# Integrated transcriptomic and metabolomic analyses reveal host–metabolite interactions underlying cloacal inflammation in laying Pekin ducks

**DOI:** 10.5713/ab.260208

**Published:** 2026-04-16

**Authors:** Yaxi Xu, Wentao Cai, Qinglei Yang, Zhenlin Liu, Xihui Sheng, Shuisheng Hou, Jian Hu

**Affiliations:** 1 College of Animal Science and Technology, Beijing University of Agriculture, Beijing, China; 2 Institute of Animal Science, Chinese Academy of Agricultural Sciences, Beijing, China; 3 Shandong Zhongxin Food Group Co., Ltd., Binzhou, China

**Keywords:** Cloacal Inflammation, Laying Duck, Metabolomics, Transcriptomics

## Abstract

**Objective:**

Cloacal inflammation in laying ducks, often accompanied by prolapse, is a major threat to animal welfare, productivity, and intestinal health. However, the underlying molecular and metabolic mechanisms remain largely unclear.

**Methods:**

Cloacal tissues and contents from inflamed and healthy laying Pekin ducks were examined histologically, transcriptomically, and metabolomically. Cross-omics integration was used to identify coordinated molecular and metabolic changes.

**Results:**

Hematoxylin and eosin staining showed a loose and disorganized structure of the cloacal sphincter in inflamed ducks. Transcriptomic analysis identified differentially expressed genes that are enriched in immune signaling, inflammatory response, and epithelial repair pathways. Metabolomic profiling revealed significant alterations in taurine- and lipid-related metabolic pathways. Integrated analysis highlighted coordinated changes between immune-related genes (*COL6A3*, *SPARC*, *PPEF1*, *HSD17B2*, and *KCNG2*) and key metabolites including Hydroxytyrosol-Ac, Phytol, GG-alcohol, MeCys-SO, Thiazoline, and PEA-glu.

**Conclusion:**

The findings provide a multi-omics perspective on cloacal inflammation in laying Pekin ducks, highlighting host–metabolite interactions that may play a role in immune activation and epithelial barrier dysfunction.

## INTRODUCTION

Poultry production serves as a major global source of animal protein, and white-feathered meat ducks are extensively reared in China due to their rapid growth, high feed efficiency, and superior meat quality [1]. However, the intensification of duck farming has introduced several production challenges—including environmental stressors, pathogenic microbial infections, and immune dysregulation, which collectively compromise growth performance and overall productivity [2]. Cloacal inflammation is a common disease in meat ducks, often presenting as redness, erosion, prolapse, and secondary infections [3]. Beyond animal welfare concerns, these symptoms can also impair excretion and reproductive functions, indirectly impacting production performance [4].

Research on cloacal inflammation in poultry has so far focused mainly on describing clinical symptoms and developing preventive or therapeutic strategies [4–6]. Previous studies have suggested associations with gut microbiota disturbances, environmental factors, and altered immune responses in ducks [7–9]; however, the underlying molecular mechanisms within the host remain largely unclear. The cloaca, which serves as a shared opening for the intestinal, urinary, and reproductive tracts, relies on a finely tuned balance of gene regulation, immune signaling, and metabolic homeostasis [10]. Increasing evidence indicates that metabolic alterations are often accompanied by changes in immune-related gene expression, suggesting that host–metabolite interactions play a key role in driving inflammation [11,12]. Nevertheless, systematical investigations of these interactions in meat ducks are still scarce.

Recent advances in omics technologies provide powerful tools for investigating complex biological processes (BPs) [13]. Transcriptomics offers insight into gene expression and regulatory networks, whereas metabolomics captures the dynamic metabolic state of tissues. Integrating these datasets enables the identification of host–metabolite interactions and provides a more comprehensive understanding of the molecular processes underlying inflammation. Multi-omics approaches have been widely applied in animal studies to investigate intestinal health [14], metabolism [15,16], and immune regulation [7]. However, these approaches have not yet been applied to the study of cloacal inflammation in ducks.

In this study, we combined transcriptomic and metabolomic analyses of healthy and cloacal-inflamed laying Pekin ducks to investigate host–metabolite interactions associated with cloacal inflammation. By identifying key genes, differential metabolites (DMs), and related pathways, we aimed to elucidate the molecular mechanisms driving cloacal inflammation and to provide new insights into cloacal health and inflammatory regulation, as well as potential targets for disease prevention and genetic improvement in meat ducks.

## MATERIALS AND METHODS

### Animals

All experimental procedures and animal welfare practices were conducted in accordance with the Guide for the Care and Use of Laboratory Animals. The samples for this study were obtained from 60-week-old female Pekin ducks from Shandong Zhongxin Food Group Company, which were raised on the ground at a stocking density of approximately 5 ducks per square meter, with uniform feeding, lighting, and free access to water.

### Cloacal examination and samples collection

Prior to sample collection, all ducks (n = 1,500) were examined for cloacal abnormalities. The external appearance of the cloaca was observed under natural conditions, including the degree of eversion and skin congestion. Subsequently, gentle manual pressure was applied to the pericloacal skin under identical conditions to observe changes in cloacal eversion and recovery. The prevalence of cloacal prolapse in this population was approximately 7.21%. For comparative analysis, ten ducks diagnosed with cloacal inflammation and ten clinically healthy ducks were randomly selected to constitute the case group and control group, respectively.

After 12 hours of feed deprivation, the ducks were euthanized by cervical dislocation following anesthesia. Immediately, sterile swabs were used to wipe the mucosal surface, and the adherent substances were collected. The swabs were then immediately placed in RNase/DNase-free cryovials with labels and rapidly frozen in liquid nitrogen, and then stored at −80°C. Skin tissues from the inflamed areas of the cloaca in diseased ducks (ID), the non-inflamed areas of the cloaca in diseased ducks (NID), as well as cloacal skin tissues from healthy ducks (HC), were collected. One portion of each sample was rapidly frozen in liquid nitrogen and stored at −80°C, while the other portion was immediately fixed in 4% formaldehyde.

### Histological sectioning and hematoxylin and eosin staining

Cloacal sphincter muscle tissues were collected from both healthy and diseased ducks and immediately fixed in 4% formaldehyde for 48 hours. Fixed tissues were dehydrated, embedded in paraffin, and sectioned at 5 μm thickness. Sections were stained with hematoxylin and eosin (H&E) following standard procedures to evaluate muscle fiber morphology, bundle organization, and inter-fiber spacing (Figures 1E, 1F).

### RNA sequencing data acquisition

#### RNA extraction, library preparation, and sequencing

For RNA sequencing (RNA-seq), 10 samples per group (ID, NID, HC) were used (n = 10). Total RNA was extracted from 30 skin tissue samples with the RNA TRIzol (Takara Bio) according to the manufacturer’s protocol. The quality and concentration of RNA were evaluated with NanoDrop 2000 (Thermo Fisher Scientific). RNA-seq libraries were constructed with the NEB Next Ultra RNA Library Prep Kit (New England Biolabs) and sequenced using the Illumina NovaSeq X Plus (Illumina) with a 150 bp paired-end protocol. Each library was sequenced with a minimum data output of 6 Gb (Supplement 1).

### Transcriptome analysis

Raw RNA-seq reads were processed using fastp 0.23.2 [17] for quality control (QC) and adapter trimming with default parameters: fastp automatically detected and removed adapter sequences, trimmed low-quality bases (Phred quality score< 20) from the read tails, and discarded reads shorter than 15 bp after trimming. Reads with more than 40% low-quality bases (Q<15) or containing over 5 ambiguous bases (N) were filtered out. PolyG tail trimming was automatically enabled for Illumina NovaSeq data.

The trimmed RNA-seq reads were then mapped against the IASCAAS_PekinDuck_T2T reference genome ( https://www.ncbi.nlm.nih.gov/assembly/GCF_047663525.1/) using HISAT2 (v2.2.1) [17] with the default parameters. Afterwards, gene-level counts were generated using featureCounts (v2.1.1) [18] and normalized using the transcripts per million (TPM) method with the R package Subread (v2.1.1) [19].

To ensure data quality and consistency among samples, unsupervised hierarchical clustering and principal component analysis (PCA) were performed using the hclust function in stats (v4.3.3) and FactoMineR (v2.11) [20] packages in R. Samples identified as outliers or potentially contaminated were removed before further analysis.

Differential expression analysis was performed using DESeq2 (v1.42.1) [21] with criteria of |log_2_FoldChange|>1 and adjusted p<0.05. Differentially expressed genes (DEGs) from the comparisons were ranked by log_2_ fold change. For each comparison, the top 50 upregulated and top 50 downregulated genes were selected. After merging and removing duplicates, the union gene set was extracted from the variance-stabilizing transformed (VST) expression matrix generated by DESeq2.

A heatmap was then constructed using the pheatmap (v 1.0.13) package in R, with Z-score normalization applied across rows. Hierarchical clustering of genes was performed, and samples were annotated according to their biological groups. To maintain the original biological grouping structure, column clustering was disabled (cluster_cols = FALSE).

Functional enrichment analysis was performed using the clusterProfiler (v4.10.1) [22] package in R. The background gene set consisted of all annotated genes detected in the RNA-Seq dataset. Gene Ontology (GO) and Kyoto Encyclopedia of Genes and Genomes (KEGG) databases were used to explore the BPs and pathways associated with DEGs. Enrichment significance was assessed using the Benjamini–Hochberg procedure, and terms with adjusted p<0.05 were considered significantly enriched. For visualization, enriched GO terms were categorized into BP, cellular component (CC), and molecular function (MF) domains. KEGG pathways were visualized using bubble plots generated in R. Only the top enriched terms were retained to highlight major BPs and metabolic pathways associated with cloacal inflammation.

### Metabolite extraction and liquid chromatography-mass spectrometry analysis

Approximately 50 mg of each sample was weighed into a 2 mL centrifuge tube containing a 6 mm stainless steel grinding bead. A total of 400 μL of extraction solvent (methanol:water = 4:1, v/v) containing internal standards (L-2-chlorophenylalanine, 0.02 mg/mL) was added. The samples were homogenized using a tissue grinder (6 min, −10°C, 50 Hz) and subsequently sonicated (30 min, 5°C, 40 kHz). After incubation at −20°C for 30 min, the mixture was centrifuged at 13,000×g for 15 min at 4°C. The supernatant was transferred into autosampler vials with inner inserts for liquid chromatography-mass spectrometry (LC–MS) analysis. QC samples were prepared by pooling equal aliquots from all extracts to assess system stability throughout the analysis.

Metabolomic profiling was performed using a Thermo Fisher Scientific ultra-high-performance liquid chromatography (UHPLC) system coupled to a Q Exactive HF-X mass spectrometer (Thermo Fisher Scientific). Chromatographic separation was achieved on an ACQUITY UPLC HSS T3 column (Waters). The mobile phases consisted of A: 95% water+5% acetonitrile with 0.1% formic acid and B: 47.5% acetonitrile+47.5% isopropanol+5% water with 0.1% formic acid. The gradient elution program was as follows: 0–2 min, 5% B; 2–8 min, 5%–50% B; 8–14 min, 50%–90% B; 14–16 min, 90% B; 16–16.1 min, 90%–5% B; and 16.1–20 min, 5% B for re-equilibration. The flow rate was 0.35 mL/min, the column temperature was maintained at 40°C, and the injection volume was 3 μL.

Mass spectrometric detection was carried out in both positive and negative electrospray ionization (ESI) modes. The parameters were set as follows: spray voltage, +3.5 kV (positive) and −3.5 kV (negative); capillary temperature, 325°C; heater temperature, 425°C; sheath gas, 50 (arb units); auxiliary gas, 13 (arb units); S-Lens RF level, 50; full MS resolution, 60,000; MS/MS resolution, 7,500; normalized collision energy, 20%, 40%, and 60%. Data acquisition and processing were performed using Xcalibur software (Thermo Fisher Scientific). QC samples were analyzed after every 5–15 injections to monitor instrument drift and analytical reproducibility.

### Differential analysis of metabolites

To explore metabolic differences between groups, orthogonal partial least squares discriminant analysis (OPLS-DA) was performed using the R package MetaboAnalystR v3 [23]. Metabolite intensities were log_2_-transformed and mean-centered before modeling. The OPLS-DA model generated score and permutation plots, and 200 permutation tests were conducted to assess model reliability and prevent overfitting.

Significantly altered metabolites were identified according to the criteria of variable importance in projection (VIP)>1 and p<0.05. These DMs were further subjected to hierarchical clustering analysis (HCA) using the hclust function in R v4.1.0, and the normalized signal intensities were visualized as clustered heatmaps with the ComplexHeatmap v1.20.2 package [23] to display metabolite abundance patterns across samples.

### Kyoto Encyclopedia of Genes and Genomes annotation and enrichment analysis

DMs were annotated using the KEGG Compound database (https://www.kegg.jp/kegg/compound/) and mapped to the KEGG Pathway database (https://www.kegg.jp/kegg/pathway.html). Pathway enrichment analysis was conducted using the minimum hypergeometric test, and pathways with adjusted p<0.05 were considered significantly enriched.

### Integrated transcriptomic and metabolomic analysis

To explore the molecular interactions between transcriptional regulation and metabolic changes, integrated analysis of transcriptomic and metabolomic datasets was performed. DEGs and DMs were identified according to the respective screening criteria. Normalized transcript expression (TPM) and metabolite abundance data were used to calculate pairwise Pearson correlation coefficients (PCCs). Correlations with |r|>0.6 and p<0.05 were considered statistically significant.

The significant DEG–DM pairs were imported into Cytoscape v3.9.1 to construct the gene–metabolite interaction network. Network enrichment and visualization were carried out using the MetScape [24] and ClueGO [25] plugins, based on the KEGG and GO databases, respectively. These analyses enabled the identification of co-regulated pathways and key regulatory nodes potentially linking gene expression and metabolite variation.

## RESULTS

### Phenotypic and histological characteristics of cloacitis in ducks

Compared with healthy ducks, ducks with cloacitis exhibited obvious cloacal abnormalities at the phenotypic level. Under natural conditions, the cloaca of diseased ducks showed abnormal appearance and eversion, whereas the cloaca of healthy ducks remained morphologically normal (Figures 1A, 1B). When gentle manual pressure was applied to the pericloacal skin under identical conditions, the cloaca of healthy ducks remained largely stable, and any transient eversion rapidly returned to normal upon release of the pressure. In contrast, diseased ducks exhibited persistent or aggravated cloacal eversion, with delayed recovery after removal of the force, and a few severely affected individuals failed to return to the normal state, indicating impaired cloacal sphincter function (Figures 1C, 1D).

Histological examination further revealed pronounced structural abnormalities in the cloacal sphincter muscle of diseased ducks. H&E staining showed that muscle fibers in healthy ducks were regularly shaped, relatively uniform in size, and tightly and orderly arranged into bundles. In contrast, diseased ducks exhibited markedly disorganized muscle fibers with irregular boundaries and widened inter-fiber spaces, indicating structural damage to the cloacal sphincter muscle (Figures 1E, 1F).

### Transcriptomic analysis of cloacal skin tissues

RNA-seq generated high-quality datasets for all skin samples, with detailed sequencing metrics summarized in Supplement 1. PCA of the transcriptomic data did not show distinct clustering among the three groups (ID; NID; and healthy controls, HC; Figure 2A), suggesting that overall transcriptomic variation was modest relative to within-group variability. Although PCA did not show clear separation, pairwise differential expression analysis revealed substantial changes in gene expression: 436 upregulated and 451 downregulated genes in ID vs NID (Figure 2B; Supplement 2), 315 upregulated and 422 downregulated genes in ID vs HC (Figure 2C; Supplement 3), and 532 upregulated and 1,002 downregulated genes in NID vs HC (Figure 2D; Supplement 4). Heatmaps of the top 50 DEGs in each comparison illustrated distinct expression patterns among the three groups (Figure 2E).

GO enrichment analyses were performed for each set of DEGs. In the comparison between inflamed (ID) and non-inflamed (NID) cloacal skin of diseased ducks, the 887 DEGs were significantly enriched in 55 GO terms, including 40 BPs (e.g., immune system process, immune response, defense response, inflammatory response, positive regulation of immune system process, positive regulation of response to biotic stimulus, positive regulation of defense response, positive regulation of response to external stimulus, regulation of innate immune response, and positive regulation of innate immune response), 2 CCs (eg., extracellular space), and 13 molecular functions (eg., cytokine activity) (Figure 2F; Supplement 5). For the comparison between inflamed skin of diseased ducks (ID) and HC, 737 DEGs were enriched in 51 GO terms, including 41 BPs (e.g., immune response, immune system process, inflammatory response, activation of innate immune response, pattern recognition receptor signaling pathway, toll-like receptor signaling pathway, activation of immune response, immune response-activating signaling pathway, innate immune response-activating signaling pathway, and immune response-regulating signaling pathway), 2 CCs (extracellular region and extracellular space), and 8 molecular functions (e.g., cytokine activity) (Figure 2G; Supplement 6). In NID compared with HC, 1,534 DEGs were enriched in 13 GO terms, all corresponding to BPs, including immune system process, immune response, positive regulation of BP, regulation of immune system process, positive regulation of immune system process, inflammatory response, small GTPase-mediated signal transduction, positive regulation of response to biotic stimulus, positive regulation of defense response, and positive regulation of response to external stimulus (Figure 2H; Supplement 7).

KEGG pathway analysis revealed that DEGs in ID vs NID were enriched in pathways including cytoskeleton in muscle cells, cytokine–cytokine receptor interaction, motor proteins, intestinal immune network for IgA production, calcium signaling pathway, C-type lectin receptor signaling pathway, Toll-like receptor signaling pathway, adrenergic signaling in cardiomyocytes, cardiac muscle contraction, phagosome, apelin signaling pathway, ECM–receptor interaction, efferocytosis, Salmonella infection, cell adhesion molecules, Influenza A, and adipocytokine signaling pathway (Figure 2I; Supplement 8). In ID vs HC, 737 DEGs were enriched in cytokine–cytokine receptor interaction, cytoskeleton in muscle cells, ECM–receptor interaction, cornified envelope formation, Toll-like receptor signaling pathway, focal adhesion, intestinal immune network for IgA production, C-type lectin receptor signaling pathway, arachidonic acid metabolism, cell adhesion molecules, and phagosome (Figure 2J; Supplement 9). In NID vs HC, 1,534 DEGs were enriched in intestinal immune network for IgA production, cytoskeleton in muscle cells, cell adhesion molecules, cytokine–cytokine receptor interaction, motor proteins, phagosome, cardiac muscle contraction, adrenergic signaling in cardiomyocytes, cell cycle, C-type lectin receptor signaling pathway, DNA replication, calcium signaling pathway, herpes simplex virus 1 infection, and nitrogen metabolism (Figure 2K; Supplement 10).

To identify key genes commonly involved in cloacal inflammation, we examined the overlap of DEGs among the three comparisons (ID vs NID, NID vs HC, and ID vs HC). A total of 54 DEGs were shared across all groups (Figure 3A). To explore common and unique functional enrichments, we performed GO and KEGG analyses for each comparison individually and summarized the overlaps using Venn diagrams. The GO enrichment Venn diagram (Figure 3B) shows 12 GO terms commonly enriched among the three comparisons, while the KEGG enrichment Venn diagram (Figure 3C) highlights six pathways shared across the comparisons. We then analyzed the expression trends of the 54 shared DEGs across the three groups. Most genes exhibited a similar pattern, with the highest expression in HC, the lowest in NID, and intermediate expression in ID (Figure 3D). The 12 GO terms commonly enriched among the three comparisons were all BPs, including immune system process, immune response, inflammatory response, defense response, positive regulation of immune system process, positive regulation of response to biotic stimulus, positive regulation of defense response, positive regulation of response to external stimulus, regulation of innate immune response, positive regulation of innate immune response, regulation of immune system process, and positive regulation of BP (Figure 3E). And the 6 KEGG pathways shared among the DEG sets of three comparisons consisted of cytoskeleton in muscle cells, Cytokine-cytokine receptor interaction, intestinal immune network for lgA production, C-type lectin receptor signaling pathway, phagosome, and cell adhesion molecules (Figure 3F).

### Metabolomic profiling of cloacal contents

Subsequently, metabolomic profiling was performed on cloacal contents from diseased and healthy ducks, resulting in the detection of 8,101 metabolites, of which 921 were annotated. Among the annotated metabolites, 29.10% were classified as lipids and lipid-like molecules, 23.78% as organic acids and derivatives, 18.78% as organoheterocyclic compounds, 9.34% as benzenoids, 8.79% as organic oxygen compounds, 3.80% as phenylpropanoids and polyketides, 2.93% as nucleosides, nucleotides, and analogues, 1.95% as organic nitrogen compounds, 0.87% as alkaloids and derivatives, 0.43% as hydrocarbons, 0.11% as lignans, neolignans, and related compounds, and 0.11% as hydrocarbon derivatives (Figure 4A; Supplement 11).

OPLS-DA analysis using all 8,101 metabolites revealed clear separation between diseased and healthy ducks, as shown in the score plot (Figure 4B), indicating distinct metabolic profiles in cloacal contents. DM analysis with VIP>1 and p<0.05 identified 152 upregulated and 106 downregulated metabolites in diseased ducks compared with HC (Figure 4C; Supplement 12). Among these 258 DMs, only 24 were annotated, and their abundance across samples is displayed in a heatmap (Figure 4D), showing significant differences between the two groups.

Pathway enrichment analysis of the 24 annotated DMs revealed significant involvement in Taurine and hypotaurine metabolism, and Caffeine metabolism.

### Integration of transcriptomic and metabolomic datasets

To explore cross-layer molecular interactions, DEGs (p<0.05, |log2FC|>1) were correlated with DMs identified using a relaxed threshold (p<0.1, VIP>0.8). Using p<0.05 and |r|>0.6 as significance criteria, 313 gene–metabolite pairs were identified, involving 123 DEGs and 59 metabolites (Figure 5A). Correlation details are listed in Supplement 13. Network analysis in Cytoscape highlighted several key metabolites—Hydroxytyrosol-Ac, Phytol, GG-alcohol, MeCys-SO, Thiazoline, and PEA-glu—interacting with immune-related genes such as *COL6A3*, *SPARC*, *PPEF1*, *HSD17B2*, and *KCNG2* (Figure 5B).

Functional network enrichment of the interacting gene–metabolite nodes indicated involvement in arachidonic acid metabolism, extracellular matrix remodeling, metalloendopeptidase activity, and myeloid leukocyte activation, linking metabolic perturbations with inflammatory gene programs (Figure 5C).

## DISCUSSION

Cloacal inflammation in laying ducks can progress to cloacal prolapse, leading to increased mortality and reduced reproductive performance, and therefore represents an important disease requiring control in duck production systems [4]. Previous studies have indicated that mechanical injury, microbial infection, and environmental stress may all induce cloacal inflammation, accompanied by local immune responses and structural tissue alterations [5]. Repeated expansion of the cloacal sphincter during egg laying may compromise mucosal barrier integrity, thereby increasing the risk of microbial invasion [5,6]; colonization by opportunistic pathogens can further trigger local inflammatory responses [8]; and environmental stress may indirectly promote inflammation by impairing immune function [8]. However, previous studies have largely focused on clinical symptoms and histopathological observations [5,26,27], and systematic analyses of the underlying molecular regulatory mechanisms remain limited, particularly for this specific physiological stage in laying ducks, where integrated multi-omics investigations are lacking.

Transcriptomic analysis showed that a large number of DEGs were identified between the non-inflamed region and HC, exceeding those observed in comparisons between inflamed and non-inflamed regions and between inflamed regions and HC. This indicates that substantial transcriptional alterations had already occurred in adjacent tissues without visible lesions, suggesting that cloacal inflammation is not confined to the local lesion but may involve a local field effect extending to neighboring tissues. Similar phenomena have been reported in other mucosal inflammatory diseases, in which adjacent tissues exhibit immune-related gene expression changes before morphological alterations become apparent [28], thereby contributing to the expansion of inflammation [14,15,29]. Accordingly, the non-inflamed region may represent a transitional state during disease progression, whereas the inflamed region exhibits stronger local immune and tissue responses. This gradient pattern suggests that cloacal inflammation may be initiated by local tissue injury and subsequently expand through immune pre-activation and microenvironmental alterations in adjacent tissues, representing a progressive process from neighboring tissue pre-activation to amplified inflammation within the lesion.

Further comparison between inflamed and non-inflamed regions revealed that multiple inflammation-related genes were further upregulated in the inflamed area, indicating that local inflammatory responses were markedly amplified within lesion sites. These genes were primarily associated with immune regulation, extracellular matrix remodeling, and oxidative stress pathways, suggesting that inflamed regions exhibit not only immune activation but also structural tissue changes and metabolic dysregulation. Similar inflammatory amplification effects have been observed in mucosal inflammation and tissue injury models, highlighting the importance of local inflammatory microenvironment changes in disease progression [15,30,31].

Histological observations further supported these findings. In inflamed individuals, cloacal sphincter tissues exhibited disorganized muscle fiber arrangement and widened inter-fiber spaces, suggesting structural damage and possible inflammatory infiltration. These structural alterations are consistent with enhanced inflammatory responses [32]. Transcriptomic results also revealed differential expression of genes involved in extracellular matrix remodeling, including members of the ADAM and ADAMTS families. These metalloproteinases can mediate extracellular matrix degradation and reconstruction during inflammatory injury and tissue repair, thereby altering tissue structure and mechanical properties [33,34]. The observed widening of muscle fiber spaces and tissue disorganization may therefore be associated with enhanced extracellular matrix degradation, suggesting that cloacal inflammation involves not only immune responses but also significant tissue remodeling.

Metabolomic analysis indicated pronounced metabolic alterations associated with cloacal inflammation, particularly involving taurine and hypotaurine metabolism and lipid-related metabolic pathways. Taurine plays an important role in maintaining mucosal barrier integrity and mitigating oxidative stress–induced damage [35], and changes in its metabolism may reflect altered local antioxidant capacity during inflammation [36,37]. In addition, lipid metabolism plays a critical role in inflammatory regulation, and lipid mediators derived from arachidonic acid metabolism can participate in inflammatory responses and tissue repair processes [38,39]. These findings suggest that oxidative stress–related metabolic changes and lipid metabolic abnormalities accompany immune-related gene expression changes, indicating that cloacal inflammation involves both lipid inflammatory signaling and altered antioxidant regulation.

Integrated transcriptomic and metabolomic analyses further revealed coordinated regulation between genes and metabolites. Gene–metabolite modules enriched in arachidonic acid metabolism and extracellular matrix remodeling suggest that inflammatory responses, structural tissue changes, and metabolic regulation may form coordinated regulatory networks. Such coordinated transcriptomic and metabolomic alterations have been reported in various inflammatory diseases [15,40,41], indicating that multi-layered molecular regulation plays an important role in the development and progression of inflammation. Collectively, changes in immune and extracellular matrix remodeling genes at the transcriptomic level correspond with alterations in lipid inflammatory and antioxidant metabolic pathways, suggesting that cloacal inflammation may be driven by combined effects of structural tissue damage, inflammatory signal amplification, and metabolic dysregulation, reflecting coordinated cross-omics regulation.

It should also be noted that cloacal inflammation may arise from multiple factors, including mechanical injury during egg laying, microbial infection, and environmental stress. Although different etiologies may produce similar phenotypes, their immune responses and metabolic regulatory mechanisms may differ [15,40]. Previous studies have shown that alterations in gut morphology and microbiota are closely associated with immune responses and physiological changes in ducks. For example, changes in stocking density significantly affected gut morphology, microbiota composition, and immune-related physiological indicators in ducks, highlighting the link between intestinal health and inflammatory responses [42]. Recent studies have also demonstrated that different enterotypes are associated with growth performance and carcass traits in ducks, highlighting the interaction between microbiota composition and host physiology [43]. Therefore, the molecular changes observed in this study may reflect the combined effects of multiple causative factors. Despite this potential heterogeneity, immune responses, extracellular matrix remodeling, and lipid metabolism pathways were consistently enriched across comparisons, suggesting that these processes may represent shared regulatory mechanisms underlying cloacal inflammation.

In summary, cloacal inflammation in laying ducks involves neighboring tissue pre-activation, local inflammatory amplification, extracellular matrix remodeling, and lipid metabolic alterations, and is characterized by coordinated transcriptomic and metabolomic regulation (Figure 6). These findings suggest that cloacal inflammation is not a single inflammatory event but rather the result of combined structural, immune, and metabolic alterations, providing new multi-omics evidence for understanding the molecular regulatory mechanisms underlying cloacal inflammation in laying ducks.

## CONCLUSION

In conclusion, this study provides an integrated molecular characterization of cloacal inflammation in laying ducks. Both inflamed and adjacent non-inflamed cloacal skin exhibited immune activation, while metabolomic alterations highlighted disruptions in taurine- and lipid-related pathways. Cross-omics integration identified key gene–metabolite interactions, such as *COL6A3* and Hydroxytyrosol-Ac, linking inflammation, oxidative stress, and tissue remodeling. These insights improve our understanding of the molecular basis of cloacal inflammation and prolapse, and offer a foundation for improved health management, early intervention strategies, and the identification of candidate biomarkers in laying duck production systems.

## Figures and Tables

**Figure 1:**
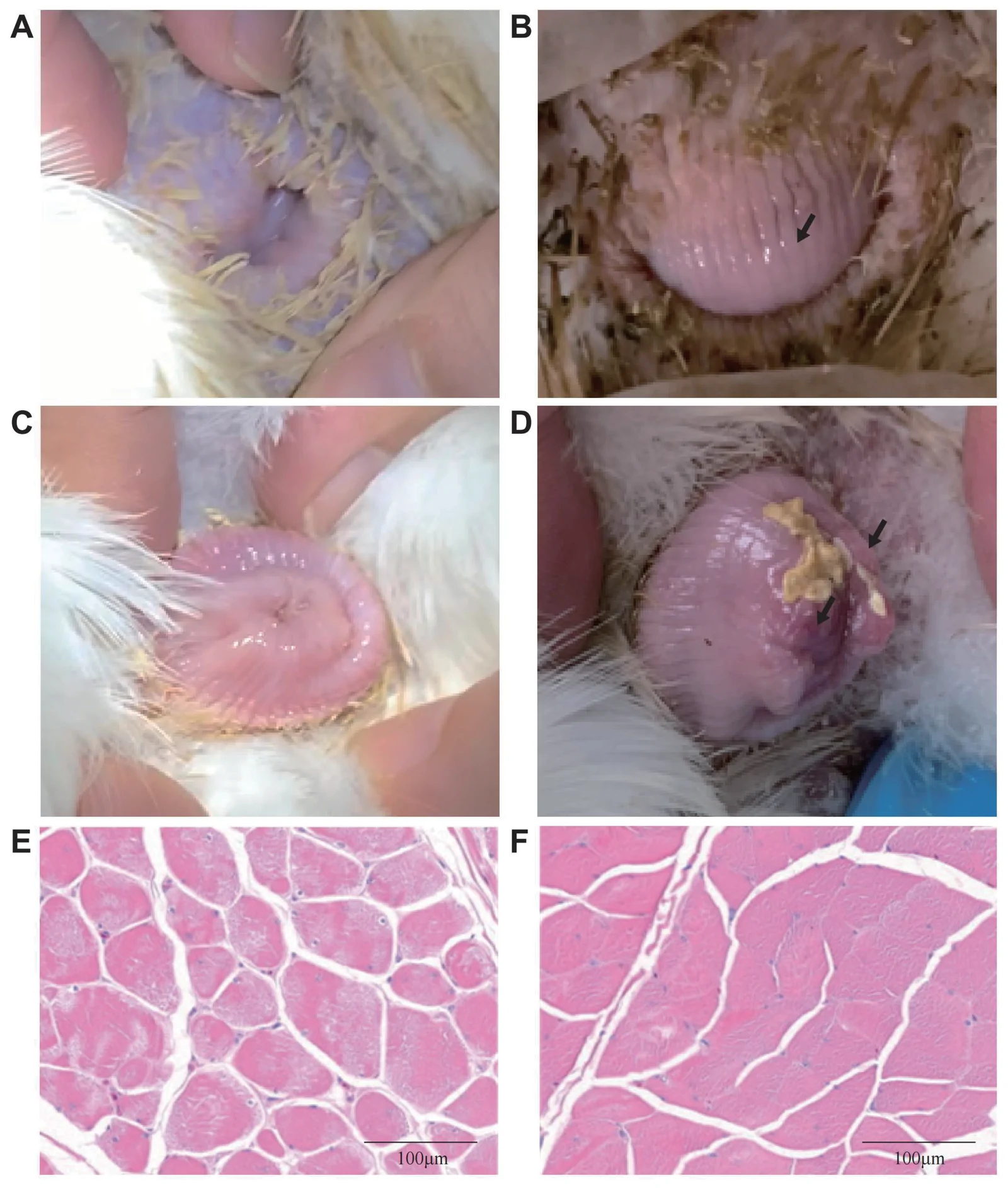
Phenotypic and histological characterization of cloacitis in ducks. (A, B) Representative images of the cloaca from healthy ducks (A) and ducks with cloacitis (B) under natural conditions. Diseased ducks exhibited cloacal eversion, mucosal hyperemia, and swelling (arrows). (C, D) Cloacal appearance of healthy (C) and diseased ducks (D) after gentle manual pressure applied to the pericloacal skin under identical conditions. Healthy ducks maintained a stable cloacal structure, whereas diseased ducks showed aggravated eversion, indicating impaired cloacal sphincter function. (E, F) Hematoxylin and eosin (H&E) staining of cloacal sphincter muscle sections from healthy (E) and diseased ducks (F). Diseased tissues showed disorganized muscle fibers and widened inter-fiber spaces. Scale bar = 100 μm.

**Figure 2:**
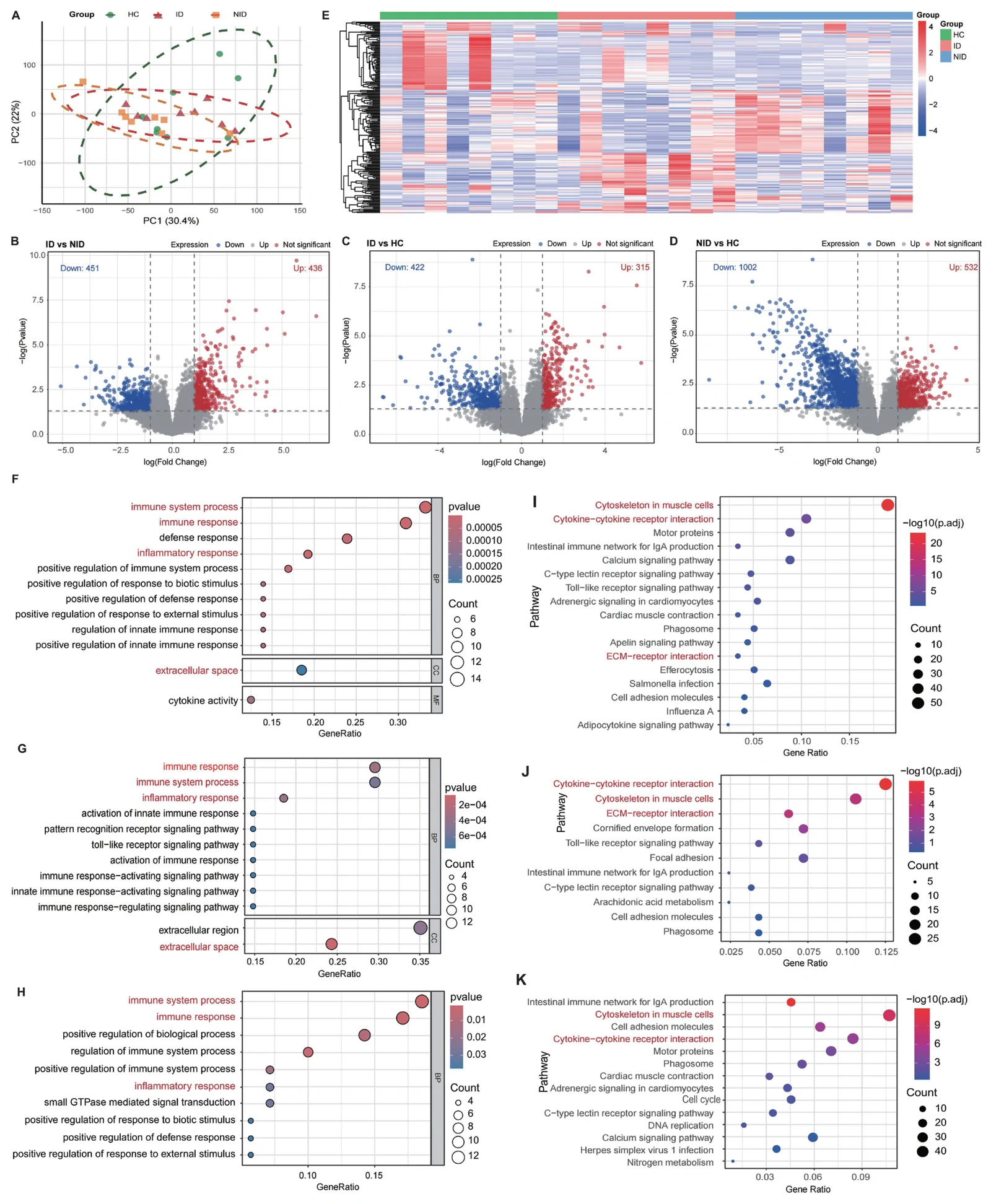
Transcriptomic profiling of cloacal skin from healthy ducks (HC) and ducks with cloacal inflammation, including inflamed (ID) and non-inflamed (NID) sites. (A) Principal component analysis (PCA) based on normalized transcripts per million (TPM) values showing group separation among groups. (B–D) Volcano plots of differentially expressed genes (DEGs) for ID vs NID (B), ID vs HC (C), and NID vs HC (D). Red dots indicate upregulated genes and blue dots indicate downregulated genes (|log2FC|>1, p<0.05). (E) Heatmap of top DEGs across the three groups based on normalized expression values. (F–H) Gene Ontology (GO) enrichment of DEGs in each comparison (top enriched terms shown). (I–K) Kyoto Encyclopedia of Genes and Genomes (KEGG) pathway enrichment of DEGs in each comparison.

**Figure 3:**
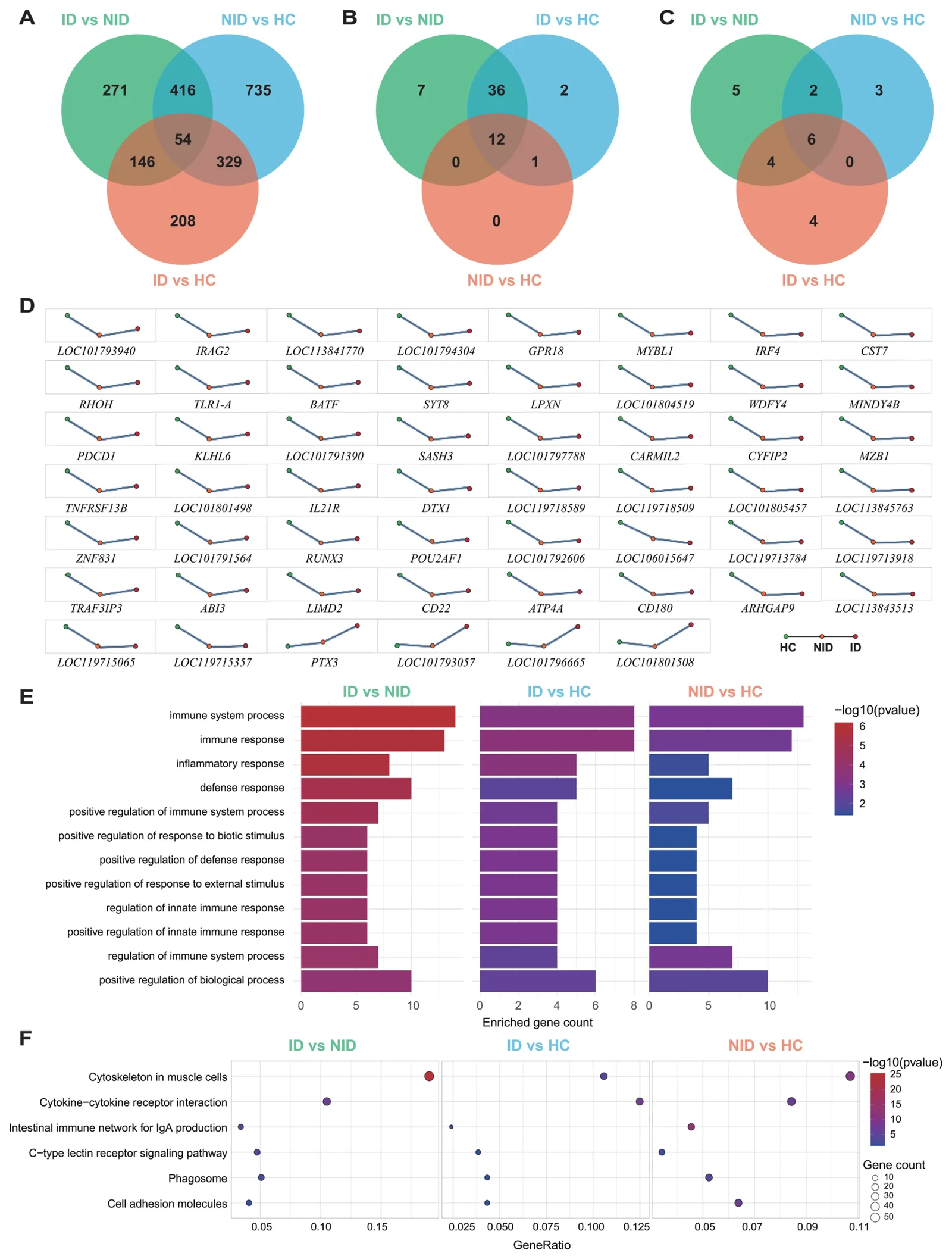
Intersection and functional enrichment analysis of differentially expressed genes (DEGs). (A) Venn diagram showing overlap of DEGs among the three comparison groups (|log_2_FC|>1, p<0.05). (B) Venn diagram of shared Gene Ontology (GO) terms across the three DEG sets. (C) Venn diagram of the shared Kyoto Encyclopedia of Genes and Genomes (KEGG) pathways across the three DEG sets. (D) Expression trend of the 54 intersection genes across the three groups based on TPM values. (E) GO enrichment analysis of the 54 intersection genes (p<0.05). Bar color represents −log_10_(p) values and bar length indicates gene counts. (F) KEGG enrichment analysis of the 54 intersection genes (p<0.05). Dot color represents −log_10_(p) values, dot size indicates gene counts, and the x-axis shows the ratio of enriched genes to the total number of genes annotated in each pathway.

**Figure 4:**
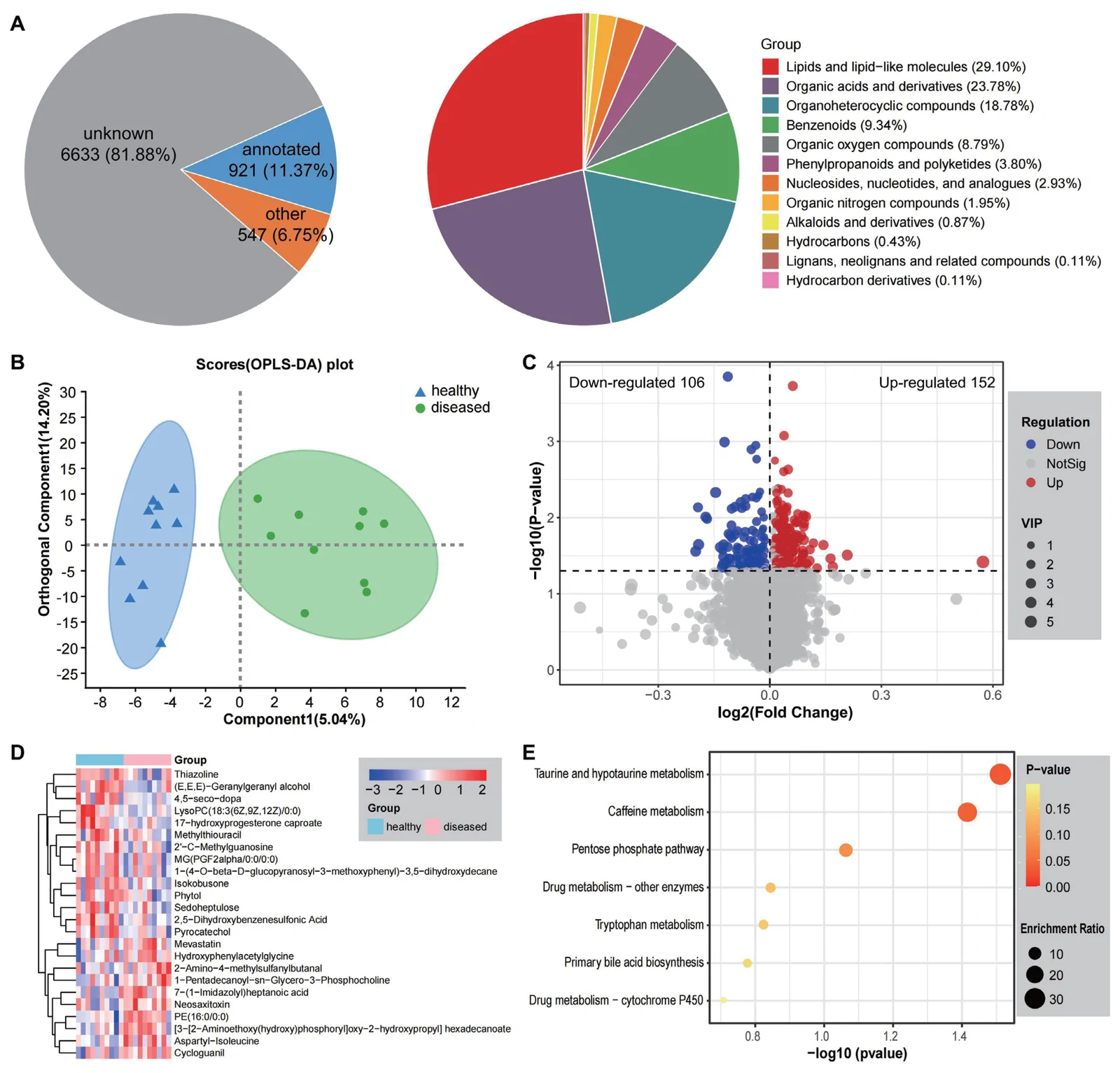
Metabolomic profiling of cloacal contents reveals metabolic alterations between diseased and healthy ducks. (A) Pie charts showing the total metabolites detected (8,101 in total, including 921 annotated compounds) and composition of annotated metabolites. (B) OPLS-DA score plot showing distinct clustering between groups. (C) Volcano plot of differential metabolites in cloacal contents between diseased and healthy ducks (VIP>1, p<0.05). (D) Heatmap of annotated differential metabolites. (E) KEGG pathway enrichment of differential metabolites. OPLS-DA, orthogonal partial least squares discriminant analysis; VIP, variable importance in projection; KEGG, Kyoto Encyclopedia of Genes and Genomes.

**Figure 5:**
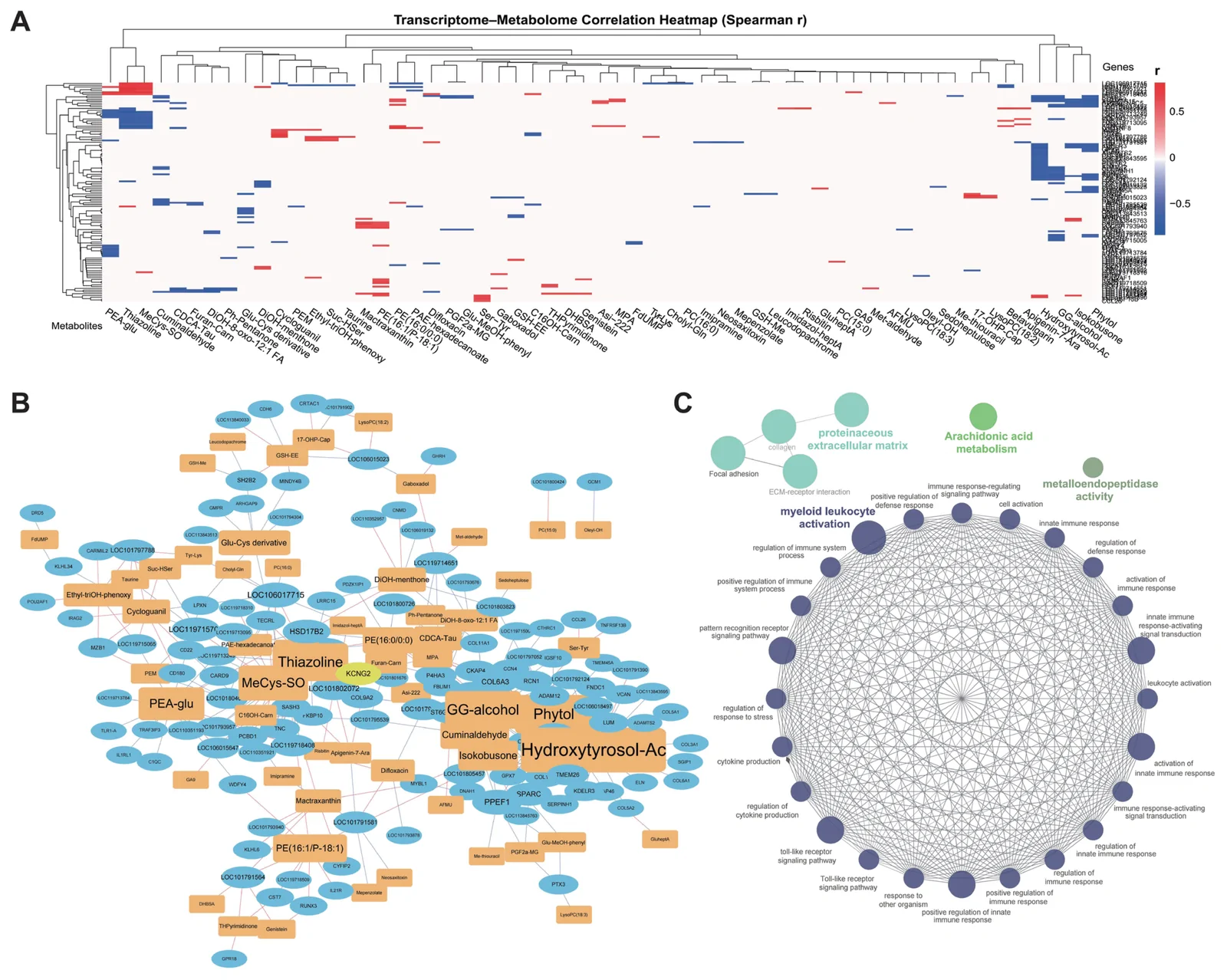
Integrated transcriptome–metabolome analysis of the cloacal samples. (A) Correlation heatmap showing Spearman correlations between differential genes and metabolites (|r|>0.6, p<0.05). (B) Network of significant gene–metabolite pairs highlighting key nodes such as *Hydroxytyrosol-Ac* and immune-related genes (*HSD17B2*, *SPARC*, *COL6A3*). (C) Functional enrichment network showing that correlated genes and metabolites were mainly involved in immune defense, extracellular matrix remodeling, and arachidonic acid metabolism.

**Figure 6:**
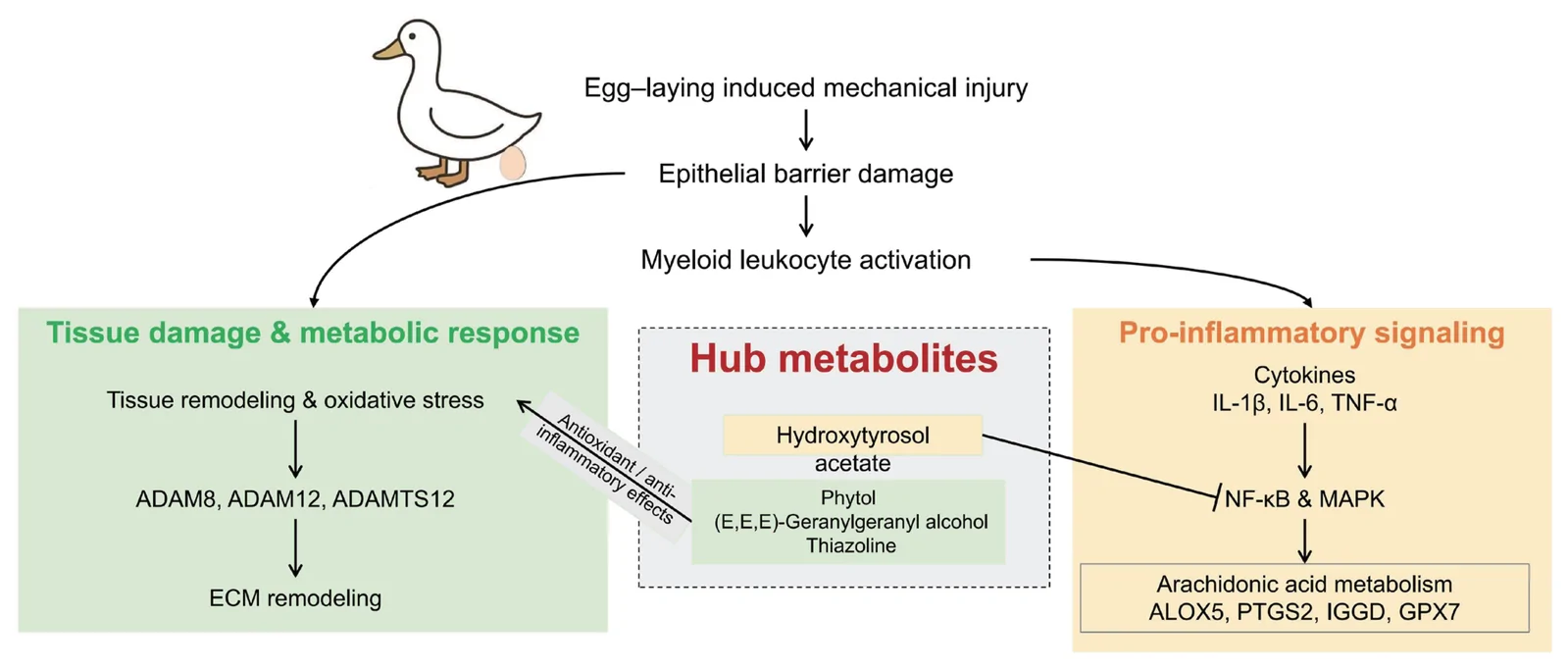
Proposed mechanism of inflammation, oxidative stress, and metabolic regulation in cloacal injury of laying Pekin ducks. The schematic summarizes transcriptomic and metabolomic findings from this study. Differentially expressed genes and metabolites suggest activation of immune responses, extracellular matrix remodeling, and lipid metabolism pathways, contributing to cloacal tissue injury. Solid arrows indicate relationships supported by transcriptomic and metabolomic results in this study. Dashed arrows indicated proposed mechanisms inferred from previous studies. Arrows indicate activation or promotion, whereas T-shaped bars indicate inhibition.

## Data Availability

The dataset supporting the conclusions of this article is available with links to BioProject accession number PRJNA1433160 (https://www.ncbi.nlm.nih.gov/bioproject/PRJNA1433160).

## SUPPLEMENTARY MATERIAL

**Supplemant 1**. Summary of RNA-seq data quality and alignment statistics for all cloacal skin samples.

**Supplement 2**. Significantly differentially expressed genes in ID vs NID comparison.

**Supplement 3**. Significantly differentially expressed genes in ID vs HC comparison.

**Supplement 4**. Significantly differentially expressed genes in NID vs HC comparison.

**Supplement 5**. GO enrichment analysis of DEGs in ID vs NID.

**Supplement 6**. GO enrichment analysis of DEGs in ID vs HC.

**Supplement 7**. GO enrichment analysis of DEGs in NID vs HC.

**Supplement 8**. KEGG pathway enrichment of DEGs in ID vs NID.

**Supplement 9**. KEGG pathway enrichment of DEGs in ID vs NID.

**Supplement 10**. KEGG pathway enrichment of DEGs in ID vs NID. **Supplement 11**. Classification of detected metabolites in the cloacal contents of diseased and healthy ducks.

**Supplement 12**. Differential metabolites in cloacal contents of ducks with cloacal inflammation (ID) versus healthy controls (HC).

**Supplement 13**. Correlation analysis between transcriptomic DEGs and differential metabolites.
