# Supplementary material for: Integrated transcriptomic and metabolomic analyses reveal host–metabolite interactions underlying cloacal inflammation in laying Pekin ducks
Source: Anim Biosci. 2026 Apr 16;39(10):260208. doi: 10.5713/ab.260208 (PMC13662949; doi:10.5713/ab.260208)
Supplement: Supplementary file 1 [file ab-260208-Supplementary-1.pdf]

Supplemant 1. Summary of RNA-seq data quality and alignment statistics for all cloacal skin samples

ID	Group	Raw Reads	Raw Base(G)	Clean Reads	Clean Base(G)	Clean Reads Rate(%)	Raw Q30 Bases Rate(%)	Clean Q30 Bases Rate(%)	Raw GC percent(%)	Clean GC percent(%)	mapping rate (%)	uniq mapping rate (%)
HC1	HC (Healthy control)	22,851,867	6.86	22,055,427	6.62	96.51	94.36	94.28	54.13	54.17	89.17	77.88
HC2	HC (Healthy control)	24,175,523	7.25	22,151,103	6.65	91.63	95.55	95.43	55.14	55.33	87.36	76.36
HC3	HC (Healthy control)	24,043,002	7.21	22,982,878	6.89	95.59	94.42	94.31	54.19	54.25	87.79	77.32
HC4	HC (Healthy control)	24,032,962	7.21	23,144,995	6.94	96.31	94.50	94.41	52.38	52.41	90.81	80.85
HC5	HC (Healthy control)	26,829,351	8.05	22,167,110	6.65	82.62	96.02	95.81	56.26	56.76	85.08	73.54
HC6	HC (Healthy control)	22,571,872	6.77	22,034,496	6.61	97.62	94.26	94.21	52.22	52.23	90.81	79.88
HC7	HC (Healthy control)	23,676,405	7.10	23,222,206	6.97	98.08	93.01	92.96	50.92	50.93	91.75	81.70
HC8	HC (Healthy control)	23,474,222	7.04	22,800,434	6.84	97.13	94.20	94.13	52.24	52.26	91.00	80.42
ID1	ID (Inflamed region of deseased duck)	24,885,036	7.47	23,966,737	7.19	96.31	95.03	94.95	53.92	53.96	90.09	80.26
ID2	ID (Inflamed region of deseased duck)	23,843,401	7.15	23,099,523	6.93	96.88	93.38	93.30	53.86	53.89	88.55	77.39
ID3	ID (Inflamed region of deseased duck)	24,786,100	7.44	23,576,993	7.07	95.12	94.28	94.17	54.26	54.32	88.66	78.53
ID4	ID (Inflamed region of deseased duck)	23,553,185	7.07	22,663,706	6.80	96.22	93.92	93.81	53.93	53.96	90.04	80.64
ID5	ID (Inflamed region of deseased duck)	24,198,015	7.26	23,644,747	7.09	97.71	93.52	93.47	51.40	51.41	91.32	79.82
ID6	ID (Inflamed region of deseased duck)	22,958,556	6.89	22,193,914	6.66	96.67	93.84	93.76	53.41	53.45	90.73	81.18
ID7	ID (Inflamed region of deseased duck)	24,204,000	7.26	22,905,420	6.87	94.63	94.85	94.75	54.20	54.30	90.62	79.78
ID8	ID (Inflamed region of deseased duck)	23,212,645	6.96	22,606,320	6.78	97.39	94.20	94.15	51.53	51.55	90.83	79.70
NID1	NID (Non-inflamed region of diseased duck)	23,511,077	7.05	22,817,391	6.85	97.05	93.66	93.58	54.56	54.60	87.93	75.97
NID2	NID (Non-inflamed region of diseased duck)	22,233,734	6.67	21,399,646	6.42	96.25	94.19	94.10	53.02	53.05	89.45	77.53
NID3	NID (Non-inflamed region of diseased duck)	23,192,872	6.96	22,408,523	6.72	96.62	95.62	95.56	52.54	52.57	91.11	77.86
NID4	NID (Non-inflamed region of diseased duck)	23,310,727	6.99	22,491,688	6.75	96.49	95.53	95.47	53.62	53.66	90.72	80.12
NID5	NID (Non-inflamed region of diseased duck)	24,810,109	7.44	23,942,786	7.18	96.50	94.81	94.73	53.25	53.30	90.56	79.81
NID6	NID (Non-inflamed region of diseased duck)	23,716,678	7.12	23,132,849	6.94	97.54	94.11	94.05	52.81	52.83	90.33	80.36
NID7	NID (Non-inflamed region of diseased duck)	24,256,954	7.28	23,603,902	7.08	97.31	93.56	93.49	54.14	54.16	87.62	70.17
NID8	NID (Non-inflamed region of diseased duck)	23,866,572	7.16	23,338,370	7.00	97.79	96.31	96.28	52.27	52.28	91.87	81.08
