# Supplementary material for: Integrated transcriptomic and metabolomic analyses reveal host–metabolite interactions underlying cloacal inflammation in laying Pekin ducks
Source: Anim Biosci. 2026 Apr 16;39(10):260208. doi: 10.5713/ab.260208 (PMC13662949; doi:10.5713/ab.260208)
Supplement: Supplementary file 2 [file ab-260208-Supplementary-10,11.pdf]

Immune system	apla04625	C-type lectin receptor signaling pathway	8/208	96/5816	0.0209601	0.293441403	0.278548701	8
Signaling molecules and interaction	apla04514	Cell adhesion molecules	9/208	125/5816	0.034369189	0.416065574	0.394949464	9
Lipid metabolism	apla00590	Arachidonic acid metabolism	5/208	52/5816	0.037148712	0.416065574	0.394949464	5
Transport and catabolism	apla04145	Phagosome	9/208	133/5816	0.048040547	0.489140117	0.464315336	9

Supplement 10. KEGG pathway enrichment of DEGs in ID vs NID.

subcategory	ID	Description	GeneRatio	BgRatio	pvalue	p.adjust	qvalue	Count
Immune system	apla04672	Intestinal immune network for IgA production	20/439	34/5816	1.23E-14	1.77E-12	1.68E-12	20
NA	apla04820	Cytoskeleton in muscle cells	47/439	215/5816	1.00E-11	7.20E-10	6.84E-10	47
Signaling molecules and interaction	apla04514	Cell adhesion molecules	28/439	125/5816	1.08E-07	5.17E-06	4.91E-06	28
Signaling molecules and interaction	apla04060	Cytokine-cytokine receptor interaction	37/439	205/5816	3.82E-07	1.37E-05	1.31E-05	37
Cell motility	apla04814	Motor proteins	31/439	184/5816	1.51E-05	0.000433495	0.000411947	31
Transport and catabolism	apla04145	Phagosome	23/439	133/5816	0.000127973	0.00307136	0.00291869	23
Circulatory system	apla04260	Cardiac muscle contraction	14/439	65/5816	0.000269976	0.00555379	0.005277725	14
Circulatory system	apla04261	Adrenergic signaling in cardiomyocytes	19/439	125/5816	0.002410388	0.043386983	0.04123032	19
Cell growth and death	apla04110	Cell cycle	20/439	138/5816	0.003324249	0.053187987	0.05054414	20
Immune system	apla04625	C-type lectin receptor signaling pathway	15/439	96/5816	0.00514508	0.074089156	0.070406362	15
Replication and repair	apla03030	DNA replication	7/439	30/5816	0.005892367	0.077136438	0.07330217	7
Signal transduction	apla04020	Calcium signaling pathway	26/439	235/5816	0.030010945	0.360131341	0.342230075	26
Energy metabolism	apla00910	Nitrogen metabolism	4/439	18/5816	0.041963539	0.452454549	0.429964118	4
Infectious disease: viral	apla05168	Herpes simplex virus 1 infection	16/439	134/5816	0.043988637	0.452454549	0.429964118	16

Supplement 11. Classification of detected metabolites in the cloacal contents of diseased and healthy ducks.

Metabolite Set	Total	Hits	Ratio	Expect	P value	Holm P	FDR
Organic acids and derivatives	4600	219	23.77850163	20.4	4.98E-154	1.74E-152	1.74E-152
Organoheterocyclic compounds	6560	173	18.78393051	29	8.49E-80	2.89E-78	1.49E-78
Organic oxygen compounds	3240	81	8.794788274	14.4	2.65E-35	8.74E-34	3.09E-34
Benzenoids	5030	86	9.337676439	22.3	6.03E-26	1.93E-24	5.28E-25
Nucleosides, nucleotides, and analogues	560	27	2.931596091	2.48	1.59E-19	4.91E-18	1.11E-18
Organic nitrogen compounds	632	18	1.954397394	2.8	9.1E-10	2.73E-08	5.31E-09
Phenylpropanoids and polyketides	3830	35	3.800217155	17	0.0000665	0.00193	0.000332
Alkaloids and derivatives	442	8	0.868621064	1.96	0.000903	0.0253	0.00395
Hydrocarbons	283	4	0.434310532	1.25	0.038	1	0.148
Hydrocarbon derivatives	14	1	0.108577633	0.0619	0.0602	1	0.211
Lignans, neolignans and related compounds	217	1	0.108577633	0.96	0.618	1	1
Lipids and lipid-like molecules	94100	268	29.09880565	416	1	1	1
Sum		921					
