# Supplementary material for: Integrated transcriptomic and metabolomic analyses reveal host–metabolite interactions underlying cloacal inflammation in laying Pekin ducks
Source: Anim Biosci. 2026 Apr 16;39(10):260208. doi: 10.5713/ab.260208 (PMC13662949; doi:10.5713/ab.260208)
Supplement: Supplementary file 3 [file ab-260208-Supplementary-12.pdf]

Supplement 12. Differential metabolites in cloacal contents of ducks with cloacal inflammation (ID) versus healthy controls (HC).

Group	N	Metabolites
Upregulated DEGs	152	metab_4737, Neosaxitoxin, metab_2957, metab_562, metab_2179, metab_382, metab_8395, metab_2529, metab_3835, metab_8276, metab_4565, metab_378, metab_4194, metab_2590, metab_2289, metab_7804, metab_4701, metab_8237, metab_4620, metab_8134, metab_2339, metab_8314, metab_262, metab_5429, metab_3818, metab_4440, metab_2237, metab_7514, metab_388, metab_2375, metab_4291, metab_8164, metab_5400, metab_43, metab_2373, metab_7825, metab_6005, metab_6543, metab_4532, metab_3559, metab_3772, metab_7874, metab_7823, metab_7790, metab_8606, metab_7819, metab_7880, metab_5099, metab_660, Aspartyl-Isoleucine, metab_848, metab_8285, metab_7833, metab_5935, metab_4617, Mevastatin, metab_7035, metab_8152, metab_8155, metab_7573, metab_7820, metab_3850, metab_1887, metab_840, Hydroxyphenylacetylglycine, metab_6560, metab_8511, metab_5552, metab_7826, metab_8151, metab_550, metab_7781, metab_559, metab_2506, metab_483, metab_8617, metab_4679, metab_6651, metab_4456, metab_7130, metab_7254, metab_8286, metab_7832, metab_1881, 2-Amino-4-methylsulfanylbutanal, metab_7799, metab_7785, metab_7824, metab_4718, PE(16:0/0:0), metab_8031, metab_6344, metab_8535, metab_6102, metab_7787, 7-(1-Imidazolyl)heptanoic acid, metab_8376, metab_7520, metab_8474, metab_7307, metab_3518, metab_5519, metab_8506, metab_5594, metab_5355, metab_7809, metab_7038, metab_5823, metab_5932, metab_4645, [3-[2-Aminoethoxy(hydroxy)phosphoryl]oxy-2-hydroxypropyl] hexadecanoate, metab_8488, metab_12, metab_1514, metab_7680, metab_4722, metab_2779, metab_8586, metab_5612, metab_7955, metab_6419, metab_5646, metab_7045, metab_5306, metab_6225, metab_4665, metab_6181, metab_5497, metab_7647, metab_8179, metab_6273, Cycloguanil, metab_5713, metab_4611, metab_7865, metab_7432, metab_7834, metab_6038, metab_6382, metab_1882, metab_6253, metab_6266, 1-Pentadecanoyl-sn-Glycero-3-Phosphocholine, metab_6272, metab_1545, metab_8383, metab_6474, metab_5747, metab_6242, metab_28, metab_7648, metab_5976
Downregulated DEGs	*106*	Thiazoline, metab_4057, metab_3369, metab_6464, metab_8153, metab_4753, metab_8365, metab_3676, metab_4634, MG(PGF2alpha/0:0/0:0), metab_2390, metab_1399, metab_7807, metab_6142, metab_6017, metab_6015, metab_2847, metab_6458, metab_1064, metab_7476, 1-(4-O-beta-D-glucopyranosyl-3-methoxyphenyl)-3,5-dihydroxydecane, metab_8616, metab_1117, metab_654, metab_232, metab_529, metab_3126, Sedoheptulose, metab_2777, metab_206, metab_4921, metab_3690, metab_6796, metab_7803, metab_4700, metab_3578, metab_8225, 2,5-Dihydroxybenzenesulfonic Acid, metab_8126, metab_2536, metab_8128, metab_3069, metab_3642, metab_4466, metab_1277, metab_6086, metab_3752, metab_4367, metab_665, metab_3525, metab_3513, metab_1351, Pyrocatechol, metab_2844, metab_3876, metab_1286, metab_8515, metab_7691, metab_3669, metab_8500, metab_2503, metab_6928, metab_3979, metab_3602, metab_8131, metab_1199, metab_836, metab_5318, metab_617, metab_2236, metab_3299, metab_107, metab_1478, metab_1435, metab_354, metab_2486, metab_393, metab_647, metab_4372, (E,E,E)-Geranylgeranyl alcohol, metab_2656, metab_2653, metab_203, metab_4775, metab_1550, 2'-C-Methylguanosine, metab_5103, metab_5412, metab_1952, 17-hydroxyprogesterone caproate, metab_151, LysoPC(18:3(6Z,9Z,12Z)/0:0), metab_2636, metab_2630, metab_1950, metab_2206, Isokobusone, metab_1475, Phytol, metab_5046, metab_700, metab_7924, metab_2845, Methylthiouracil, metab_5658, 4,5-seco-dopa
