# Supplementary material for: Integrated transcriptomic and metabolomic analyses reveal host–metabolite interactions underlying cloacal inflammation in laying Pekin ducks
Source: Anim Biosci. 2026 Apr 16;39(10):260208. doi: 10.5713/ab.260208 (PMC13662949; doi:10.5713/ab.260208)
Supplement: Supplementary file 4 [file ab-260208-Supplementary-13.pdf]

Supplement 13. Correlation analysis between transcriptomic DEGs and differential metabolites.

gene	metab	r	p
KCNG2	Thiazoline	-0.841176471	2.93E-05
LOC110351921	Thiazoline	0.811764706	0.000194873
PCBD1	Thiazoline	-0.811764706	0.000194873
C1QTNF8	PE(16:0/0:0)	0.803532226	0.000177058
PCBD1	(S)C(S)S-S-Methylcysteine sulfoxide	-0.802941176	0.00027427
KCNG2	(S)C(S)S-S-Methylcysteine sulfoxide	-0.794117647	0.000371456
LOC106015647	(S)C(S)S-S-Methylcysteine sulfoxide	0.788235294	0.000447446
LOC106015647	Thiazoline	0.785294118	0.000489119
LOC101798959	(9R,10S,12Z)-9,10-Dihydroxy-8-oxo-12-octadecenoic acid	-0.785294118	0.000489119
SH2B2	2-Amino-5-((1-((carboxymethyl)amino)-3-(methylthio)-1-oxopropan-2-yl)amino)-5-oxopentanoic acid	-0.782352941	0.000533395
CD180	Cycloguanil	0.767647059	0.000798026
FNDC1	Hydroxy Tyrosol -Acetate	-0.755882353	0.001069496
LOC106018497	(E,E,E)-Geranylgeranyl alcohol	-0.75	0.001228299
CRTAC1	17-hydroxyprogesterone caproate	0.75	0.001228299
LOC110351921	(S)C(S)S-S-Methylcysteine sulfoxide	0.747058824	0.001314011
LOC101800726	(9R,10S,12Z)-9,10-Dihydroxy-8-oxo-12-octadecenoic acid	-0.744117647	0.001404148
LOC119715705	Ethyl [3,4,5-trihydroxy-6-[2-[(4-methoxyphenyl)methyl]phenoxy]oxan-2-yl]methyl carbonate	-0.741176471	0.00149888
SH2B2	Glycine, L-g-glutamyl-L-cysteinyl-, 3-methyl ester	-0.735294118	0.00170283
LRRC15	(4S,8R)-8,9-Dihydroxy-p-menth-1(6)-en-2-one	-0.735294118	0.00170283
HSD17B2	Thiazoline	0.73142036	0.001282372
LOC106015647	Phenethylamine glucuronide	0.729411765	0.001927304
TECRL	Thiazoline	0.727427365	0.001405336
MYBL1	Isokobusone	0.726470588	0.00204771
LOC106017715	Thiazoline	0.724594857	0.001498259
LOC106017715	(S)C(S)S-S-Methylcysteine sulfoxide	0.724594857	0.001498259
LOC106017715	[3-[2-Aminoethoxy(hydroxy)phosphoryl]oxy-2-hydroxypropyl] hexadecanoate	-0.718454223	0.001716953
LOC101802072	Betavulgarin	0.714705882	0.002588434
LOC101800726	Chenodeoxycholyltaurine	-0.714705882	0.002588434
COL6A3	Phytol	-0.711764706	0.002739434
ADAMTS15	Phytol	-0.711764706	0.002739434
KDELR3	Hydroxy Tyrosol -Acetate	-0.711764706	0.002739434
C1QTNF8	Chenodeoxycholyltaurine	-0.710816969	0.002024469
COL6A3	(E,E,E)-Geranylgeranyl alcohol	-0.708823529	0.002897197
HSD17B2	(S)C(S)S-S-Methylcysteine sulfoxide	0.706401957	0.002221699
LOC119715705	Thiazoline	0.705882353	0.003061947
LOC119715005	(E,E,E)-Geranylgeranyl alcohol	-0.702941176	0.003233916
LOC119713249	Phenethylamine glucuronide	-0.702941176	0.003233916
C1QTNF8	Thiazoline	-0.701986945	0.002434226
LRRC15	Thiazoline	-0.7	0.00341334
LOC106019132	(4S,8R)-8,9-Dihydroxy-p-menth-1(6)-en-2-one	-0.698585278	0.002608954
KDELR3	Phytol	-0.697058824	0.003600458
ST6GALNAC5	Phytol	-0.691176471	0.00399877
LOC101802072	Risbitin	0.691176471	0.00399877
LOC106015023	Gaboxadol	-0.691176471	0.00399877
PPEF1	Phytol	-0.688235294	0.004210469
LOC106018497	Phytol	-0.688235294	0.004210469
LOC119715705	(S)C(S)S-S-Methylcysteine sulfoxide	0.688235294	0.004210469
LOC101803823	Phytol	-0.685294118	0.004430878
LOC101797788	O-Succinyl-L-homoserine	0.685294118	0.004430878
CYFIP2	Mactraxanthin	0.685294118	0.004430878
LOC101791564	PE(16:1(9Z)/P-18:1(11Z))	0.685294118	0.004430878
TMEM26	Hydroxy Tyrosol -Acetate	-0.685294118	0.004430878
COL3A1	Hydroxy Tyrosol -Acetate	-0.685294118	0.004430878
DRD5	5-Fluorodeoxyuridine monophosphate	-0.683658242	0.003499704
HSD17B2	PE(16:0/0:0)	-0.682855225	0.003553796
FKBP7	Phytol	-0.682352941	0.004660262
LOC101797788	Cycloguanil	0.682352941	0.004660262
LOC101802072	Chenodeoxycholyltaurine	-0.682352941	0.004660262
SPARC	Hydroxy Tyrosol -Acetate	-0.682352941	0.004660262
VCAN	Hydroxy Tyrosol -Acetate	-0.682352941	0.004660262
LOC119715705	Taurine	-0.682352941	0.004660262
TNC	Thiazoline	-0.679411765	0.004898893
CFAP46	Glucoheptonic acid	0.679411765	0.004898893
COL5A1	Hydroxy Tyrosol -Acetate	-0.679411765	0.004898893
KCNG2	PE(16:0/0:0)	0.676470588	0.005147047
LOC101791581	Neosaxitoxin	-0.676470588	0.005147047
LOC101801676	1-Phenyl-1-pentanone	-0.676470588	0.005147047
COL9A2	(S)C(S)S-S-Methylcysteine sulfoxide	-0.673529412	0.005405006
LOC113843513	2-Amino-5-((1-((carboxymethyl)amino)-3-(methylthio)-1-oxopropan-2-yl)amino)-5-oxopentanoic acid	-0.673529412	0.005405006
COL6A3	Hydroxy Tyrosol -Acetate	-0.673529412	0.005405006
ADAMTS15	Hydroxy Tyrosol -Acetate	-0.673529412	0.005405006
COL1A2	(E,E,E)-Geranylgeranyl alcohol	-0.670588235	0.005673057
LOC101805457	Isokobusone	0.670588235	0.005673057
LOC101795539	Cuminaldehyde	-0.670588235	0.005673057
CARMIL2	Ethyl [3,4,5-trihydroxy-6-[2-[(4-methoxyphenyl)methyl]phenoxy]oxan-2-yl]methyl carbonate	0.670588235	0.005673057
LOC119714651	(9R,10S,12Z)-9,10-Dihydroxy-8-oxo-12-octadecenoic acid	-0.670588235	0.005673057
LOC119714651	1-Phenyl-1-pentanone	-0.670588235	0.005673057
LOC119718509	PE(16:1(9Z)/P-18:1(11Z))	0.670588235	0.005673057
LOC113840033	N-(N-L-gamma-Glutamyl-L-cysteinyl)glycine monoethyl ester	0.670588235	0.005673057
RCN1	(E,E,E)-Geranylgeranyl alcohol	-0.667647059	0.005951492
COL1A1	(E,E,E)-Geranylgeranyl alcohol	-0.667647059	0.005951492
TLR1-A	Phenethylamine glucuronide	-0.667647059	0.005951492
KLHL6	PE(16:1(9Z)/P-18:1(11Z))	0.667647059	0.005951492
COL11A1	(4S,8R)-8,9-Dihydroxy-p-menth-1(6)-en-2-one	-0.667647059	0.005951492
CCN4	Hydroxy Tyrosol -Acetate	-0.667647059	0.005951492
SPARC	(E,E,E)-Geranylgeranyl alcohol	-0.664705882	0.006240608
CCN4	Cuminaldehyde	-0.664705882	0.006240608

LOC119718408	Phenethylamine glucuronide	0.664705882	0.006240608
COL1A2	Hydroxy Tyrosol -Acetate	-0.664705882	0.006240608
ADAMTS2	Hydroxy Tyrosol -Acetate	-0.664705882	0.006240608
LOC101800424	1-Pentadecanoyl-sn-Glycero-3-Phosphocholine	0.662760392	0.005142331
LOC106018497	Isokobusone	-0.661764706	0.006540708
COL9A2	Thiazoline	-0.661764706	0.006540708
LOC101802072	Apigenin 7-arabinoside	0.661764706	0.006540708
LOC106015023	LysoPC(0:0/18:2(9Z,12Z))	0.661764706	0.006540708
CD22	Cycloguanil	0.661764706	0.006540708
CD180	12-Hydroxyhexadecanoylcarnitine	-0.661764706	0.006540708
LOC119715705	Phenylethylmalonamide	-0.661764706	0.006540708
LOC106015647	Gibberellin A9	0.661764706	0.006540708
LOC101805457	Ser-Tyr	0.661764706	0.006540708
LOC101793878	Difloxacin	-0.661764706	0.006540708
GPX7	Hydroxy Tyrosol -Acetate	-0.661764706	0.006540708
MINDY4B	N-(N-L-gamma-Glutamyl-L-cysteinyl)glycine monoethyl ester	-0.660780164	0.005325426
CKAP4	PE(16:0/0:0)	0.658823529	0.0068521
PPEF1	MG(PGF2alpha/0:0/0:0)	-0.658823529	0.0068521
C1QTNF8	[3-[2-Aminoethoxy(hydroxy)phosphoryl]oxy-2-hydroxypropyl] hexadecanoate	0.657836823	0.005607166
HSD17B2	(4S,8R)-8,9-Dihydroxy-p-menth-1(6)-en-2-one	0.657836823	0.005607166
COL9A2	(E,E,E)-Geranylgeranyl alcohol	-0.655882353	0.007175095
COL6A3	Isokobusone	-0.655882353	0.007175095
LOC119714651	Chenodeoxycholyltaurine	-0.655882353	0.007175095
LOC101798959	8-(5-Pentylfuran-2-yl)octanoylcarnitine	-0.655882353	0.007175095
LOC101805457	Hydroxy Tyrosol -Acetate	0.655882353	0.007175095
LOC119713095	Thiazoline	-0.65342181	0.006051973
IGSF10	(E,E,E)-Geranylgeranyl alcohol	-0.652941176	0.007510013
FNDC1	Isokobusone	-0.652941176	0.007510013
FKBP7	Isokobusone	-0.652941176	0.007510013
GPX7	Phytol	-0.652941176	0.007510013
LOC101791390	Hydroxy Tyrosol -Acetate	0.652941176	0.007510013
PDZK1IP1	PE(16:0/0:0)	-0.65	0.007857177
LOC101804042	Thiazoline	0.65	0.007857177
LOC106015023	17-hydroxyprogesterone caproate	0.65	0.007857177
COL1A2	Phytol	-0.65	0.007857177
LOC101804042	Imipramine	-0.65	0.007857177
GMPR	2-Amino-5-((1-((carboxymethyl)amino)-3-(methylthio)-1-oxopropan-2-yl)amino)-5-oxopentanoic acid	-0.65	0.007857177
TNFRSF13B	Ser-Tyr	0.65	0.007857177
ABHD8	Hydroxy Tyrosol -Acetate	-0.65	0.007857177
LOC101797788	Taurine	0.65	0.007857177
HSD17B2	Betavulgarin	-0.649006798	0.006524479
TECRL	(S)C(S)S-S-Methylcysteine sulfoxide	0.648906692	0.006535523
KDELR3	Isokobusone	-0.647058824	0.008216914
SPARC	MG(PGF2alpha/0:0/0:0)	-0.647058824	0.008216914
P4HA3	8-(5-Pentylfuran-2-yl)octanoylcarnitine	-0.647058824	0.008216914
LOC106017715	PE(16:0/0:0)	-0.64476661	0.007005483
LOC119718408	Thiazoline	0.644117647	0.008589558
LOC101791564	3,5,7,8-Tetrahydro-2-[4-(trifluoromethyl)phenyl]-4H-thiopyrano[4,3-d]pyrimidin-4-one	0.644117647	0.008589558
LOC119715065	Ethyl [3,4,5-trihydroxy-6-[2-[(4-methoxyphenyl)methyl]phenoxy]oxan-2-yl]methyl carbonate	0.644117647	0.008589558
LOC119713784	Phenethylamine glucuronide	-0.644117647	0.008589558
SASH3	Mactraxanthin	0.644117647	0.008589558
ADAM12	Hydroxy Tyrosol -Acetate	-0.644117647	0.008589558
COL6A1	Hydroxy Tyrosol -Acetate	-0.644117647	0.008589558
COL1A1	Hydroxy Tyrosol -Acetate	-0.644117647	0.008589558
LOC106017715	1-Palmitoylphosphatidylcholine	-0.641696293	0.007371054
C1QTNF8	(S)C(S)S-S-Methylcysteine sulfoxide	-0.641648444	0.007376868
SERPINH1	(E,E,E)-Geranylgeranyl alcohol	-0.641176471	0.008975447
CTHRC1	(E,E,E)-Geranylgeranyl alcohol	-0.641176471	0.008975447
ADAMTS15	(E,E,E)-Geranylgeranyl alcohol	-0.641176471	0.008975447
LOC119714651	2-Amino-4-methylsulfanylbutanal	0.641176471	0.008975447
GPR18	3,5,7,8-Tetrahydro-2-[4-(trifluoromethyl)phenyl]-4H-thiopyrano[4,3-d]pyrimidin-4-one	0.641176471	0.008975447
LOC101791390	Ser-Tyr	0.641176471	0.008975447
KCNG2	Asi-222	0.641176471	0.008975447
RCN1	Hydroxy Tyrosol -Acetate	-0.641176471	0.008975447
LOC106017715	Tyrosyl-Lysine	-0.638625976	0.007751617
P4HA3	PE(16:0/0:0)	0.638235294	0.009374926
LOC101802072	Thiazoline	-0.638235294	0.009374926
LOC101793957	Thiazoline	-0.638235294	0.009374926
CKAP4	Phytol	-0.638235294	0.009374926
RCN1	Phytol	-0.638235294	0.009374926
CARD9	Phenethylamine glucuronide	-0.638235294	0.009374926
LOC101800726	1-Phenyl-1-pentanone	-0.638235294	0.009374926
PPEF1	Difloxacin	-0.638235294	0.009374926
LOC110352957	(4S,8R)-8,9-Dihydroxy-p-menth-1(6)-en-2-one	0.638235294	0.009374926
LOC101792124	Hydroxy Tyrosol -Acetate	-0.638235294	0.009374926
SGIP1	Hydroxy Tyrosol -Acetate	-0.638235294	0.009374926
ADAMTS15	Methylprednisolone acetate	0.638235294	0.009374926
ADAM12	(E,E,E)-Geranylgeranyl alcohol	-0.635294118	0.009788343
LOC101797788	Ethyl [3,4,5-trihydroxy-6-[2-[(4-methoxyphenyl)methyl]phenoxy]oxan-2-yl]methyl carbonate	0.635294118	0.009788343
LOC119718408	(S)C(S)S-S-Methylcysteine sulfoxide	0.635294118	0.009788343
LOC106017715	Cycloguanil	-0.632485342	0.00855946
LOC101797052	(E,E,E)-Geranylgeranyl alcohol	-0.632352941	0.010216051
ADAMTS15	Isokobusone	-0.632352941	0.010216051
CARD9	Thiazoline	-0.632352941	0.010216051
LOC119715705	Betavulgarin	-0.632352941	0.010216051
LOC101791564	2,5-Dihydroxybenzenesulfonic Acid	0.632352941	0.010216051
SPARC	1-(4-O-beta-D-glucopyranosyl-3-methoxyphenyl)-3,5-dihydroxydecane	-0.632352941	0.010216051
LUM	Phytol	-0.632352941	0.010216051
TMEM26	Phytol	-0.632352941	0.010216051
KLHL34	5-Fluorodeoxyuridine monophosphate	-0.632352941	0.010216051
LOC101793957	(S)C(S)S-S-Methylcysteine sulfoxide	-0.632352941	0.010216051
LOC101797788	Tyrosyl-Lysine	0.632352941	0.010216051
COL5A2	Hydroxy Tyrosol -Acetate	-0.632352941	0.010216051

SH2B2	Leucodopachrome	-0.632352941	0.010216051
LOC119713095	(S)C(S)S-S-Methylcysteine sulfoxide	-0.631346749	0.008716314
COL6A3	PE(16:0/0:0)	0.629411765	0.010658409
TNC	Betavulgarin	0.629411765	0.010658409
CRTAC1	LysoPC(0:0/18:2(9Z,12Z))	0.629411765	0.010658409
SASH3	2-Amino-5-((1-((carboxymethyl)amino)-3-(methylthio)-1-oxopropan-2-yl)amino)-5-oxopentanoic acid	-0.629411765	0.010658409
IL1RL1	Phenethylamine glucuronide	-0.629411765	0.010658409
LOC110351193	Phenethylamine glucuronide	-0.629411765	0.010658409
PPEF1	(E,E,E)-Geranylgeranyl alcohol	-0.626470588	0.01111578
ST6GALNAC5	Isokobusone	-0.626470588	0.01111578
TNC	(S)C(S)S-S-Methylcysteine sulfoxide	-0.626470588	0.01111578
LOC119715705	O-Succinyl-L-homoserine	-0.626470588	0.01111578
LOC101793940	Mactraxanthin	0.626470588	0.01111578
LOC101793940	PE(16:1(9Z)/P-18:1(11Z))	0.626470588	0.01111578
PPEF1	Hydroxy Tyrosol -Acetate	-0.626470588	0.01111578
ELN	Hydroxy Tyrosol -Acetate	-0.626470588	0.01111578
LUM	Isokobusone	-0.623529412	0.011588534
LOC119713249	Thiazoline	-0.623529412	0.011588534
SPARC	Phytol	-0.623529412	0.011588534
CD22	12-Hydroxyhexadecanoylcarnitine	-0.623529412	0.011588534
MZB1	Ethyl [3,4,5-trihydroxy-6-[2-[(4-methoxyphenyl)methyl]phenoxy]oxan-2-yl]methyl carbonate	0.623529412	0.011588534
LOC119715065	O-Succinyl-L-homoserine	0.623529412	0.011588534
ARHGAP9	2-Amino-5-((1-((carboxymethyl)amino)-3-(methylthio)-1-oxopropan-2-yl)amino)-5-oxopentanoic acid	-0.623529412	0.011588534
C1QC	Phenethylamine glucuronide	-0.623529412	0.011588534
LOC101803823	(4S,8R)-8,9-Dihydroxy-p-menth-1(6)-en-2-one	-0.623529412	0.011588534
KCNG2	Methylprednisolone acetate	0.623529412	0.011588534
LOC113845763	Isokobusone	0.620588235	0.012077043
LOC101791902	17-hydroxyprogesterone caproate	-0.620588235	0.012077043
FNDC1	Phytol	-0.620588235	0.012077043
IRAG2	Cycloguanil	0.620588235	0.012077043
KLHL34	Cycloguanil	-0.620588235	0.012077043
COL1A1	Cuminaldehyde	-0.620588235	0.012077043
LOC119718408	Difloxacin	0.620588235	0.012077043
LOC101791581	Difloxacin	0.620588235	0.012077043
LOC106015023	N-(N-L-gamma-Glutamyl-L-cysteinyl)glycine monoethyl ester	-0.620588235	0.012077043
LUM	Hydroxy Tyrosol -Acetate	-0.620588235	0.012077043
SERPINH1	Hydroxy Tyrosol -Acetate	-0.620588235	0.012077043
ST6GALNAC5	5-Acetylamino-6-formylamino-3-methyluracil	0.620588235	0.012077043
TECRL	[3-[2-Aminoethoxy(hydroxy)phosphoryl]oxy-2-hydroxypropyl] hexadecanoate	-0.617794727	0.010763761
SH2B2	17-hydroxyprogesterone caproate	0.617647059	0.012581685
LOC101791581	Apigenin 7-arabinoside	-0.617647059	0.012581685
LOC101797052	Phytol	-0.617647059	0.012581685
DNAH1	Cuminaldehyde	-0.617647059	0.012581685
LOC119718310	(S)C(S)S-S-Methylcysteine sulfoxide	0.617647059	0.012581685
CARD9	(S)C(S)S-S-Methylcysteine sulfoxide	-0.617647059	0.012581685
IL21R	PE(16:1(9Z)/P-18:1(11Z))	0.617647059	0.012581685
RUNX3	PE(16:1(9Z)/P-18:1(11Z))	0.617647059	0.012581685
LOC101791581	Mepenzolate	-0.617647059	0.012581685
CKAP4	(E,E,E)-Geranylgeranyl alcohol	-0.614705882	0.013102844
TMEM26	(E,E,E)-Geranylgeranyl alcohol	-0.614705882	0.013102844
CKAP4	Isokobusone	-0.614705882	0.013102844
LOC101792124	Phytol	-0.614705882	0.013102844
FKBP10	(S)C(S)S-S-Methylcysteine sulfoxide	-0.614705882	0.013102844
PCBD1	Phenethylamine glucuronide	-0.614705882	0.013102844
WDFY4	Phenethylamine glucuronide	-0.614705882	0.013102844
KLHL6	Mactraxanthin	0.614705882	0.013102844
LOC101791564	Mactraxanthin	0.614705882	0.013102844
ST6GALNAC5	Chenodeoxycholyltaurine	-0.614705882	0.013102844
CYFIP2	PE(16:1(9Z)/P-18:1(11Z))	0.614705882	0.013102844
LOC101805457	PE(16:1(9Z)/P-18:1(11Z))	0.614705882	0.013102844
COL6A3	Methylprednisolone acetate	0.614705882	0.013102844
HSD17B2	[3-[2-Aminoethoxy(hydroxy)phosphoryl]oxy-2-hydroxypropyl] hexadecanoate	-0.6136867	0.011453833
PTX3	LysoPC(18:3(6Z,9Z,12Z)/0:0)	-0.611764706	0.013640906
TMEM26	Isokobusone	-0.611764706	0.013640906
LOC110351193	Thiazoline	-0.611764706	0.013640906
CFAP46	Phytol	-0.611764706	0.013640906
P4HA3	Cuminaldehyde	-0.611764706	0.013640906
RCN1	Cuminaldehyde	-0.611764706	0.013640906
SPARC	Cuminaldehyde	-0.611764706	0.013640906
MZB1	Phenylethylmalonamide	0.611764706	0.013640906
CCL26	Ser-Tyr	0.611764706	0.013640906
GHRH	Gaboxadol	0.611764706	0.013640906
LOC101800726	8-(5-Pentylfuran-2-yl)octanoylcarnitine	-0.611764706	0.013640906
LOC106017715	Cholylglutamine	-0.610993121	0.011924858
LOC101792124	(E,E,E)-Geranylgeranyl alcohol	-0.608823529	0.014196265
LOC101804042	[3-[2-Aminoethoxy(hydroxy)phosphoryl]oxy-2-hydroxypropyl] hexadecanoate	-0.608823529	0.014196265
PTX3	MG(PGF2alpha/0:0/0:0)	-0.608823529	0.014196265
TMEM45A	Phytol	-0.608823529	0.014196265
VCAN	Phytol	-0.608823529	0.014196265
MZB1	Cycloguanil	0.608823529	0.014196265
ADAM12	Cuminaldehyde	-0.608823529	0.014196265
LOC101804042	Phenylethylmalonamide	-0.608823529	0.014196265
LPXN	Phenethylamine glucuronide	-0.608823529	0.014196265
LOC101798959	Difloxacin	-0.608823529	0.014196265
LOC119714651	Gaboxadol	0.608823529	0.014196265
WDFY4	PE(16:1(9Z)/P-18:1(11Z))	0.608823529	0.014196265
ST6GALNAC5	Asi-222	0.608823529	0.014196265
ADAMTS15	Asi-222	0.608823529	0.014196265
LOC101795539	8-(5-Pentylfuran-2-yl)octanoylcarnitine	-0.608823529	0.014196265
CDH6	N-(N-L-gamma-Glutamyl-L-cysteinyl)glycine monoethyl ester	-0.608514221	0.01237166
MINDY4B	2-Amino-5-((1-((carboxymethyl)amino)-3-(methylthio)-1-oxopropan-2-yl)amino)-5-oxopentanoic acid	-0.606328347	0.012776447
COL11A1	(E,E,E)-Geranylgeranyl alcohol	-0.605882353	0.014769317
ADAMTS15	PE(16:0/0:0)	0.605882353	0.014769317

LOC101802072	7-(1-Imidazolyl)heptanoic acid	0.605882353	0.014769317
CST7	3,5,7,8-Tetrahydro-2-[4-(trifluoromethyl)phenyl]-4H-thiopyrano[4,3-d]pyrimidin-4-one	0.605882353	0.014769317
ADAMTS15	Cuminaldehyde	-0.605882353	0.014769317
POU2AF1	Ethyl [3,4,5-trihydroxy-6-[2-[(4-methoxyphenyl)methyl]phenoxy]oxan-2-yl]methyl carbonate	0.605882353	0.014769317
FBLIM1	(9R,10S,12Z)-9,10-Dihydroxy-8-oxo-12-octadecenoic acid	-0.605882353	0.014769317
LOC101794304	2-Amino-5-((1-((carboxymethyl)amino)-3-(methylthio)-1-oxopropan-2-yl)amino)-5-oxopentanoic acid	-0.605882353	0.014769317
LPXN	2-Amino-5-((1-((carboxymethyl)amino)-3-(methylthio)-1-oxopropan-2-yl)amino)-5-oxopentanoic acid	-0.605882353	0.014769317
GCM1	Oleyl alcohol	-0.605882353	0.014769317
P4HA3	Chenodeoxycholyltaurine	-0.605882353	0.014769317
LOC101798959	Chenodeoxycholyltaurine	-0.605882353	0.014769317
LOC113843595	Hydroxy Tyrosol -Acetate	-0.605882353	0.014769317
HSD17B2	Phenethylamine glucuronide	0.604856676	0.013054773
ABHD8	(E,E,E)-Geranylgeranyl alcohol	-0.602941176	0.015360462
LOC101803823	Sedoheptulose	-0.602941176	0.015360462
PPEF1	Methylthiouracil	-0.602941176	0.015360462
PPEF1	Cuminaldehyde	-0.602941176	0.015360462
LOC101795539	12-Hydroxyhexadecanoylcarnitine	-0.602941176	0.015360462
LOC119718408	12-Hydroxyhexadecanoylcarnitine	0.602941176	0.015360462
LOC101793676	(9R,10S,12Z)-9,10-Dihydroxy-8-oxo-12-octadecenoic acid	-0.602941176	0.015360462
CARMIL2	O-Succinyl-L-homoserine	0.602941176	0.015360462
LOC101793957	Phenethylamine glucuronide	-0.602941176	0.015360462
LOC101791564	Genistein	0.602941176	0.015360462
MYBL1	Mactraxanthin	0.602941176	0.015360462
CST7	PE(16:1(9Z)/P-18:1(11Z))	0.602941176	0.015360462
FBLIM1	(E,E,E)-Geranylgeranyl alcohol	-0.6	0.015970108
FKBP10	Thiazoline	-0.6	0.015970108
KCNG2	Betavulgarin	0.6	0.015970108
COL9A2	Apigenin 7-arabinoside	0.6	0.015970108
FKBP10	Apigenin 7-arabinoside	0.6	0.015970108
PPEF1	1-(4-O-beta-D-glucopyranosyl-3-methoxyphenyl)-3,5-dihydroxydecane	-0.6	0.015970108
PTX3	1-(4-O-beta-D-glucopyranosyl-3-methoxyphenyl)-3,5-dihydroxydecane	-0.6	0.015970108
IGSF10	Phytol	-0.6	0.015970108
LOC101798959	Cuminaldehyde	-0.6	0.015970108
LOC119713249	(S)C(S)S-S-Methylcysteine sulfoxide	-0.6	0.015970108
LOC101791581	Imipramine	-0.6	0.015970108
TRAF3IP3	Phenethylamine glucuronide	-0.6	0.015970108
LOC119715065	Phenethylamine glucuronide	-0.6	0.015970108
RUNX3	Genistein	0.6	0.015970108
SASH3	PE(16:1(9Z)/P-18:1(11Z))	0.6	0.015970108
CNMD	(4S,8R)-8,9-Dihydroxy-p-menth-1(6)-en-2-one	0.6	0.015970108
