# Supplementary material for: Integrated transcriptomic and metabolomic analyses reveal host–metabolite interactions underlying cloacal inflammation in laying Pekin ducks
Source: Anim Biosci. 2026 Apr 16;39(10):260208. doi: 10.5713/ab.260208 (PMC13662949; doi:10.5713/ab.260208)
Supplement: Supplementary file 5 [file ab-260208-Supplementary-2.pdf]

Supplement 2. Significantly differentially expressed genes in ID vs NID comparison.

Group	N	Genes
Upregulated DEGs	451	LOC119713823, CDH6, ESR2, SIX3, DRD5, MDGA1, LOC106017715, DUSP29, OSTN, TECRL, LOC119713219, LOC113840355, LOC110352957, C7H21orf58, LOC119713220, LOC110351497, LOC113842379, ASB18, TMEM233, LOC101801612, LOC101803316, ASB15, STRIT1, ARX, LOC101791401, LOC101794508, LOC101796474, LOC119713167, LOC101802165, LOC101802071, LOC113844155, MYOM2, GPD1, LOC101794221, ASB4, MAFA, LOC119715992, LOC119713221, GRIN2B, LOC101793478, LOC101790909, PPDPFL, RPL3L, LOC110353953, MYBPH, LOC106016943, LOC119712999, NWD1, DRGX, OBSL1, C2H8orf34, LMOD3, MYLK2, IP6K3, ACTA1, LOC119715271, MYL1, LOC106018919, LOC101801311, LOC106014873, LOC119717776, NT5C1A, MYH7B, LOC106015356, NR4A1, LOC113842688, SMTNL2, LOC119718360, ANKRD2, LOC106017289, C6H10orf71, SLC8A3, CRTAC1, CLCN2, SLC30A2, LOC106015797, USP2, LOC110351399, KCNH7, ASB10, LRRC2, DSCAM, TNNT3, KLHL40, IRX1, TEKT5, LOC119714566, OBSCN, PDK4, LOC113842930, MYLK3, LOC110353597, HSD17B2, IRX2, LOC101791922, LOC119718254, PNMT, LOC119713229, EEF1A2, ERBB4, PLN, ARMC12, LOC101804042, FSD2, LOC101795019, LDB3, KCNK9, CNMD, LOC101795309, USP13, SNCB, CORO6, LOC113840724, LOC113843713, LOC101793493, JPH1, STAC3, PKD2L2, SLN, KLHL30, FHL2, LOC113840410, LOC106019710, LOC101800711, CLDN14, SYNPO2L, FHL3, TRDN, GRIK4, PDE4C, TMEM255B, LOC106015458, LOC106017354, IRS2, NEB, TEKT1, PPP1R1A, LDHD, TTN, LOC119716681, CRYAB, MYO18B, SGCZ, SLC25A4, LOC119715705, LOC119714529, FHL1, RAMP1, LOC101793529, ALPK3, FKBP1B, SMPX, LOC101794749, LOC119714325, LOC119715738, ATP2A2, LOC119716429, LOC119715431, LOC113840188, HSPB7, LOC119716546, LOC110351921, RTN4R, SGCA, LOC110352115, TMEM74, LOC113842743, LOC106016406, MYLK4, BEST3, LOC113845049, LOC119716592, LOC101798971, LOC101799407, TXLNB, CMYA5, LOC119714295, CLSTN2, LOC113841418, TRIM54, LOC101795226, ANO8, LOC101800783, LRRC56, KLHL34, CHRNA1, MSTN, LOC101804425, MYL2, CPT1B, TMEM38A, LOC119718029, FGF18, PRKAB2, LOC119718310, LOC106018705, LOC119717819, RAPGEF4, ACACB, CLCN1, FSD1L, SEMA6B, EYA4, REEP6, LOC119718304, RNF207, FRMD3, LOC113844214, TMOD1, FGF13, LOC119717734, ARPP21, DUSP26, LOC101794131, HSPB1, TPM3, RBM24, IGDCC4, CAMK2A, LOC101790517, LRRC14B, BCAN, LOC113843972, TMEM229B, PCBP4, ADAMTS20, LOC113843890, LRRC20, LOC119717600, KLHL14, RIMBP2, LIMCH1, RXFP2, ANO4, COLQ, SPEG, ASB11, CAP2, LOC113841470, C5H14orf180, LOC106019894, SLC7A2, STRIP2, ANKEF1, LOC101789676, ALPK2, TMEM132D, APOH, LOC119715432, LOC119716801, ABLIM2, CUTA, LOC106016021, TPM2, PGM1, PDLIM5, NRN1L, LAPTM4B, LOC110353809, PABPC4, CAPN3, SIK2, EPDR1, KBTBD13, COQ8A, LSMEM1, IGSF21, LOC110354152, LOC113844903, LOC101797856, HELT, LOC101802901, S100A1, LOC119718301, LOC119715213, DKK2, TMEM52, RANBP3L, STYXL2, LRRC38, LOC101793418, DNAJB13, MLF1, LOC101794949, KCNH2, FKBP3, LOC119715435, LOC119713150, GMPR, LOC101792067, ADCY2, SLC38A3, RYR3, TMOD4, PHKA1, FZD9, NEXN, LOC110351385, LOC101804257, RGS11, POPDC2, KCNJ2, ADSS1, RAPGEF5, PCP4, LOC119718305, KCND2, LOC101802516, SH3BP5, LOC110352082, LOC101794644, SPTB, LOC119718300, LOC106015023, LRRTM4, SLC25A34, LOC113845281, NR4A3, NSUN7, PDZK1IP1, TEAD4, LOC101804932, HS3ST2, CNTNAP5, NMNAT3, STC2, BOLL, GAMT, LOC119718096, NDRG4, BIN1, LOC119717257, PLP1, LOC119714067, RALY, COQ10A, BCKDHB, FBXO15, LOC113843748, CEP85, PLPPR1, MINAR1, PPDPF, GCM1, LOC119717767, LOC119718309, AJM1, REEP1, LOC101791673, MAPK8IP1, ACVR1C, FAM162B, PKIA, KCNT1, PRSS12, RASGEF1B, LOC106018273, LOC119714223, WFIKKN1, SNX22, DES, SESN2, SSUH2, HSPB8, SLC6A8, LOC101801369, MCRIP2, ACYP2, FILIP1, PLCXD2, LOC101796791, ZNF106, GPAM, SCN2B, ZBTB16, CLTCL1, TM6SF1, PIWIL1, LOC113843555, PM20D2, LOC119714192, LOC113845031, MCF2, TBX18, unassigned_gene_1, WIPF3, LOC119718408, PEBP4, LOC106015647, RETREG1, PRKAG3, LOC101802576, PLCD4, LOC101802038, EPM2A, PDK2, GPC3, unassigned_gene_11, GPM6B, SIK1, PDE4B, PKNOX2, KHDRBS3, IRS1, LOC101791581, unassigned_gene_12, LOC119716632, JPH2, SLC25A12, LOC113844749, LOC106015025, LOC101793413, FEM1C, SLC27A1, ISM1, FKBP5, LOC106016919, NFATC1, PLCL1, ACO2, LOC101791736, SLC16A7, LOC106014639, ADHFE1, LOC119717011, CD320, TPPP3, ELAVL4, ACADL, NSMF, SGCD, LRRC39, TMEM82, MTCL1, AQP4, CISD3, SLC25A33, SYNM, TMEM109, RBM38, AGBL3, CACNA2D1, PLPP7, SCYL3, SPRY2, LOC119718147, CFL2, SH2B2, FXR1, PPM1L, PNPLA2
Downregulated DEGs	436	LOC106019132, LOC101798959, LOC101793057, LOC110354560, LOC110351995, LOC119713829, LOC101802909, B4GALT6, LOC101799898, LOC101803727, LOC101803082, LOC101791569, IL8, C8B, BMP3, LOC119714395, SYT8, LOC101796665, COL11A1, SAA2, LOC119714069, LOC101801508, LOC106014594, LOC101802211, SPP1, LOC110351193, LOC101800424, LOC101801676, LOC119717029, CTHRC1, LOC101794528, ZP1, LOC101793676, LOC113840231, LOC119717042, LOC119717040, TNC, PRLH, LOC101793441, CHRNA4, WNT16, MINDY4B, C1QTNF8, LOC119713475, SERTM2, LOC113843959, COL6A6, LOC119717036, TLR7, LOC119718349, LOC101804519, PTGS2, LOC101793957, PDCD1, TMEM45A, LOC101795539, CCL26, LOC101797788, NKX6-2, LOC119713916, LOC101791385, LOC106019940, INHBA, CD22, SLC2A6, LOC101793778, LOC119715005, EVX1, LOC113842721, LOC101790301, LOC101793878, PIRT, LOC119717888, LOC101803817, PRAM1, LOC119714144, DCHS2, LOC101802072, TMEM26, DRD4, LOC101797500, LOC106017965, LOC119718509, LOC101794304, MLKL, ADAMTS18, TNFRSF13B, LOC113845350, ESM1, LOC106015430, LOC101800358, LOC101791674, SRMS, HPGDS, LOC119717041, ACOD1, MZB1, LOC119713140, COL1A1, LOC101791902, FAM163A, ASS1, BRSK2, LOC119717944, MPEG1, LOC119713095, LOC101801783, LOC101798845, DCX, C3, ZNF831, TLR1-A, GP1BA, LOC101803823, COL3A1, GAP43, PAX6, ADAM8, CENPA, LOC101789851, LOC119714296, LOC113841492, GHRH, PTX3, ADAMTSL2, LOC113845274, LOC119712955, LOC101795569, LOC110353216, LOC101801606, GPR18, LOC101798264, LOC119715439, COL1A2, CCR10, HAS3, LOC101796765, FKBP11, ADAM12, LOC119713036, CST7, LOC119714156, ATP4A, LOC113841770, ADAMTS12, LOC101802760, DTX1, TNFSF8, MMP9, LOC101793260, LOC101805457, CFAP251, GPR132, MARCO, LOC101794299, LOC101800757, DDC, LOC106018497, CD247, CCL22, LOC101801498, GNG2, UBE2C, COL5A1, LOC119718666, EOMES, CA10, KDELR3, SPARC, DNAJC5B, PCBD1, LOC101797052, LOC106019440, LOC119714803, ELN, CKS1B, ADAMTS2, LOC119718710, CXCR4, CD3E, IL21R, GPX7, LOC119714651, LOC119717889, LOC119713033, LOC113840033, LOC101797666, CFAP46, LOC101804783, CARD9, CRYBB1, RCN1, LOC113842387, LOC119718662, FSCN1, LOC119713971, BATF, DBH, LOC113839921, LOC106020629, LOC106020111, POU2AF1, LOC101797566, TASL, MSX1, COL26A1, LOC101802873, LOC101803429, CNRIP1, ABHD8, KCNG2, CDCA3, LOC101800311, SHMT2, LOC101798133, LOC101803633, ADAMTS15, LOC101792171, ITGB2, TRIM71, LCP1, LOC119715859, PLK1, CASR, RHOH, TSPEAR, FBN3, LOC119713784, CREB3L1, LY96, LOC119713918, SRGN, LOC119715065, ITK, LOC106017547, PRR11, TAFA3, LOC101803950, TM4SF19, LOC101800429, SLA, SPRY4, LOC101802067, LOC110351762, C1QC, LPXN, IL2RG, FKBP10, MFNG, ADGRG7, VCAM1, LOC101798037, WDFY4, IGSF10, LOC119717051, LOC101803193, IKZF1, IL6, CDH3, LOC101793812, IRF4, GPR65, IL1RL1, LOC119718395, CDCA8, LRRC23, HHIPL1, EVI2A, C3AR1, IGSF6, QPCT, SAMSN1, COL5A2, LOC101795053, CARMIL2, IL18R1, STEAP1, P4HA3, LOC113845409, THSD7A, ABCG4, TLR-15, LOC101791564, CNN2, LOC113840340, LOC101800880, ADAMTS10, SERPINH1, TNFSF11, NOS2, COL6A3, SNAI1, KIF21B, RHEX, RHOG, ALOX5, LOC113843595, LOC101803964, NCF2, EVI2B, PIK3R5, STC1, PTPRC, RGS9, LOC119718678, LOC101797774, LOC119718589, TRAF3IP3, RRM2, KLHL6, LOC119715357, ARHGAP15, IFI30, LOC101791390, LOC113840683, TK1, WNT1, LOC113842636, LOC113843744, ANKRD34A, LOC101796407, LOC101793233, FNDC1, LOC101802747, WSCD1, FBLIM1, LOC101803870, LRRC15, MYBL1, GPRIN3, C1QB, LOC119713249, PPEF1, OMG, ESPL1, LEF1, VCAN, GPER1, PTCHD3, TDRD5, LOC101789766, DNAH1, LOC101800726, LOC113843513, CCL19, LOC101804340, CD180, LOC119714196, RUNX3, IRAG2, KPNA2, COL9A2, ARHGAP9, CARD11, LOC119713956, LOC101797250, LOC101793086, ST8SIA1, TNIP3, LOC119716063, PEAK3, DOCK2, C1QTNF6, CENPO, LOC101791991, LOC119714135, CCN4, DOK2, CKAP4, LOC101795552, FN1, LOC119717472, LOC101799520, GJC2, PIF1, C1QA, TUBB3, SH3TC2, TRARG1, SGIP1, TTLL10, ADGRG5, CX3CR1, LOC113845767, PIK3CD, CYFIP2, ABI3, FBN2, LOC101791359, FKBP7, LOC101792402, SASH3, DNASE2B, LCK, E2F1, CEP55, LOC101803634, TLR4, DEPDC1B, CCNB3, LOC119716678, LOC101792606, LOC101792124, LOC119714503, LAMP3, CD40LG, CADM3, KIF14, NDC80, PRSS35, LOC101793940, RGS19, PTTG1, LOC110351185, LOC106019771, DEPDC1, LOC119713016, LOC106014292, P2RY13, LOC101805472, LOC101804178, ST6GALNAC5, C6H10orf95, PXYLP1, LIMD2, SKA1, LOC113845763, COL6A1, TMEM71, LOC106018390, LAMP5, KIF18B, XYLT1, LUM, LOC119713786, LOC119713674

Supplement 3. Significantly differentially expressed genes in ID vs HC comparison.

Group	N	Genes
Upregulated DEGs	422	LOC106016138, LOC119717431, LOC119718627, LOC119715673, LOC119715300, OLFM4, VPREB3, LOC101794061, LOC101795471, LOC119716924, NKX2-5, LOC119714867, LOC119714865, LOC101802977, CDH6, LOC119715236, CD72, MUC16, LOC119718509, LOC113840514, LOC101791268, MZB1, SYT8, LOC119714450, TNFRSF13C, CD22, LOC119714447, DTX1, LOC113842527, LOC113843481, PAX5, TMEM196, LOC101797788, LOC119715492, CXCR5, MINDY4B, ATP6V1B1, LOC119714863, LOC119716808, LOC106017715, LOC101794304, FAM135B, LOC106018249, LOC119716133, MEF2B, CPLX3, LOC119715235, AICDA, LOC101800964, NR5A1, LOC119714449, LOC101799803, IKZF3, LOC106017353, STK31, HSD17B2, LOC101803936, LOC101792387, LOC101801498, CARMIL2, DSCAM, RASAL1, LOC119714861, LOC119716116, TECRL, LOC101792733, LOC119713782, TNFRSF13B, MUC1, LOC101804519, LOC119714971, PDCD1, POU2AF1, LOC106015683, LOC101805198, LOC101803958, CD79B, LOC106017287, TMEM163, LOC119713850, LOC119715067, LOC101805457, LOC106015026, GNGT2, LOC101796210, LOC113840747,
