# Supplementary material for: Integrated transcriptomic and metabolomic analyses reveal host–metabolite interactions underlying cloacal inflammation in laying Pekin ducks
Source: Anim Biosci. 2026 Apr 16;39(10):260208. doi: 10.5713/ab.260208 (PMC13662949; doi:10.5713/ab.260208)
Supplement: Supplementary file 6 [file ab-260208-Supplementary-3.pdf]

**Supplement 2. Significantly differentially expressed genes in ID vs NID comparison.**

| Group | N | Genes |
|---|---|---|
| Upregulated DEGs | 451 | *LOC119713823, CDH6, ESR2, SIX3, DRD5, MDGA1, LOC106017715, DUSP29, OSTN, TECRL, LOC119713219, LOC113840355, LOC110352957, C7H21orf58, LOC119713220, LOC110351497, LOC113842379, ASB18, TMEM233, LOC101801612, LOC101803316, ASB15, STRIT1, ARX, LOC101791401, LOC101794508, LOC101796474, LOC119713167, LOC101802165, LOC101802071, LOC113844155, MYOM2, GPD1, LOC101794221, ASB4, MAFA, LOC119715992, LOC119713221, GRIN2B, LOC101793478, LOC101790909, PPDPFL, RPL3L, LOC110353953, MYBPH, LOC106016943, LOC119712999, NWD1, DRGX, OBSL1, C2H8orf34, LMOD3, MYLK2, IP6K3, ACTA1, LOC119715271, MYL1, LOC106018919, LOC101801311, LOC106014873, LOC119717776, NT5C1A, MYH7B, LOC106015356, NR4A1, LOC113842688, SMTNL2, LOC119718360, ANKRD2, LOC106017289, C6H10orf71, SLC8A3, CRTAC1, CLCN2, SLC30A2, LOC106015797, USP2, LOC110351399, KCNH7, ASB10, LRRC2, DSCAM, TNNT3, KLHL40, IRX1, TEKT5, LOC119714566, OBSCN, PDK4, LOC113842930, MYLK3, LOC110353597, HSD17B2, IRX2, LOC101791922, LOC119718254, PNMT, LOC119713229, EEF1A2, ERBB4, PLN, ARMC12, LOC101804042, FSD2, LOC101795019, LDB3, KCNK9, CNMD, LOC101795309, USP13, SNCB, CORO6, LOC113840724, LOC113843713, LOC101793493, JPH1, STAC3, PKD2L2, SLN, KLHL30, FHL2, LOC113840410, LOC106019710, LOC101800711, CLDN14, SYNPO2L, FHL3, TRDN, GRIK4, PDE4C, TMEM255B, LOC106015458, LOC106017354, IRS2, NEB, TEKT1, PPP1R1A, LDHD, TTN, LOC119716681, CRYAB, MYO18B, SGCZ, SLC25A4, LOC119715705, LOC119714529, FHL1, RAMP1, LOC101793529, ALPK3, FKBP1B, SMPX, LOC101794749, LOC119714325, LOC119715738, ATP2A2, LOC119716429, LOC119715431, LOC113840188, HSPB7, LOC119716546, LOC110351921, RTN4R, SGCA, LOC110352115, TMEM74, LOC113842743, LOC106016406, MYLK4, BEST3, LOC113845049, LOC119716592, LOC101798971, LOC101799407, TXLNB, CMYA5, LOC119714295, CLSTN2, LOC113841418, TRIM54, LOC101795226, ANO8, LOC101800783, LRRC56, KLHL34, CHRNA1, MSTN, LOC101804425, MYL2, CPT1B, TMEM38A, LOC119718029, FGF18, PRKAB2, LOC119718310, LOC106018705, LOC119717819, RAPGEF4, ACACB, CLCN1, FSD1L, SEMA6B, EYA4, REEP6, LOC119718304, RNF207, FRMD3, LOC113844214, TMOD1, FGF13, LOC119717734, ARPP21, DUSP26, LOC101794131, HSPB1, TPM3, RBM24, IGDCC4, CAMK2A, LOC101790517, LRRC14B, BCAN, LOC113843972, TMEM229B, PCBP4, ADAMTS20, LOC113843890, LRRC20, LOC119717600, KLHL14, RIMBP2, LIMCH1, RXFP2, ANO4, COLQ, SPEG, ASB11, CAP2, LOC113841470, C5H14orf180, LOC106019894, SLC7A2, STRIP2, ANKEF1, LOC101789676, ALPK2, TMEM132D, APOH, LOC119715432, LOC119716801, ABLIM2, CUTA, LOC106016021, TPM2, PGM1, PDLIM5, NRN1L, LAPTM4B, LOC110353809, PABPC4, CAPN3, SIK2, EPDR1, KBTBD13, COQ8A, LSMEM1, IGSF21, LOC110354152, LOC113844903, LOC101797856, HELT, LOC101802901, S100A1, LOC119718301, LOC119715213, DKK2, TMEM52, RANBP3L, STYXL2, LRRC38, LOC101793418, DNAJB13, MLF1, LOC101794949, KCNH2, FKBP3, LOC119715435, LOC119713150, GMPR, LOC101792067, ADCY2, SLC38A3, RYR3, TMOD4, PHKA1, FZD9, NEXN, LOC110351385, LOC101804257, RGS11, POPDC2, KCNJ2, ADSS1, RAPGEF5, PCP4, LOC119718305, KCND2, LOC101802516, SH3BP5, LOC110352082, LOC101794644, SPTB, LOC119718300, LOC106015023, LRRTM4, SLC25A34, LOC113845281, NR4A3, NSUN7, PDZK1IP1, TEAD4, LOC101804932, HS3ST2, CNTNAP5, NMNAT3, STC2, BOLL, GAMT, LOC119718096, NDRG4, BIN1, LOC119717257, PLP1, LOC119714067, RALY, COQ10A, BCKDHB, FBXO15, LOC113843748, CEP85, PLPPR1, MINAR1, PPDPF, GCM1, LOC119717767, LOC119718309, AJM1, REEP1, LOC101791673, MAPK8IP1, ACVR1C, FAM162B, PKIA, KCNT1, PRSS12, RASGEF1B, LOC106018273, LOC119714223, WFIKKN1, SNX22, DES, SESN2, SSUH2, HSPB8, SLC6A8, LOC101801369, MCRIP2, ACYP2, FILIP1, PLCXD2, LOC101796791, ZNF106, GPAM, SCN2B, ZBTB16, CLTCL1, TM6SF1, PIWIL1, LOC113843555, PM20D2, LOC119714192, LOC113845031, MCF2, TBX18, unassigned_gene_1, WIPF3, LOC119718408, PEBP4, LOC106015647, RETREG1, PRKAG3, LOC101802576, PLCD4, LOC101802038, EPM2A, PDK2, GPC3, unassigned_gene_11, GPM6B, SIK1, PDE4B, PKNOX2, KHDRBS3, IRS1, LOC101791581, unassigned_gene_12, LOC119716632, JPH2, SLC25A12, LOC113844749, LOC106015025, LOC101793413, FEM1C, SLC27A1, ISM1, FKBP5, LOC106016919, NFATC1, PLCL1, ACO2, LOC101791736, SLC16A7, LOC106014639, ADHFE1, LOC119717011, CD320, TPPP3, ELAVL4, ACADL, NSMF, SGCD, LRRC39, TMEM82, MTCL1, AQP4, CISD3, SLC25A33, SYNM, TMEM109, RBM38, AGBL3, CACNA2D1, PLPP7, SCYL3, SPRY2, LOC119718147, CFL2, SH2B2, FXR1, PPM1L, PNPLA2* |
| Downregulated DEGs | *436* | *LOC106019132, LOC101798959, LOC101793057, LOC110354560, LOC110351995, LOC119713829, LOC101802909, B4GALT6, LOC101799898, LOC101803727, LOC101803082, LOC101791569, IL8, C8B, BMP3, LOC119714395, SYT8, LOC101796665, COL11A1, SAA2, LOC119714069, LOC101801508, LOC106014594, LOC101802211, SPP1, LOC110351193, LOC101800424, LOC101801676, LOC119717029, CTHRC1, LOC101794528, ZP1, LOC101793676, LOC113840231, LOC119717042, LOC119717040, TNC, PRLH, LOC101793441, CHRNA4, WNT16, MINDY4B, C1QTNF8, LOC119713475, SERTM2, LOC113843959, COL6A6, LOC119717036, TLR7, LOC119718349, LOC101804519, PTGS2, LOC101793957, PDCD1, TMEM45A, LOC101795539, CCL26, LOC101797788, NKX6-2, LOC119713916, LOC101791385, LOC106019940, INHBA, CD22, SLC2A6, LOC101793778, LOC119715005, EVX1, LOC113842721, LOC101790301, LOC101793878, PIRT, LOC119717888, LOC101803817, PRAM1, LOC119714144, DCHS2, LOC101802072, TMEM26, DRD4, LOC101797500, LOC106017965, LOC119718509, LOC101794304, MLKL, ADAMTS18, TNFRSF13B, LOC113845350, ESM1, LOC106015430, LOC101800358, LOC101791674, SRMS, HPGDS, LOC119717041, ACOD1, MZB1, LOC119713140, COL1A1, LOC101791902, FAM163A, ASS1, BRSK2, LOC119717944, MPEG1, LOC119713095, LOC101801783, LOC101798845, DCX, C3, ZNF831, TLR1-A, GP1BA, LOC101803823, COL3A1, GAP43, PAX6, ADAM8, CENPA, LOC101789851, LOC119714296, LOC113841492, GHRH, PTX3, ADAMTSL2, LOC113845274, LOC119712955, LOC101795569, LOC110353216, LOC101801606, GPR18, LOC101798264, LOC119715439, COL1A2, CCR10, HAS3, LOC101796765, FKBP11, ADAM12, LOC119713036, CST7, LOC119714156, ATP4A, LOC113841770, ADAMTS12, LOC101802760, DTX1, TNFSF8, MMP9, LOC101793260, LOC101805457, CFAP251, GPR132, MARCO, LOC101794299, LOC101800757, DDC, LOC106018497, CD247, CCL22, LOC101801498, GNG2, UBE2C, COL5A1, LOC119718666, EOMES, CA10, KDELR3, SPARC, DNAJC5B, PCBD1, LOC101797052, LOC106019440, LOC119714803, ELN, CKS1B, ADAMTS2, LOC119718710, CXCR4, CD3E, IL21R, GPX7, LOC119714651, LOC119717889, LOC119713033, LOC113840033, LOC101797666, CFAP46, LOC101804783, CARD9, CRYBB1, RCN1, LOC113842387, LOC119718662, FSCN1, LOC119713971, BATF, DBH, LOC113839921, LOC106020629, LOC106020111, POU2AF1, LOC101797566, TASL, MSX1, COL26A1, LOC101802873, LOC101803429, CNRIP1, ABHD8, KCNG2, CDCA3, LOC101800311, SHMT2, LOC101798133, LOC101803633, ADAMTS15, LOC101792171, ITGB2, TRIM71, LCP1, LOC119715859, PLK1, CASR, RHOH, TSPEAR, FBN3, LOC119713784, CREB3L1, LY96, LOC119713918, SRGN, LOC119715065, ITK, LOC106017547, PRR11, TAFA3, LOC101803950, TM4SF19, LOC101800429, SLA, SPRY4, LOC101802067, LOC110351762, C1QC, LPXN, IL2RG, FKBP10, MFNG, ADGRG7, VCAM1, LOC101798037, WDFY4, IGSF10, LOC119717051, LOC101803193, IKZF1, IL6, CDH3, LOC101793812, IRF4, GPR65, IL1RL1, LOC119718395, CDCA8, LRRC23, HHIPL1, EVI2A, C3AR1, IGSF6, QPCT, SAMSN1, COL5A2, LOC101795053, CARMIL2, IL18R1, STEAP1, P4HA3, LOC113845409, THSD7A, ABCG4, TLR-15, LOC101791564, CNN2, LOC113840340, LOC101800880, ADAMTS10, SERPINH1, TNFSF11, NOS2, COL6A3, SNAI1, KIF21B, RHEX, RHOG, ALOX5, LOC113843595, LOC101803964, NCF2, EVI2B, PIK3R5, STC1, PTPRC, RGS9, LOC119718678, LOC101797774, LOC119718589, TRAF3IP3, RRM2, KLHL6, LOC119715357, ARHGAP15, IFI30, LOC101791390, LOC113840683, TK1, WNT1, LOC113842636, LOC113843744, ANKRD34A, LOC101796407, LOC101793233, FNDC1, LOC101802747, WSCD1, FBLIM1, LOC101803870, LRRC15, MYBL1, GPRIN3, C1QB, LOC119713249, PPEF1, OMG, ESPL1, LEF1, VCAN, GPER1, PTCHD3, TDRD5, LOC101789766, DNAH1, LOC101800726, LOC113843513, CCL19, LOC101804340, CD180, LOC119714196, RUNX3, IRAG2, KPNA2, COL9A2, ARHGAP9, CARD11, LOC119713956, LOC101797250, LOC101793086, ST8SIA1, TNIP3, LOC119716063, PEAK3, DOCK2, C1QTNF6, CENPO, LOC101791991, LOC119714135, CCN4, DOK2, CKAP4, LOC101795552, FN1, LOC119717472, LOC101799520, GJC2, PIF1, C1QA, TUBB3, SH3TC2, TRARG1, SGIP1, TTLL10, ADGRG5, CX3CR1, LOC113845767, PIK3CD, CYFIP2, ABI3, FBN2, LOC101791359, FKBP7, LOC101792402, SASH3, DNASE2B, LCK, E2F1, CEP55, LOC101803634, TLR4, DEPDC1B, CCNB3, LOC119716678, LOC101792606, LOC101792124, LOC119714503, LAMP3, CD40LG, CADM3, KIF14, NDC80, PRSS35, LOC101793940, RGS19, PTTG1, LOC110351185, LOC106019771, DEPDC1, LOC119713016, LOC106014292, P2RY13, LOC101805472, LOC101804178, ST6GALNAC5, C6H10orf95, PXYLP1, LIMD2, SKA1, LOC113845763, COL6A1, TMEM71, LOC106018390, LAMP5, KIF18B, XYLT1, LUM, LOC119713786, LOC119713674* |

**Supplement 3. Significantly differentially expressed genes in ID vs HC comparison.**

| Group | N | Genes |
|---|---|---|
| Upregulated DEGs | 422 | *LOC106016138, LOC119717431, LOC119718627, LOC119715673, LOC119715300, OLFM4, VPREB3, LOC101794061, LOC101795471, LOC119716924, NKX2-5, LOC119714867, LOC119714865, LOC101802977, CDH6, LOC119715236, CD72, MUC16, LOC119718509, LOC113840514, LOC101791268, MZB1, SYT8, LOC119714450, TNFRSF13C, CD22, LOC119714447, DTX1, LOC113842527, LOC113843481, PAX5, TMEM196, LOC101797788, LOC119715492, CXCR5, MINDY4B, ATP6V1B1, LOC119714863, LOC119716808, LOC106017715, LOC101794304, FAM135B, LOC106018249, LOC119716133, MEF2B, CPLX3, LOC119715235, AICDA, LOC101800964, NR5A1, LOC119714449, LOC101799803, IKZF3, LOC106017353, STK31, HSD17B2, LOC101803936, LOC101792387, LOC101801498, CARMIL2, DSCAM, RASAL1, LOC119714861, LOC119716116, TECRL, LOC101792733, LOC119713782, TNFRSF13B, MUC1, LOC101804519, LOC119714971, PDCD1, POU2AF1, LOC106015683, LOC101805198, LOC101803958, CD79B, LOC106017287, TMEM163, LOC119713850, LOC119715067, LOC101805457, LOC106015026, GNGT2, LOC101796210, LOC113840747,* |

| | | |
|---|---|---|
| | | LOC119713297, LOC119715510, LOC119714864, LOC119715705, LOC113845383, LOC119714994, SLC13A1, DRD5, LOC119717100, PTPN7, RHOH, LOC113845412, LOC119717909, LOC119715065, LOC113841770, CYFIP2, ESPN, LOC119715072, LOC101800913, ABI3, LOC119717131, LOC101801160, LAG3, LOC119718254, LOC119718611, TNR, LOC106015647, LOC119717144, ATP4A, LOC119715074, LOC113840654, LOC119713026, GCM1, LOC113839930, LOC119718505, DMBT1, DNAI3, LOC119715490, LOC101805432, LOC110352865, LOC106016299, LOC119717604, BLK, LOC119713039, TMEM269, LOC113842161, LOC119718589, CDH9, CNMD, PIGR, LOC119715075, GPR15, LOC106017126, WDFY4, LOC119718507, LOC106017520, LOC113844476, GPR18, MYBL1, LOC113841630, ATP2A3, LOC101795529, LOC119715364, EGR3, WDR88, LOC113840509, LOC119715468, ZNF831, SLC5A8, LOC113845395, LIX1, SLC45A3, CDH17, LOC119715429, C10HXorf65, LOC101803387, LOC101796759, ST8SIA6, KSR2, CST7, ALB, BATF, LOC119718408, LOC101804790, LOC110352957, LOC119715363, LOC119715172, IL21, XPNPEP2, LOC101790952, LOC119715069, LOC119715357, LOC119714996, LOC113841604, GATA5, LOC119714143, LOC113842282, NLRC3, LOC113841232, LOC101795309, LOC101791390, KLHL6, C2CD4C, PPFIA4, CD6, LOC106016902, ZGRF1, CD96, RAC2, EDAR, LOC119716370, LPXN, LOC101797718, CNTFR, LOC119717899, LIME1, KIAA0040, GFRA1, IRF4, LOC119714894, LOC113842163, CRYBB3, FLT3, FUT9, LOC101791534, ISLR2, LOC106017673, LOC106014495, CCL20, LOC119716538, RASGRP3, CCND3, VSIG10, LOC119714871, LOC101790341, LOC119717732, LOC119713784, MADCAM1, LOC113840447, LOC101800207, LOC101791564, P2RX5, GMPR, IRAG2, NFASC, TLR1-A, BTK, TRAF3IP3, SLCO4A1, LOC106015023, LOC113841901, HMCES, CD180, LOC119714819, LOC113841838, LOC119715242, ARR3, LOC113841629, CIITA, LOC119713918, RAD54L, PDZK1IP1, ACSL5, LOC101803073, LOC119714744, LOC113845716, LOC101792606, LOC101804042, RUNX3, CCR6, MCM5, SASH3, LOC110354615, LOC119715916, LVRN, LOC119715070, IL21R, LOC113843513, LOC119713851, LOC101793029, ABCG2, RGS6, CFAP206, NUP210, DNTT, ARHGAP9, LIMD2, LOC101792885, LOC101802373, LOC119714854, DOK3, DMRT1, ENTPD8, LOC101793940, GPR55, LOC119714866, CFAP73, LOC119712979, MYO1H, LOC113845717, KLHDC7A, GPR3, LRAT, LOC106015903, LOC113845438, LOC113841777, IGSF9B, LOC119718654, LOC119713919, GRIK3, KLHL34, CSK, SLC5A1, LOC119713299, ALKBH2, CDCA7, UNG, LOC113839605, SPAG6, LOC119718506, CFTR, LOC119717868, LOC119716486, SH2B2, ATP7B, PRPS2, CD8A, LOC113841322, LOC101798418, LOXHD1, LOC101792763, LOC119718310, KIAA0895L, LOC119714818, PIK3AP1, LOC119717729, TMEFF1, LOC119714515, LOC119717226, LOC101802936, PLCG2, VWC2, LYG2, LOC101795581, CHTF18, CLDN10, LOC119718068, LOC119717843, LOC101799758, LOC113845763, LOC101795032, LOC113840542, STXBP5L, LOC106015872, LOC113840606, LOC119714164, UNC93A, NTN1, GDF1, LOC119716561, LOC101798815, LOC113845608, VWA5B1, LOC119713258, LOC106014553, LOC101794002, SLC16A14, ELMO1, SIAH2, LOC113839872, FANCC, TDRD6, LOC119717548, LOC110353167, LOC119714870, FANCD2, LYPD6B, DIRAS2, FANCE, ASCL4, CSMD2, LOC101805264, ANOS1, BLNK, LOC119713029, ARHGAP24, LOC110351392, TENT5C, DOC2B, LOC119714984, LOC113845703, SLC9A7, CRTAC1, CNR2, POLH, LOC119716307, LOC110351921, LOC119713921, SCTR, LOC113843865, LOC113845718, LOC113841845, PIK3C2B, FIGNL1, GINS1, AMDHD1, PPP1R16B, LOC106015834, CHKA, HVCN1, DRP2, GRIN1, LOC113844663, VOPP1, NEU4, LOC106017059, LRRC26, NLGN3, RUBCN, LOC113841603, GRHPR, DENND5B, TMPRSS2, LOC101791581, E2F2, MCM8, LOC119715912, PITPNM2, LOC113840481, LOC101794526, SYT7, ZNF608, SLC17A9, RHOF, LOC119715619, LOC101790356 |
| Downregulated DEGs | 315 | LOC113839716, LOC101798959, LOC106019132, LOC110354560, PTX3, IL8, LOC101800424, LOC101803082, CTHRC1, PAPPA2, COL11A1, CYTL1, CACNB4, LOC101793676, SPP1, LOC101793057, NPBWR2, LOC106017735, LOC101801676, C1QTNF8, LOC113840231, LOC101799579, LOC119714069, ZP1, LOC101795539, LOC119713475, ACE, LOC110351193, LOC101802909, COL1A1, LOC119714651, LOC106015430, TMEM45A, LOC101793878, LOC110351189, RBP7, ADAM12, LOC101798298, LOC113842678, LOC101793561, LOC101796665, COL1A2, TMEM26, COL3A1, LOC101797088, LOC101793441, WNT16, ELN, COL5A1, HPGDS, PDE5A, LOC113843595, FNDC1, LOC119714042, PTGS2, GHRH, LOC101795465, GPX7, LOC101800726, LOC101793812, LOC119715005, FN1, LOC101792626, LOC119717042, LOC101791902, SPARC, LOC101797500, VCAN, TNC, LOC101797566, LOC101793233, ADAMTS2, LOC106018497, LOC119715604, LOC113840033, MARCO, ADAMTS12, DBX2, LOC119713290, LOC113843959, LOC101803823, COL5A2, LOC119718232, KDELR3, LOC101797052, LOC101802072, ADAMTS15, LUM, LOC113839922, SUSD5, LOC110352522, CUBN, COL20A1, COL6A3, LOC101801508, PRG4, LOC110352931, TLR5, LOC110353218, LOC101790657, SIGLEC1, RCN1, G0S2, TRNAR-CCG, LOC119715018, KCNG2, MCF2L2, SV2C, LOC119713095, LOC101792918, FOXL2, CCL26, TNFAIP6, COL6A1, MATN3, LOC119718185, LOC113841475, FKBP7, HHIPL1, OAF, CCN4, COL8A1, PPEF1, COL6A2, FKBP10, LOC101799230, SERPINH1, GLT8D2, PRDM8, IGF1, ST8SIA2, NRN1, ESM1, P4HA3, LRRC15, LOC101792402, QPCT, LOC101795107, DNAH1, CFAP46, RGS16, CRMP1, LOC119713872, MMP23B, LOC101793957, COL5A3, LOC113841362, LOC101801783, LOC113839921, OGN, LOC119714291, LOC101797569, GP1BA, ABHD8, CIDEC, LOC119718602, LOC119717176, WFDC1, FBN1, ARHGAP22, LOC119715976, LOC101794299, FBLN2, FBN3, LOC101804074, GAL, LOC119713616, LOC101802744, LOC101797203, F2, LOC101803098, LOC119717889, FMOD, LOC113839941, ADGRG7, LOC101804640, LOC101789766, MMP17, PROK2, METTL21C, ANKRD34A, PCBD1, LOC106015640, IL13RA2, RFX4, LOX, OLFML3, CARD9, DMC1, C1QTNF6, SCG3, MFAP2, OTOS, LOC101801190, INHBA, FGL2, HAS3, RAB23, FAM81A, LOC119713856, PODN, BAG2, LOC119717573, LOC119713225, CCBE1, IGSF10, LOC101802067, LOC101799146, LOC119718712, NOX3, SGIP1, LOC106018389, LOC101794319, TTC39A, LOC101798499, OLFML2B, WSCD1, LOC113840744, LOC101794631, SERPINF1, ALOX5, LOC101790299, LOC101804173, FBLIM1, COL12A1, CCDC80, MSH4, LOC119717559, TGFBI, LOC101799516, C1QC, COL14A1, DYNLT5, ADAM8, LOC101797243, ST6GALNAC5, ANGPTL5, CKAP4, LOC101799623, CREB3L1, LOC119713729, ABCB11, ZNF469, LOC106014509, LOXL2, LINGO1, THBS3, LOC101793253, KCNE4, C1QTNF2, P4HA2, B3GNT9, TRPA1, LOC101796528, INSYN1, TLR4, LOC101804178, PKDCC, KERA, LOC119716063, LOC101789401, LOC119718344, LOC110351185, CRLF1, LOC101798468, LOC101789696, RRBP1, LOC101800293, PXDN, LOC113840193, LOC119717472, LOC101794246, LOC101795053, LOC101796516, LOC106016655, MRC2, LOC101792124, LOC101796964, IL1RL1, PTPRR, RARRES1, PRDM5, LOXL3, LOC119716104, LOC119716034, LOC119716109, EDNRA, COL16A1, DCN, TSKU, THY1, LOC119713179, HTRA3, LOC119713469, COL9A2, LOC110353965, RFESD, COL7A1, HGF, CASR, HHIPL2, ZFPM1, GLIS3, LOC119713249, C1QA, RNF157, TMEM119, DMGDH, HECW1, LOC113843291, ENO2, LOC119714803, SLC6A13, C1QB, WDR86 |

## Supplement 4. Significantly differentially expressed genes in NID vs HC comparison.

| Group | N | Genes |
|---|---|---|
| Upregulated DEGs | 1002 | VPREB3, LOC119718509, CD72, LOC119718627, LOC113843481, LOC119714447, PAX5, CD22, CXCR5, MINDY4B, LOC101795471, MZB1, SYT8, LOC101794061, LOC101797788, LOC119714449, DTX1, LOC101794304, LOC119714450, LOC101792733, RASAL1, LOC119716808, LOC101805198, PDCD1, LOC101801254, LOC119717774, TNFRSF13C, LOC101796210, LOC101803936, AICDA, LOC106018249, TNFRSF13B, LOC119713782, LOC119716924, LOC101802211, LOC119715463, MUC5AC, HCN4, LOC113840514, LOC101796759, LOC119716371, LOC101801498, LOC101794528, LOC119715236, LOC110351664, LOC101791268, IKZF3, LOC119714867, CARMIL2, LOC119714971, LOC119715235, LOC119714865, LOC101804581, LOC101800964, MEF2B, LOC101805457, POU2AF1, LOC101792344, LOC101804519, MUC16, LOC119716405, CD79B, LOC113841770, LAG3, NKX2-5, CPLX3, MUC1, ATP4A, PTPN7, LOC119713297, ATP6V1B1, LOC119715300, LOC113845335, SLC13A5, SAA2, ZNF831, RHOH, LOC119715065, GPR18, LOC119717131, CTLA4, SERTM2, LOC119715673, NEUROD6, LOC110352525, LOC119715510, LOC101799803, LOC119714863, LOC113839930, LOC106015026, LOC113842527, LOC119717732, LOC119714118, CST7, LOC101804866, TMEM163, LOC106016891, LOC119716116, STK31, LOC101792387, CYFIP2, ANO7, ABI3, LOC119714143, LOC119715072, LOC101795976, WDFY4, TLR1-A, LOC113844068, BATF, LOC119718589, LOC119715067, LOC106017353, LOC119717733, LOC119715492, LOC101805061, LOC106015683, CD6, MYBL1, C10HXorf65, LOC119714820, LOC106014594, BRSK2, LOC110352865, LOC119714969, KNG1, LOC119714861, LOC119713026, LOC101803958, LOC106017126, LOC113841930, BLK, LOC119717932, LOC119715468, LPXN, LOC119715357, LOC119714821, LOC113840747, LOC101791390, LOC101801160, IRF4, KLHL6, ATP2A3, LOC119717054, ADAD1, GNGT2, LOC119713784, LOC119715073, NLRC3, CD4, IL21R, FOXF2, LOC113845274, LOC119714837, LOC119714994, LOC106014904, ATP6V0A4, CDH17, TERT, TSPEAR, LOC119713918, LOC119714259, LOC113843625, LOC101791564, LOC106020056, LOC119714144, LOC119717048, LOC119715477, SPIC, LOC101802760, EGR3, LOC113841233, LOC119718505, LOC119713140, LIX1, CD5, LOC119715066, TRAF3IP3, LOC119714864, LOC119715074, LOC119715429, LOC113845412, LOC106017287, LOC101789851, LOC113842891, IRAG2, DMBT1, LOC101800913, TBXT, LOC119715075, LOC119714950, LOC101798133, LOC113841630, CD180, XPNPEP2, LOC119713850, LOC113840654, SLC2A6, CENPA, LOC119714117, LOC119717899, LOC119715363, LOC113845383, LOC119714826, LOC119717144, LOC119717100, CD96, NR5A1, LOC119713369, RUNX3, IL2RG, TCF7, SLC13A1, VWDE, LOC113843513, LIME1, LOC113840863, LOC119715490, TNR, RHOG, IL21, ZGRF1, ARHGAP9, CXCR4, SLC45A3, THEMIS, LOC101792606, LOC101803073, RAC2, LOC110351249, SASH3, TASL, MFNG, CIITA, LOC119715068, LOC113845395, CCR7, LOC119716137, LOC106019826, LOC101803193, GATA5, LOC101790341, CCND3, LOC119713036, LOC101793940, LIMD2, TXK, UBE2C, LOC119714512, SH2D1A, KCNK12, FLT3, KSR2, LOC119714157, LOC113840509, LOC119712955, KIAA0040, BTK, ALB, LOC113839910, GPR15, ARR3, KIF21B, LOC101798888, AQP5, ABCG4, LOC119715164, CD8B, BCL2L14, CCR10, LOC101795529, MCM5, CDHR5, NUP210, LOC101793029, LOC110353216, LOC101804783, LOC101790952, ST8SIA6, LOC119714128, LOC101802279, IL22RA2, ESPN, LOC119717604, LOC119715364, LOC119715163, LOC119715069, LOC101793096, CCL20, LOC119717605, LOC119718654, LOC119718507, FUT9, LOC113841492, LOC113841164, LOC119718246, |
