# Supplementary material for: Integrated transcriptomic and metabolomic analyses reveal host–metabolite interactions underlying cloacal inflammation in laying Pekin ducks
Source: Anim Biosci. 2026 Apr 16;39(10):260208. doi: 10.5713/ab.260208 (PMC13662949; doi:10.5713/ab.260208)
Supplement: Supplementary file 7 [file ab-260208-Supplementary-4.pdf]

		LOC119713297, LOC119715510, LOC119714864, LOC119715705, LOC113845383, LOC119714994, SLC13A1, DRD5, LOC119717100, PTPN7, RHOH, LOC113845412, LOC119717909, LOC119715065, LOC113841770, CYFIP2, ESPN, LOC119715072, LOC101800913, ABI3, LOC119717131, LOC101801160, LAG3, LOC119718254, LOC119718611, TNR, LOC106015647, LOC119717144, ATP4A, LOC119715074, LOC113840654, LOC119713026, GCM1, LOC113839930, LOC119718505, DMBT1, DNAI3, LOC119715490, LOC101805432, LOC110352865, LOC106016299, LOC119717604, BLK, LOC119713039, TMEM269, LOC113842161, LOC119718589, CDH9, CNMD, PIGR, LOC119715075, GPR15, LOC106017126, WDFY4, LOC119718507, LOC106017520, LOC113844476, GPR18, MYBL1, LOC113841630, ATP2A3, LOC101795529, LOC119715364, EGR3, WDR88, LOC113840509, LOC119715468, ZNF831, SLC5A8, LOC113845395, LIX1, SLC45A3, CDH17, LOC119715429, C10HXorf65, LOC101803387, LOC101796759, ST8SIA6, KSR2, CST7, ALB, BATF, LOC119718408, LOC101804790, LOC110352957, LOC119715363, LOC119715172, IL21, XPNPEP2, LOC101790952, LOC119715069, LOC119715357, LOC119714996, LOC113841604, GATA5, LOC119714143, LOC113842282, NLRC3, LOC113841232, LOC101795309, LOC101791390, KLHL6, C2CD4C, PPFIA4, CD6, LOC106016902, ZGRF1, CD96, RAC2, EDAR, LOC119716370, LPXN, LOC101797718, CNTFR, LOC119717899, LIME1, KIAA0040, GFRA1, IRF4, LOC119714894, LOC113842163, CRYBB3, FLT3, FUT9, LOC101791534, ISLR2, LOC106017673, LOC106014495, CCL20, LOC119716538, RASGRP3, CCND3, VSIG10, LOC119714871, LOC101790341, LOC119717732, LOC119713784, MADCAM1, LOC113840447, LOC101800207, LOC101791564, P2RX5, GMPR, IRAG2, NFASC, TLR1-A, BTK, TRAF3IP3, SLCO4A1, LOC106015023, LOC113841901, HMCES, CD180, LOC119714819, LOC113841838, LOC119715242, ARR3, LOC113841629, CIITA, LOC119713918, RAD54L, PDZK1IP1, ACSL5, LOC101803073, LOC119714744, LOC113845716, LOC101792606, LOC101804042, RUNX3, CCR6, MCM5, SASH3, LOC110354615, LOC119715916, LVRN, LOC119715070, IL21R, LOC113843513, LOC119713851, LOC101793029, ABCG2, RGS6, CFAP206, NUP210, DNTT, ARHGAP9, LIMD2, LOC101792885, LOC101802373, LOC119714854, DOK3, DMRT1, ENTPD8, LOC101793940, GPR55, LOC119714866, CFAP73, LOC119712979, MYO1H, LOC113845717, KLHDC7A, GPR3, LRAT, LOC106015903, LOC113845438, LOC113841777, IGSF9B, LOC119718654, LOC119713919, GRIK3, KLHL34, CSK, SLC5A1, LOC119713299, ALKBH2, CDCA7, UNG, LOC113839605, SPAG6, LOC119718506, CFTR, LOC119717868, LOC119716486, SH2B2, ATP7B, PRPS2, CD8A, LOC113841322, LOC101798418, LOXHD1, LOC101792763, LOC119718310, KIAA0895L, LOC119714818, PIK3AP1, LOC119717729, TMEFF1, LOC119714515, LOC119717226, LOC101802936, PLCG2, VWC2, LYG2, LOC101795581, CHTF18, CLDN10, LOC119718068, LOC119717843, LOC101799758, LOC113845763, LOC101795032, LOC113840542, STXBP5L, LOC106015872, LOC113840606, LOC119714164, UNC93A, NTN1, GDF1, LOC119716561, LOC101798815, LOC113845608, VWA5B1, LOC119713258, LOC106014553, LOC101794002, SLC16A14, ELMO1, SIAH2, LOC113839872, FANCC, TDRD6, LOC119717548, LOC110353167, LOC119714870, FANCD2, LYPD6B, DIRAS2, FANCE, ASCL4, CSMD2, LOC101805264, ANOS1, BLNK, LOC119713029, ARHGAP24, LOC110351392, TENT5C, DOC2B, LOC119714984, LOC113845703, SLC9A7, CRTAC1, CNR2, POLH, LOC119716307, LOC110351921, LOC119713921, SCTR, LOC113843865, LOC113845718, LOC113841845, PIK3C2B, FIGNL1, GINS1, AMDHD1, PPP1R16B, LOC106015834, CHKA, HVCN1, DRP2, GRIN1, LOC113844663, VOPP1, NEU4, LOC106017059, LRRC26, NLGN3, RUBCN, LOC113841603, GRHPR, DENND5B, TMPRSS2, LOC101791581, E2F2, MCM8, LOC119715912, PITPNM2, LOC113840481, LOC101794526, SYT7, ZNF608, SLC17A9, RHOF, LOC119715619, LOC101790356
Downregulated DEGs	315	*LOC113839716, LOC101798959, LOC106019132, LOC110354560, PTX3, IL8, LOC101800424, LOC101803082, CTHRC1, PAPPA2, COL11A1, CYTL1, CACNB4, LOC101793676, SPP1, LOC101793057, NPBWR2, LOC106017735, LOC101801676, C1QTNF8, LOC113840231, LOC101799579, LOC119714069, ZP1, LOC101795539, LOC119713475, ACE, LOC110351193, LOC101802909, COL1A1, LOC119714651, LOC106015430, TMEM45A, LOC101793878, LOC110351189, RBP7, ADAM12, LOC101798298, LOC113842678, LOC101793561, LOC101796665, COL1A2, TMEM26, COL3A1, LOC101797088, LOC101793441, WNT16, ELN, COL5A1, HPGDS, PDE5A, LOC113843595, FNDC1, LOC119714042, PTGS2, GHRH, LOC101795465, GPX7, LOC101800726, LOC101793812, LOC119715005, FN1, LOC101792626, LOC119717042, LOC101791902, SPARC, LOC101797500, VCAN, TNC, LOC101797566, LOC101793233, ADAMTS2, LOC106018497, LOC119715604, LOC113840033, MARCO, ADAMTS12, DBX2, LOC119713290, LOC113843959, LOC101803823, COL5A2, LOC119718232, KDELR3, LOC101797052, LOC101802072, ADAMTS15, LUM, LOC113839922, SUSD5, LOC110352522, CUBN, COL20A1, COL6A3, LOC101801508, PRG4, LOC110352931, TLR5, LOC110353218, LOC101790657, SIGLEC1, RCN1, G0S2, TRNAR-CCG, LOC119715018, KCNG2, MCF2L2, SV2C, LOC119713095, LOC101792918, FOXL2, CCL26, TNFAIP6, COL6A1, MATN3, LOC119718185, LOC113841475, FKBP7, HHIPL1, OAF, CCN4, COL8A1, PPEF1, COL6A2, FKBP10, LOC101799230, SERPINH1, GLT8D2, PRDM8, IGF1, ST8SIA2, NRN1, ESM1, P4HA3, LRRC15, LOC101792402, QPCT, LOC101795107, DNAH1, CFAP46, RGS16, CRMP1, LOC119713872, MMP23B, LOC101793957, COL5A3, LOC113841362, LOC101801783, LOC113839921, OGN, LOC119714291, LOC101797569, GP1BA, ABHD8, CIDEC, LOC119718602, LOC119717176, WFDC1, FBN1, ARHGAP22, LOC119715976, LOC101794299, FBLN2, FBN3, LOC101804074, GAL, LOC119713616, LOC101802744, LOC101797203, F2, LOC101803098, LOC119717889, FMOD, LOC113839941, ADGRG7, LOC101804640, LOC101789766, MMP17, PROK2, METTL21C, ANKRD34A, PCBD1, LOC106015640, IL13RA2, RFX4, LOX, OLFML3, CARD9, DMC1, C1QTNF6, SCG3, MFAP2, OTOS, LOC101801190, INHBA, FGL2, HAS3, RAB23, FAM81A, LOC119713856, PODN, BAG2, LOC119717573, LOC119713225, CCBE1, IGSF10, LOC101802067, LOC101799146, LOC119718712, NOX3, SGIP1, LOC106018389, LOC101794319, TTC39A, LOC101798499, OLFML2B, WSCD1, LOC113840744, LOC101794631, SERPINF1, ALOX5, LOC101790299, LOC101804173, FBLIM1, COL12A1, CCDC80, MSH4, LOC119717559, TGFBI, LOC101799516, C1QC, COL14A1, DYNLT5, ADAM8, LOC101797243, ST6GALNAC5, ANGPTL5, CKAP4, LOC101799623, CREB3L1, LOC119713729, ABCB11, ZNF469, LOC106014509, LOXL2, LINGO1, THBS3, LOC101793253, KCNE4, C1QTNF2, P4HA2, B3GNT9, TRPA1, LOC101796528, INSYN1, TLR4, LOC101804178, PKDCC, KERA, LOC119716063, LOC101789401, LOC119718344, LOC110351185, CRLF1, LOC101798468, LOC101789696, RRBP1, LOC101800293, PXDN, LOC113840193, LOC119717472, LOC101794246, LOC101795053, LOC101796516, LOC106016655, MRC2, LOC101792124, LOC101796964, IL1RL1, PTPRR, RARRES1, PRDM5, LOXL3, LOC119716104, LOC119716034, LOC119716109, EDNRA, COL16A1, DCN, TSKU, THY1, LOC119713179, HTRA3, LOC119713469, COL9A2, LOC110353965, RFESD, COL7A1, HGF, CASR, HHIPL2, ZFPM1, GLIS3, LOC119713249, C1QA, RNF157, TMEM119, DMGDH, HECW1, LOC113843291, ENO2, LOC119714803, SLC6A13, C1QB, WDR86*

Supplement 4. Significantly differentially expressed genes in NID vs HC comparison.

Group	N	Genes
Upregulated DEGs	1002	*VPREB3, LOC119718509, CD72, LOC119718627, LOC113843481, LOC119714447, PAX5, CD22, CXCR5, MINDY4B, LOC101795471, MZB1, SYT8, LOC101794061, LOC101797788, LOC119714449, DTX1, LOC101794304, LOC119714450, LOC101792733, RASAL1, LOC119716808, LOC101805198, PDCD1, LOC101801254, LOC119717774, TNFRSF13C, LOC101796210, LOC101803936, AICDA, LOC106018249, TNFRSF13B, LOC119713782, LOC119716924, LOC101802211, LOC119715463, MUC5AC, HCN4, LOC113840514, LOC101796759, LOC119716371, LOC101801498, LOC101794528, LOC119715236, LOC110351664, LOC101791268, IKZF3, LOC119714867, CARMIL2, LOC119714971, LOC119715235, LOC119714865, LOC101804581, LOC101800964, MEF2B, LOC101805457, POU2AF1, LOC101792344, LOC101804519, MUC16, LOC119716405, CD79B, LOC113841770, LAG3, NKX2-5, CPLX3, MUC1, ATP4A, PTPN7, LOC119713297, ATP6V1B1, LOC119715300, LOC113845335, SLC13A5, SAA2, ZNF831, RHOH, LOC119715065, GPR18, LOC119717131, CTLA4, SERTM2, LOC119715673, NEUROD6, LOC110352525, LOC119715510, LOC101799803, LOC119714863, LOC113839930, LOC106015026, LOC113842527, LOC119717732, LOC119714118, CST7, LOC101804866, TMEM163, LOC106016891, LOC119716116, STK31, LOC101792387, CYFIP2, ANO7, ABI3, LOC119714143, LOC119715072, LOC101795976, WDFY4, TLR1-A, LOC113844068, BATF, LOC119718589, LOC119715067, LOC106017353, LOC119717733, LOC119715492, LOC101805061, LOC106015683, CD6, MYBL1, C10HXorf65, LOC119714820, LOC106014594, BRSK2, LOC110352865, LOC119714969, KNG1, LOC119714861, LOC119713026, LOC101803958, LOC106017126, LOC113841930, BLK, LOC119717932, LOC119715468, LPXN, LOC119715357, LOC119714821, LOC113840747, LOC101791390, LOC101801160, IRF4, KLHL6, ATP2A3, LOC119717054, ADAD1, GNGT2, LOC119713784, LOC119715073, NLRC3, CD4, IL21R, FOXF2, LOC113845274, LOC119714837, LOC119714994, LOC106014904, ATP6V0A4, CDH17, TERT, TSPEAR, LOC119713918, LOC119714259, LOC113843625, LOC101791564, LOC106020056, LOC119714144, LOC119717048, LOC119715477, SPIC, LOC101802760, EGR3, LOC113841233, LOC119718505, LOC119713140, LIX1, CD5, LOC119715066, TRAF3IP3, LOC119714864, LOC119715074, LOC119715429, LOC113845412, LOC106017287, LOC101789851, LOC113842891, IRAG2, DMBT1, LOC101800913, TBXT, LOC119715075, LOC119714950, LOC101798133, LOC113841630, CD180, XPNPEP2, LOC119713850, LOC113840654, SLC2A6, CENPA, LOC119714117, LOC119717899, LOC119715363, LOC113845383, LOC119714826, LOC119717144, LOC119717100, CD96, NR5A1, LOC119713369, RUNX3, IL2RG, TCF7, SLC13A1, VWDE, LOC113843513, LIME1, LOC113840863, LOC119715490, TNR, RHOG, IL21, ZGRF1, ARHGAP9, CXCR4, SLC45A3, THEMIS, LOC101792606, LOC101803073, RAC2, LOC110351249, SASH3, TASL, MFNG, CIITA, LOC119715068, LOC113845395, CCR7, LOC119716137, LOC106019826, LOC101803193, GATA5, LOC101790341, CCND3, LOC119713036, LOC101793940, LIMD2, TXK, UBE2C, LOC119714512, SH2D1A, KCNK12, FLT3, KSR2, LOC119714157, LOC113840509, LOC119712955, KIAA0040, BTK, ALB, LOC113839910, GPR15, ARR3, KIF21B, LOC101798888, AQP5, ABCG4, LOC119715164, CD8B, BCL2L14, CCR10, LOC101795529, MCM5, CDHR5, NUP210, LOC101793029, LOC110353216, LOC101804783, LOC101790952, ST8SIA6, LOC119714128, LOC101802279, IL22RA2, ESPN, LOC119717604, LOC119715364, LOC119715163, LOC119715069, LOC101793096, CCL20, LOC119717605, LOC119718654, LOC119718507, FUT9, LOC113841492, LOC113841164, LOC119718246,*

		LOC113840669, LOC113841629, CD3E, LOC101792171, LOC119713069, IKZF1, C8B, ACSL5, LOC119714818, FEV, MADCAM1, TNFSF11, LOC113844476, LOC106019440, LOC101794100, VAV1, LOC113845763, ROS1, DNAI3, LOC101803044, LOC110352617, LOC119718576, LOC119715153, LOC101802936, LOC101803429, LOC106019940, LOC110352696, LOC119714174, LAMP3, MAB21L3, LOC119717868, LOC119714156, DCHS2, LOC119715549, EOMES, CKS1B, CD40LG, B4GALT6, LOC101803387, LOC101805157, KIAA0895L, LOC119716070, LOC101793057, LOC106019771, LOC113842926, CCR6, LOC119714819, LOC119714296, PIK3AP1, LOC113839605, LOC119715566, LOC101798298, LOC101791674, TNFSF8, TLR7, ATP7B, LOC113842282, LOC119717029, PIK3CD, LOC101796159, LOC119714825, LOC119713956, LOC119717898, ARHGAP15, LOC113841001, KLRG1, LOC119715844, RGS19, HMCES, IGSF9B, GPR132, TF, LOC113840683, LOC119715845, LOC101797718, ST8SIA1, P2RY8, UBASH3A, PLD4, LOC119715852, LOC119713053, LOC113841485, ESPL1, EGR2, GSG1L, LOC119717036, LOC113840455, VPS37D, LCP1, GRAP2, LOC113840268, GCNT1, LOC119714503, NCF4, PLCG2, PIGR, GPR55, LOC119716862, LOC101791534, BIN2, LOC119716538, GPRIN3, CRLF2, ENTPD8, GFRA1, VWC2, PTPRC, LOC119716133, MYO1H, CLGN, LOC119714996, LOC101793778, DOK2, LOC101798264, LOC101798985, LOC119713733, E2F1, LOC113845716, LOC113840601, LOC119715493, LOC119718696, LOC106020349, ADGRG5, SAMSN1, ETS1, TMEM269, RAD54L, DOK3, GPR20, ARHGAP45, LOC101800207, PLD5, LOC106016299, EVI2A, IGF2BP3, MGAT4D, ABCG2, KIF18B, LOC101796407, TAGLN, GPR174, BCL2A1, FANCD2, LOC119717729, PLEK, LOC101797126, LOC119717077, SLCO4A1, LOC113842163, LOC101799520, LOC110351806, LOC101792843, LOC101796765, LOC101795007, CRTAM, CARD11, BMP3, LOC101795032, PTPN6, LOC113840542, CD8A, LOC119715070, LOC101794235, GFI1, LOC119713041, LOC101796317, CRYBB3, LOC113842161, LOC119713916, PLK1, FMNL1, CDCA7, NFILZ, LOC119718678, LOC119718710, LOC119715916, LCK, LOC119718285, LOC119713016, LOC119714894, ARHGAP19, LOC101792536, LOC101790301, DDC, CD2, LOC113841838, LOC110352684, ITK, LOC119718506, RASGRP3, LOC119718650, SLC13A2, CD247, HCRT, SIDT1, COLCA2, LOC113841465, LOC106017967, LOC113840976, LOC101792763, SRGN, SIAH2, LOC119712993, FANCC, ITGB2, CNN2, LOC101797210, LOC101799758, LOC113841232, TK1, LRAT, MCM2, SPAG6, TMPRSS7, DOCK2, C2CD4C, SLC4A8, LOC119714147, LCAT, LOC101798037, LYZ, LOC119715228, LOC119714164, LOC101796693, TNFRSF25, LOC113844962, LOC119714163, CD86, PIK3R5, LOC119713674, LOC119714859, LOC119717051, LOC119713785, LOC119713919, LOC113845409, LOC106017520, ITPR3, DNTT, LOC113845350, TMEM271, LOC119717909, LOC119715439, LOC119716370, LOC113840447, CDT1, LOC113842721, NDST4, WDR88, CSK, LOC119715853, CRYBB1, STX11, LOC113843889, LOC119715808, LOC113843711, STK32A, LOC106016195, CDCA3, LOC119715071, LOC110353184, ISLR2, PPP2R2C, LOC119717681, SELPLG, DOCK8, VSIG10, KCNAB2, LOC101802354, LOC101803633, LOC101791013, IL12RB1, LOC113842864, FOXI1, SLA2, LOC101804544, LOC119714741, PPP1R16B, LOC110352159, LOC101797094, LOC113841604, PRKCB, LOC101803899, CD83, LOC101802873, COL26A1, LOC119714593, LOC101797884, LOC119717843, LOC113845438, HIVEP3, LOC113845344, NTN1, PRPS2, LOC119715491, WNT1, CCNB3, LOC101802774, MSX1, ELMO1, TNIP3, LOC106016663, LOC119715062, CCL28, LOC119718695, PCARE, IRF8, LOC119715244, LOC113841478, TMEM61, P2RX5, LOC101804790, LOC119715912, NCF2, LOC119713184, LOC101804337, LOC101791611, GINS1, KCNQ4, CFAP206, NRROS, FUT7, LOC101795263, ALKBH2, GRK6, TENT5C, STXBP5L, LOC113841163, SOWAHD, MCM3, LOC101796364, DOK1, VOPP1, LOC110352533, GNG2, LOC119718013, LEF1, MPEG1, SPAG5, RGS9, NRIP3, MYBL2, PTK2B, RUNX2, TMPRSS6, LOC113841634, LOC119718679, LOC101802747, UNG, LOC119715851, IFI30, PIF1, MYBPC3, LOC119718708, TMEFF1, LOC101801606, SLC5A8, LOC119713921, LOC119716630, VCAM1, LOC119713851, LOC101790950, TMEM71, SH3TC2, LOC113840481, LOC113839830, LOC101789439, ANKLE1, MAD2L1, FAM167B, LOC101797250, SLC9A7, LOC101798385, LOC101801508, LOC101800206, LOC101790981, LOC113839785, LOC101799094, NEK2, BHLHE22, LOC113845703, LOC113840583, LOC113843352, CSRP1, LOC101789604, LOC119716307, MIS12, LIPI, LOC113843744, TLR2, LOC119714949, LOC113843526, LOC101804573, DEPDC1B, HVCN1, JCHAIN, ARHGDIB, LOC106018871, CNTFR, TOP2A, RAB37, LOC101799932, LOC119716322, LOC113839607, LYN, LOC101790280, CCKAR, LOC101792885, RFTN1, LOC119713786, LOC113845717, CDCA8, TMEM273, LOC119714521, CCL19, LOC101798906, LOC110351953, STAT4, ERICH6, RAD51, LOC119715854, IL2RB, POLH, KIF14, LOC119714816, LOC101798418, LOC119717548, LOC119718068, LOC113840606, GNB3, LOC119715242, LOC101803950, BID, TRAF5, RHEX, LOC113840524, LOC101804875, FANCE, TOX, CENPO, DEPDC1, GCNT3, LOC101791991, NDC80, RHOF, MCM6, RGS14, LOC113839706, LOC119718395, UNC13B, LOC119714137, GRK3, LIMK1, LOC119714135, LOC113842374, RACGAP1, LYPD6B, LRRC26, PARP8, LOC110351750, LOC119715121, LOC106017632, NCF1, VWA5B1, P2RY13, SUSD3, LOC101804340, LRRC8B, LOC119715166, IL23R, LOC113843518, LOC106015872, LOC119713039, PREX1, UHRF1, LOC101797872, TDRD6, EVL, TBC1D9, SNAP91, NXPE3, LOC101804463, LOC101791319, LOC106020629, ANO9, LOC113839872, LOC101800880, GPR65, PITPNM2, FAM72A, LOC101804125, ANOS1, TRIM66, HTR7, LIG1, LOC113845030, NCAPG2, LOC119717226, HYLS1, LOC101795725, LOC113840953, LOC113842971, LOC113841633, KIF18A, SPI1, LOC119714150, LOC119715848, C3, LOC101797257, LOC101796665, B3GNT4, RGS6, LOC119713029, PRR15L, CCNA2, HASPIN, KLHDC7A, FOXM1, LOC106018996, LPAR3, CXXC4, KMO, CD74, LOC119714866, PTPRE, RASSF2, LOC119717101, LOC113845767, CHST1, LOC106015647, PHETA1, HAPLN1, CDC45, MCM8, LOC101800410, RSPO2, CYTH4, E2F2, PAG1, AMDHD1, NCOA7, LOC119714997, FAIM2, LOC113841322, APLN, KIF11, PRR11, FIGNL1, LOC101805205, TNFRSF9, TRIM71, LOC119715234, LOC106016976, LOC110351762, FAM78A, LOC101793373, LOC101794174, LOC119713005, KIF2C, NANOS1, NKAIN4, TPX2, PARVG, TFEC, BLNK, LOC119714984, HHEX, ZDHHC18, LOC119714744, BANK1, PTPN22, LOC101794002, ORC1, AURKA, LOC113841603, TTK, IL10RA, BUB1B, KIFC1, LOC119716364, MIS18BP1, LOC119716678, SHMT1, LOC113841845, OIP5, LOC101798714, RFFL, LOC113841777, GPER1, CTSS, LOC101803519, MATK, ZC3H12D, FANCB, RGS1, WIPF1, LOC101802373, RHOBTB1, HCK, TTLL10, MASTL, DLGAP5, DTL, KNSTRN, INPP5B, LOC101796470, LOC119716486, BARD1, RILPL2, LOC101790356, LOC113842037, CEP55, DERL3, KCNA4, IL18R1, IQGAP3, CD7, FGD3, CENPI, LOC101800605, LOC119714139, LOC119717467, LOC119717845, LOC119714870, GRN, KPNA2, LOC119713081, CDK1, LOC119713652, NFKB2, LOC101803634, LOC119714581, LOC101798073, LOC119715301, PPFIA4, SMC2, GDF1, LOC101794526, PTPRJ, LOC119714804, LOC106017661, LZTS3, ERICH3, XYLT1, CNR2, SPRY4, SLC9A5, LOC101802472, TMEM268, SNX20, GPR35, LOC101804808, SPC25, CDC20, PRC1, ECT2, LOC119713063, UNC79, CIP2A, NCAPH, PLEKHA2, RUBCN, CFAP251, AKNA, LOC101795552, KIF4A, INPP5D, SKA1, FLI1, IL16, LOC119712979, LOC119716807, LOC113845608, LY96, LOC106015834, CEP135, GINS2, ARPC1B, MCOLN2, LOC113841909, CENPL, CX3CR1, NOS2, LOC119715946, NCAPD3, LOC119714408, TNFRSF18, LOC113840297, E2F8, LOC101803728, LBH, DGKZ, LOC101797323, LOC101803870, DENND2D, IPCEF1, ASL, LOC101789516, ARHGAP27, CENPN, LOC119713707, JAKMIP2, LOC119715374, LOC113840340, LOC110353222, LOC101800944, CORO1C
Downregulated DEGs	532	*LOC101796123, LOC101803316, LOC119713823, LOC119713220, MMD2, LOC101791608, LOC119713219, LOC119715297, LOC119716953, MDGA1, LOC101791401, LOC101794508, LOC106018567, LOC119713221, LOC101798492, LOC113843567, KYNU, CACNB4, GPD1, LOC119716546, LOC113844155, LOC101794396, LOC113839837, PAPPA2, METTL21C, DUSP29, ARX, SIX3, LOC101793478, C7H21orf58, LMOD3, MYL2, C2H8orf34, LOC106015356, IRX1, LOC119715992, LOC106015261, LOC113840240, LOC101794749, LOC101796474, LOC119715271, IRX2, LOC101802165, LOC101802071, LOC113843334, TAFA2, IP6K3, MYL1, LOC106020235, FHL3, ACTA1, ASB10, LOC110351189, LOC119713167, PENK, LOC106019045, TNNI2, PTX3, LOC113840724, GC, LOC110351497, LOC113842688, NR4A1, MB, LOC113842792, LOC101790909, TMEM233, SLC30A2, TRIM63, TNNT3, DRGX, ASB4, LOC101793992, RAMP1, SLC25A4, USP2, LOC110353953, MYBPH, B3GALT9, ITGB1BP2, LOC110353218, LOC119717600, MYOM2, LOC101802277, LOC101793880, MYOZ1, ASB12, LOC119718438, LOC110352722, LOC113840723, OBSL1, KCNV2, TMEM229B, PNMT, KCNIP1, CRH, LOC106016943, LOC113841418, LOC119717776, MYLK3, LOC106017354, LOC113841424, REEP6, C6H10orf71, LOC101798499, LOC106014873, LOC110352096, LOC101795465, LOC113840188, FHL2, RFESD, LOC101792626, ENDOU, LOC119717850, TMEM74, LOC119717540, FSD2, PPIL6, ADAMTS20, LOC101799421, LOC101794131, LOC119716429, LBX1, RPRM, LOC119712999, LOC106015797, NT5C1A, SLITRK3, RANBP3L, LOC119718360, LOC101804261, CPT1B, BMP5, LOC110351363, CRYAB, unassigned_gene_11, TNNI1, SLC8A3, LOC119715547, LOC110351399, ERBB4, RAPGEF4, PPP1R1A, SLC7A2, PDK4, LOC106018389, SMTNL2, LOC101797129, LRRC38, RPP25, CYTL1, LOC119716592, PGM1, STAC3, LOC119714295, SLC41A3, LOC110351904, LOC101791922, DNAJB13, CKMT2, RNF207, RGS16, LOC119717520, LOC110352854, LOC119715738, LOC119713328, LOC101798290, DBX2, BEST3, CORO6, ATP2A2, FKBP1B, LOC113843291, MYLK4, LOC101795494, ARPP21, MMP17, CACNA2D3, LOC113839608, LOC101800711, LOC113843972, LOC101796443, CPB1, EEF1A2, KY, LOC119717603, PLN, LOC110354480, CLCN1, LOC113842743, LACTBL1, LOC101796243, KBTBD13, LOC113843713, KLHL31, SMOC1, LOC113840410, CHAD, LOC106017289, LOC119718232, LOC106015458, LOC113843555, ACE, SGCA, LOC119717734, TXLNB, LOC101800520, PRKAB2, ARMC12, AGTR1, NR4A3, HSPB7, KLHL33, SNCB, unassigned_gene_13, RAPSN, LOC119713489, LDB3, LOC101792918, LOC119714566, CTNNA3, IRS2, TNFAIP6, TPM2, TMEM52, MATN3, IGF1, LDHD, unassigned_gene_16, JPH1, LOC101797569, IGDCC4, LOC119715675, NRN1, PPDPF, LOC110354152, LOC101790517, ANO8, LOC113844859, RGS11, HOGA1, MCUB, LOC113844150, TMEM132E, RHAG, LOC110351420, KCND2, MAP3K7CL, POPDC2, SLN, CUTA, LOC119714927, PDE4B, TPM3, KCNH7, SPTB, LOC101800414, NMNAT3, LOC119718096, LOC119717851, LOC101799407, COL28A1, SHISA3, MEOX1, TMEM38A, DAB1, FGF12, CAPN3, LOC101789609, MLF1, SMPX, LRRC20, COL22A1, unassigned_gene_1, BBOX1, LOC101794644, GAL, HOXC8, LOC101802901, unassigned_gene_12, LOC101792412, ACACB, LOC119718250, LOC101794949, SPEG, SEMA6B, LOC101798468, CSDC2, ADAM11, LRRC56, LRP1B, LOC101804173, RBP7, TMOD1, PDE4C, MCRIP2, AJM1, NGB, MME, LOC110354585, LOC119713290, LOC101803486, LOC101800026, DDX60, COQ8A, LOC101790713, FKBP5, KEL, GRB14, CARNS1, RASL12, LOC106014707, VTN, HTR3A, TMEM247, LOC106014639, NDRG4, G0S2, LOC110353784, PCBP4, SIK2, STC2, COL25A1, LOC113841470, LOC113844903, CAP2, LOC106017813, FSD1L, LOC119713729, KCNJ2, CLCN2, LOC101801043, FAM181B, NOX3, PHKA1, COL8A1, LOC113845026, S100A1, CRHR2, BOLL, LOC101803098, LOC119717444, ESRRB, NRXN1, LOC119714519, GAMT, DUSP28, LOC119714811, OPRM1, LOC119713150, HTR1A, LOC113844715, REEP1, LOC106016021, ANK1, DNAI1, FKBP3, SIK1, LOC101793529, DMTN, COQ10A, OXTR, ACADL, LOC113840744, BIN1, LOC101796567, FHL1, SOX6, ACYP2, C5H14orf180, FBXL22, EPDR1, LOC119715213, SEZ6L, LOC110351385, PPP1R27, SLC38A3, PABPC4, LOC119713253, LOC106019837, RBFOX2, TMOD4, LYSMD4, TM6SF1, TSKU, TEAD4, LOC101802744, LOC101794782, LOC101793418, LOC119714227, ARMC3, ENTPD2, PDLIM5, RAPGEF5, CEP85, LOC119717819, LOC101795226, DES, LUZP2, MAPK8IP1, FGF14, LOC113844783, LOC101802576, LOC101791103,*

	FILIP1, KCNH2, GTF2A1L, KCNIP4, LOC119714070, LOC101797243, EIF4E3, RALY, LOC101803344, TPM1, SLC6A8, LOC110351260, LOC101797856, NAAA, FHOD3, SESN2, LOC119717849, GPC3, UGP2, SLC25A12, TOR3A, SLC25A33, PCP4, MYOZ3, ATP1B4, CUBN, PPP1R3C, SLC16A7, LOC113843748, FSHR, RAB26, STMN4, JPH2, ADAMTS5, SH3BP5, PLXNA4, LOC113845281, LOC101791292, LOC101802407, LOC101797088, GPAM, LOC101801369, ZBTB16, SNTA1, LOC119714921, PKDCC, LOC113840930, LOC113845000, MYADML2, NT5C3A, MSANTD1, TMEM109, LOC119718720, MSH4, CLTCL1, LOC119713575, ALPK2, ANKS1B, RERGL, AKR1D1, TAL1, NSUN7, COL4A4, IL6R, LOC101797420, COL15A1, SIX1, ACO2, CACNA2D1, CDR2L, LOC119713225, RASGEF1B, PLCXD2, LOC113844890, NSMF, GLUL, PLCL1, BRINP3, LOC119717601, ZNF106, INSYN2A, LOC119716632, LOC101798394, LOC101793527, LSMEM1, PKNOX2, BAG2, LOC119714223, PSPH, LOC113839684, INSYN1, LOC101796773, FAM78B, KHDRBS3, SLC27A1, LOC119717941, CCBE1, LRRC39, TFPI, OLFML1, SGCD, CD36, CISD3, ME3, SLC19A2, LOC113843522, LOC119718147, IRS1, ADAMTS1, GPRC5B, ANGPTL1, GPR85, LOC119716120, LOC106016919, RNF180, LAMA2, LOC106016986, MCF2, LOC119714920, LPL, LOC119716125, COQ10B, LOC106015025, ADSS1, LOC101799835, CFAP70, GPX3, HSPB8

Supplement 5. GO enrichment analysis of DEGs in ID vs NID.

ONTOLOGY	ID	Description	GeneRatio	count	BgRatio	pvalue	p.adjust	qvalue	Count
BP	GO:0002376	immune system process	14/43	14	44/559	6.67E-07	0.00011339	9.13E-05	14
BP	GO:0006955	immune response	13/43	13	41/559	1.99E-06	0.000156383	0.000125881	13
BP	GO:0006954	inflammatory response	8/43	8	15/559	2.76E-06	0.000156383	0.000125881	8
BP	GO:0006952	defense response	10/43	10	27/559	8.14E-06	0.000346084	0.000278582	10
BP	GO:0002684	positive regulation of immune system process	7/43	7	13/559	1.19E-05	0.000405274	0.000326227	7
BP	GO:0002833	positive regulation of response to biotic stimulus	6/43	6	11/559	5.08E-05	0.000863776	0.0006953	6
BP	GO:0031349	positive regulation of defense response	6/43	6	11/559	5.08E-05	0.000863776	0.0006953	6
BP	GO:0032103	positive regulation of response to external stimulus	6/43	6	11/559	5.08E-05	0.000863776	0.0006953	6
BP	GO:0045088	regulation of innate immune response	6/43	6	11/559	5.08E-05	0.000863776	0.0006953	6
BP	GO:0045089	positive regulation of innate immune response	6/43	6	11/559	5.08E-05	0.000863776	0.0006953	6
BP	GO:0002682	regulation of immune system process	7/43	7	16/559	6.66E-05	0.001017718	0.000819216	7
BP	GO:0002831	regulation of response to biotic stimulus	6/43	6	12/559	9.58E-05	0.001017718	0.000819216	6
BP	GO:0031347	regulation of defense response	6/43	6	12/559	9.58E-05	0.001017718	0.000819216	6
BP	GO:0032101	regulation of response to external stimulus	6/43	6	12/559	9.58E-05	0.001017718	0.000819216	6
BP	GO:0050778	positive regulation of immune response	6/43	6	12/559	9.58E-05	0.001017718	0.000819216	6
BP	GO:0080134	regulation of response to stress	6/43	6	12/559	9.58E-05	0.001017718	0.000819216	6
MF	GO:0005125	cytokine activity	7/56	7	17/743	0.000102601	0.010157465	0.009288059	7
BP	GO:0009605	response to external stimulus	9/43	9	29/559	0.000129091	0.001290909	0.001039122	9
BP	GO:0048518	positive regulation of biological process	10/43	10	36/559	0.000146124	0.001380065	0.001110888	10
BP	GO:0045087	innate immune response	7/43	7	18/559	0.00016455	0.001425194	0.001147215	7
BP	GO:0050776	regulation of immune response	6/43	6	13/559	0.00016767	0.001425194	0.001147215	6
CC	GO:0005615	extracellular space	10/54	10	39/728	0.000261862	0.010474483	0.010474483	10
BP	GO:0048584	positive regulation of response to stimulus	6/43	6	14/559	0.000276566	0.002238871	0.001802188	6
BP	GO:0006950	response to stress	11/43	11	47/559	0.000349755	0.002371699	0.001909108	11
BP	GO:0002218	activation of innate immune response	5/43	5	10/559	0.000404584	0.002371699	0.001909108	5
BP	GO:0002221	pattern recognition receptor signaling pathway	5/43	5	10/559	0.000404584	0.002371699	0.001909108	5
BP	GO:0002224	toll-like receptor signaling pathway	5/43	5	10/559	0.000404584	0.002371699	0.001909108	5
BP	GO:0002253	activation of immune response	5/43	5	10/559	0.000404584	0.002371699	0.001909108	5
BP	GO:0002757	immune response-activating signaling pathway	5/43	5	10/559	0.000404584	0.002371699	0.001909108	5
BP	GO:0002758	innate immune response-activating signaling pathway	5/43	5	10/559	0.000404584	0.002371699	0.001909108	5
BP	GO:0002764	immune response-regulating signaling pathway	5/43	5	10/559	0.000404584	0.002371699	0.001909108	5
BP	GO:0009607	response to biotic stimulus	7/43	7	21/559	0.00050346	0.002517299	0.002026309	7
BP	GO:0043207	response to external biotic stimulus	7/43	7	21/559	0.00050346	0.002517299	0.002026309	7
