# Supplementary material for: Integrated transcriptomic and metabolomic analyses reveal host–metabolite interactions underlying cloacal inflammation in laying Pekin ducks
Source: Anim Biosci. 2026 Apr 16;39(10):260208. doi: 10.5713/ab.260208 (PMC13662949; doi:10.5713/ab.260208)
Supplement: Supplementary file 8 [file ab-260208-Supplementary-5.pdf]

	FILIP1, KCNH2, GTF2A1L, KCNIP4, LOC119714070, LOC101797243, EIF4E3, RALY, LOC101803344, TPM1, SLC6A8, LOC110351260, LOC101797856, NAAA, FHOD3, SESN2, LOC119717849, GPC3, UGP2, SLC25A12, TOR3A, SLC25A33, PCP4, MYOZ3, ATP1B4, CUBN, PPP1R3C, SLC16A7, LOC113843748, FSHR, RAB26, STMN4, JPH2, ADAMTS5, SH3BP5, PLXNA4, LOC113845281, LOC101791292, LOC101802407, LOC101797088, GPAM, LOC101801369, ZBTB16, SNTA1, LOC119714921, PKDCC, LOC113840930, LOC113845000, MYADML2, NT5C3A, MSANTD1, TMEM109, LOC119718720, MSH4, CLTCL1, LOC119713575, ALPK2, ANKS1B, RERGL, AKR1D1, TAL1, NSUN7, COL4A4, IL6R, LOC101797420, COL15A1, SIX1, ACO2, CACNA2D1, CDR2L, LOC119713225, RASGEF1B, PLCXD2, LOC113844890, NSMF, GLUL, PLCL1, BRINP3, LOC119717601, ZNF106, INSYN2A, LOC119716632, LOC101798394, LOC101793527, LSMEM1, PKNOX2, BAG2, LOC119714223, PSPH, LOC113839684, INSYN1, LOC101796773, FAM78B, KHDRBS3, SLC27A1, LOC119717941, CCBE1, LRRC39, TFPI, OLFML1, SGCD, CD36, CISD3, ME3, SLC19A2, LOC113843522, LOC119718147, IRS1, ADAMTS1, GPRC5B, ANGPTL1, GPR85, LOC119716120, LOC106016919, RNF180, LAMA2, LOC106016986, MCF2, LOC119714920, LPL, LOC119716125, COQ10B, LOC106015025, ADSS1, LOC101799835, CFAP70, GPX3, HSPB8

Supplement 5. GO enrichment analysis of DEGs in ID vs NID.

ONTOLOGY	ID	Description	GeneRatio	count	BgRatio	pvalue	p.adjust	qvalue	Count
BP	GO:0002376	immune system process	14/43	14	44/559	6.67E-07	0.00011339	9.13E-05	14
BP	GO:0006955	immune response	13/43	13	41/559	1.99E-06	0.000156383	0.000125881	13
BP	GO:0006954	inflammatory response	8/43	8	15/559	2.76E-06	0.000156383	0.000125881	8
BP	GO:0006952	defense response	10/43	10	27/559	8.14E-06	0.000346084	0.000278582	10
BP	GO:0002684	positive regulation of immune system process	7/43	7	13/559	1.19E-05	0.000405274	0.000326227	7
BP	GO:0002833	positive regulation of response to biotic stimulus	6/43	6	11/559	5.08E-05	0.000863776	0.0006953	6
BP	GO:0031349	positive regulation of defense response	6/43	6	11/559	5.08E-05	0.000863776	0.0006953	6
BP	GO:0032103	positive regulation of response to external stimulus	6/43	6	11/559	5.08E-05	0.000863776	0.0006953	6
BP	GO:0045088	regulation of innate immune response	6/43	6	11/559	5.08E-05	0.000863776	0.0006953	6
BP	GO:0045089	positive regulation of innate immune response	6/43	6	11/559	5.08E-05	0.000863776	0.0006953	6
BP	GO:0002682	regulation of immune system process	7/43	7	16/559	6.66E-05	0.001017718	0.000819216	7
BP	GO:0002831	regulation of response to biotic stimulus	6/43	6	12/559	9.58E-05	0.001017718	0.000819216	6
BP	GO:0031347	regulation of defense response	6/43	6	12/559	9.58E-05	0.001017718	0.000819216	6
BP	GO:0032101	regulation of response to external stimulus	6/43	6	12/559	9.58E-05	0.001017718	0.000819216	6
BP	GO:0050778	positive regulation of immune response	6/43	6	12/559	9.58E-05	0.001017718	0.000819216	6
BP	GO:0080134	regulation of response to stress	6/43	6	12/559	9.58E-05	0.001017718	0.000819216	6
MF	GO:0005125	cytokine activity	7/56	7	17/743	0.000102601	0.010157465	0.009288059	7
BP	GO:0009605	response to external stimulus	9/43	9	29/559	0.000129091	0.001290909	0.001039122	9
BP	GO:0048518	positive regulation of biological process	10/43	10	36/559	0.000146124	0.001380065	0.001110888	10
BP	GO:0045087	innate immune response	7/43	7	18/559	0.00016455	0.001425194	0.001147215	7
BP	GO:0050776	regulation of immune response	6/43	6	13/559	0.00016767	0.001425194	0.001147215	6
CC	GO:0005615	extracellular space	10/54	10	39/728	0.000261862	0.010474483	0.010474483	10
BP	GO:0048584	positive regulation of response to stimulus	6/43	6	14/559	0.000276566	0.002238871	0.001802188	6
BP	GO:0006950	response to stress	11/43	11	47/559	0.000349755	0.002371699	0.001909108	11
BP	GO:0002218	activation of innate immune response	5/43	5	10/559	0.000404584	0.002371699	0.001909108	5
BP	GO:0002221	pattern recognition receptor signaling pathway	5/43	5	10/559	0.000404584	0.002371699	0.001909108	5
BP	GO:0002224	toll-like receptor signaling pathway	5/43	5	10/559	0.000404584	0.002371699	0.001909108	5
BP	GO:0002253	activation of immune response	5/43	5	10/559	0.000404584	0.002371699	0.001909108	5
BP	GO:0002757	immune response-activating signaling pathway	5/43	5	10/559	0.000404584	0.002371699	0.001909108	5
BP	GO:0002758	innate immune response-activating signaling pathway	5/43	5	10/559	0.000404584	0.002371699	0.001909108	5
BP	GO:0002764	immune response-regulating signaling pathway	5/43	5	10/559	0.000404584	0.002371699	0.001909108	5
BP	GO:0009607	response to biotic stimulus	7/43	7	21/559	0.00050346	0.002517299	0.002026309	7
BP	GO:0043207	response to external biotic stimulus	7/43	7	21/559	0.00050346	0.002517299	0.002026309	7

BP	GO:0044419	biological process involved in interspecies interaction between organisms	7/43	7	21/559	0.00050346	0.002517299	0.002026309	7
BP	GO:0051707	response to other organism	7/43	7	21/559	0.00050346	0.002517299	0.002026309	7
BP	GO:0098542	defense response to other organism	7/43	7	21/559	0.00050346	0.002517299	0.002026309	7
BP	GO:0050896	response to stimulus	23/43	23	166/559	0.00058258	0.002829674	0.002277757	23
MF	GO:0005126	cytokine receptor binding	6/56	6	18/743	0.001283561	0.057288453	0.052384976	6
BP	GO:0048583	regulation of response to stimulus	8/43	8	34/559	0.002543958	0.012013134	0.009670015	8
MF	GO:0030545	signaling receptor regulator activity	9/56	9	43/743	0.003078417	0.057288453	0.052384976	9
MF	GO:0030546	signaling receptor activator activity	9/56	9	43/743	0.003078417	0.057288453	0.052384976	9
MF	GO:0048018	receptor ligand activity	9/56	9	43/743	0.003078417	0.057288453	0.052384976	9
MF	GO:0140677	molecular function activator activity	10/56	10	52/743	0.003472027	0.057288453	0.052384976	10
CC	GO:0005576	extracellular region	14/54	14	97/728	0.007283678	0.145673565	0.145673565	14
BP	GO:0008283	cell population proliferation	4/43	4	12/559	0.009571137	0.043975494	0.03539823	4
MF	GO:0005102	signaling receptor binding	10/56	10	60/743	0.010208477	0.129202318	0.118143534	10
MF	GO:0098772	molecular function regulator activity	13/56	13	89/743	0.010440591	0.129202318	0.118143534	13
MF	GO:0005515	protein binding	15/56	15	116/743	0.018033961	0.182145296	0.166554976	15
MF	GO:0004888	transmembrane signaling receptor activity	12/56	12	85/743	0.018398515	0.182145296	0.166554976	12
BP	GO:0051716	cellular response to stimulus	14/43	14	113/559	0.033320551	0.149065624	0.119990904	14
BP	GO:0007165	signal transduction	13/43	13	103/559	0.035577811	0.152230507	0.122538488	13
BP	GO:0007154	cell communication	14/43	14	114/559	0.035818943	0.152230507	0.122538488	14
MF	GO:0038023	signaling receptor activity	12/56	12	93/743	0.035879331	0.296004482	0.270668638	12
MF	GO:0060089	molecular transducer activity	12/56	12	93/743	0.035879331	0.296004482	0.270668638	12
MF	GO:0016791	phosphatase activity	3/56	3	11/743	0.043386766	0.330406907	0.302126464	3

Supplement 6. GO enrichment analysis of DEGs in ID vs HC.

ONTOLOGY	ID	Description	GeneRatio	count	BgRatio	pvalue	p.adjust	qvalue	Count
BP	GO:0006955	immune response	8/27	8	41/559	0.000330827	0.00987221	0.007326593	8
BP	GO:0006954	inflammatory response	5/27	5	15/559	0.000387252	0.00987221	0.007326593	5
BP	GO:0002376	immune system process	8/27	8	44/559	0.000556738	0.00987221	0.007326593	8
BP	GO:0002218	activation of innate immune response	4/27	4	10/559	0.000747944	0.00987221	0.007326593	4
BP	GO:0002221	pattern recognition receptor signaling pathway	4/27	4	10/559	0.000747944	0.00987221	0.007326593	4
BP	GO:0002224	toll-like receptor signaling pathway	4/27	4	10/559	0.000747944	0.00987221	0.007326593	4
BP	GO:0002253	activation of immune response	4/27	4	10/559	0.000747944	0.00987221	0.007326593	4
BP	GO:0002757	immune response-activating signaling pathway	4/27	4	10/559	0.000747944	0.00987221	0.007326593	4
BP	GO:0002758	innate immune response-activating signaling pathway	4/27	4	10/559	0.000747944	0.00987221	0.007326593	4
BP	GO:0002764	immune response-regulating signaling pathway	4/27	4	10/559	0.000747944	0.00987221	0.007326593	4
BP	GO:0050896	response to stimulus	16/27	16	166/559	0.001016714	0.00987221	0.007326593	16
BP	GO:0002833	positive regulation of response to biotic stimulus	4/27	4	11/559	0.001136369	0.00987221	0.007326593	4
BP	GO:0031349	positive regulation of defense response	4/27	4	11/559	0.001136369	0.00987221	0.007326593	4
BP	GO:0032103	positive regulation of response to external stimulus	4/27	4	11/559	0.001136369	0.00987221	0.007326593	4
BP	GO:0045088	regulation of innate immune response	4/27	4	11/559	0.001136369	0.00987221	0.007326593	4
