# Supplementary material for: Integrated transcriptomic and metabolomic analyses reveal host–metabolite interactions underlying cloacal inflammation in laying Pekin ducks
Source: Anim Biosci. 2026 Apr 16;39(10):260208. doi: 10.5713/ab.260208 (PMC13662949; doi:10.5713/ab.260208)
Supplement: Supplementary file 9 [file ab-260208-Supplementary-6.pdf]

BP	GO:0044419	biological process involved in interspecies interaction between organisms	7/43	7	21/559	0.00050346	0.002517299	0.002026309	7
BP	GO:0051707	response to other organism	7/43	7	21/559	0.00050346	0.002517299	0.002026309	7
BP	GO:0098542	defense response to other organism	7/43	7	21/559	0.00050346	0.002517299	0.002026309	7
BP	GO:0050896	response to stimulus	23/43	23	166/559	0.00058258	0.002829674	0.002277757	23
MF	GO:0005126	cytokine receptor binding	6/56	6	18/743	0.001283561	0.057288453	0.052384976	6
BP	GO:0048583	regulation of response to stimulus	8/43	8	34/559	0.002543958	0.012013134	0.009670015	8
MF	GO:0030545	signaling receptor regulator activity	9/56	9	43/743	0.003078417	0.057288453	0.052384976	9
MF	GO:0030546	signaling receptor activator activity	9/56	9	43/743	0.003078417	0.057288453	0.052384976	9
MF	GO:0048018	receptor ligand activity	9/56	9	43/743	0.003078417	0.057288453	0.052384976	9
MF	GO:0140677	molecular function activator activity	10/56	10	52/743	0.003472027	0.057288453	0.052384976	10
CC	GO:0005576	extracellular region	14/54	14	97/728	0.007283678	0.145673565	0.145673565	14
BP	GO:0008283	cell population proliferation	4/43	4	12/559	0.009571137	0.043975494	0.03539823	4
MF	GO:0005102	signaling receptor binding	10/56	10	60/743	0.010208477	0.129202318	0.118143534	10
MF	GO:0098772	molecular function regulator activity	13/56	13	89/743	0.010440591	0.129202318	0.118143534	13
MF	GO:0005515	protein binding	15/56	15	116/743	0.018033961	0.182145296	0.166554976	15
MF	GO:0004888	transmembrane signaling receptor activity	12/56	12	85/743	0.018398515	0.182145296	0.166554976	12
BP	GO:0051716	cellular response to stimulus	14/43	14	113/559	0.033320551	0.149065624	0.119990904	14
BP	GO:0007165	signal transduction	13/43	13	103/559	0.035577811	0.152230507	0.122538488	13
BP	GO:0007154	cell communication	14/43	14	114/559	0.035818943	0.152230507	0.122538488	14
MF	GO:0038023	signaling receptor activity	12/56	12	93/743	0.035879331	0.296004482	0.270668638	12
MF	GO:0060089	molecular transducer activity	12/56	12	93/743	0.035879331	0.296004482	0.270668638	12
MF	GO:0016791	phosphatase activity	3/56	3	11/743	0.043386766	0.330406907	0.302126464	3

Supplement 6. GO enrichment analysis of DEGs in ID vs HC.

ONTOLOGY	ID	Description	GeneRatio	count	BgRatio	pvalue	p.adjust	qvalue	Count
BP	GO:0006955	immune response	8/27	8	41/559	0.000330827	0.00987221	0.007326593	8
BP	GO:0006954	inflammatory response	5/27	5	15/559	0.000387252	0.00987221	0.007326593	5
BP	GO:0002376	immune system process	8/27	8	44/559	0.000556738	0.00987221	0.007326593	8
BP	GO:0002218	activation of innate immune response	4/27	4	10/559	0.000747944	0.00987221	0.007326593	4
BP	GO:0002221	pattern recognition receptor signaling pathway	4/27	4	10/559	0.000747944	0.00987221	0.007326593	4
BP	GO:0002224	toll-like receptor signaling pathway	4/27	4	10/559	0.000747944	0.00987221	0.007326593	4
BP	GO:0002253	activation of immune response	4/27	4	10/559	0.000747944	0.00987221	0.007326593	4
BP	GO:0002757	immune response-activating signaling pathway	4/27	4	10/559	0.000747944	0.00987221	0.007326593	4
BP	GO:0002758	innate immune response-activating signaling pathway	4/27	4	10/559	0.000747944	0.00987221	0.007326593	4
BP	GO:0002764	immune response-regulating signaling pathway	4/27	4	10/559	0.000747944	0.00987221	0.007326593	4
BP	GO:0050896	response to stimulus	16/27	16	166/559	0.001016714	0.00987221	0.007326593	16
BP	GO:0002833	positive regulation of response to biotic stimulus	4/27	4	11/559	0.001136369	0.00987221	0.007326593	4
BP	GO:0031349	positive regulation of defense response	4/27	4	11/559	0.001136369	0.00987221	0.007326593	4
BP	GO:0032103	positive regulation of response to external stimulus	4/27	4	11/559	0.001136369	0.00987221	0.007326593	4
BP	GO:0045088	regulation of innate immune response	4/27	4	11/559	0.001136369	0.00987221	0.007326593	4

BP	GO:0045089	positive regulation of innate immune response	4/27	4	11/559	0.001136369	0.00987221	0.007326593	4
BP	GO:0009605	response to external stimulus	6/27	6	29/559	0.001581064	0.010412609	0.007727646	6
BP	GO:0002831	regulation of response to biotic stimulus	4/27	4	12/559	0.001648039	0.010412609	0.007727646	4
BP	GO:0031347	regulation of defense response	4/27	4	12/559	0.001648039	0.010412609	0.007727646	4
BP	GO:0032101	regulation of response to external stimulus	4/27	4	12/559	0.001648039	0.010412609	0.007727646	4
BP	GO:0050778	positive regulation of immune response	4/27	4	12/559	0.001648039	0.010412609	0.007727646	4
BP	GO:0080134	regulation of response to stress	4/27	4	12/559	0.001648039	0.010412609	0.007727646	4
BP	GO:0002684	positive regulation of immune system process	4/27	4	13/559	0.002301579	0.013329977	0.009892751	4
BP	GO:0050776	regulation of immune response	4/27	4	13/559	0.002301579	0.013329977	0.009892751	4
BP	GO:0048584	positive regulation of response to stimulus	4/27	4	14/559	0.003115392	0.017321578	0.01285509	4
BP	GO:0048518	positive regulation of biological process	6/27	6	36/559	0.005117603	0.02725984	0.020230703	6
BP	GO:0002682	regulation of immune system process	4/27	4	16/559	0.005295077	0.02725984	0.020230703	4
BP	GO:0006952	defense response	5/27	5	27/559	0.006930558	0.034405271	0.025533636	5
BP	GO:0045087	innate immune response	4/27	4	18/559	0.008322482	0.039890518	0.029604474	4
BP	GO:0009607	response to biotic stimulus	4/27	4	21/559	0.014713373	0.060151732	0.044641194	4
BP	GO:0043207	response to external biotic stimulus	4/27	4	21/559	0.014713373	0.060151732	0.044641194	4
BP	GO:0044419	biological process involved in interspecies interaction between organisms	4/27	4	21/559	0.014713373	0.060151732	0.044641194	4
BP	GO:0051707	response to other organism	4/27	4	21/559	0.014713373	0.060151732	0.044641194	4
BP	GO:0098542	defense response to other organism	4/27	4	21/559	0.014713373	0.060151732	0.044641194	4
BP	GO:0006950	response to stress	6/27	6	47/559	0.019412492	0.077095324	0.057215765	6
BP	GO:0023052	signaling	10/27	10	109/559	0.022825746	0.088132743	0.06540711	10
BP	GO:0051716	cellular response to stimulus	10/27	10	113/559	0.029140754	0.107819357	0.080017395	10
BP	GO:0007154	cell communication	10/27	10	114/559	0.030905187	0.107819357	0.080017395	10
BP	GO:0007264	small GTPase mediated signal transduction	3/27	3	15/559	0.031027153	0.107819357	0.080017395	3
BP	GO:0042221	response to chemical	3/27	3	15/559	0.031027153	0.107819357	0.080017395	3
BP	GO:0007165	signal transduction	9/27	9	103/559	0.042974363	0.145693573	0.108125484	9
CC	GO:0005615	extracellular space	9/37	9	39/728	5.92E-05	0.001894173	0.00186925	9
CC	GO:0005576	extracellular region	13/37	13	97/728	0.000437649	0.007002387	0.006910251	13
MF	GO:0005125	cytokine activity	4/41	4	17/743	0.011272885	0.267022925	0.25027383	4
MF	GO:0005126	cytokine receptor binding	4/41	4	18/743	0.013918955	0.267022925	0.25027383	4
MF	GO:0038023	signaling receptor activity	10/41	10	93/743	0.023413316	0.267022925	0.25027383	10
MF	GO:0060089	molecular transducer activity	10/41	10	93/743	0.023413316	0.267022925	0.25027383	10
MF	GO:0030545	signaling receptor regulator activity	6/41	6	43/743	0.025604938	0.267022925	0.25027383	6
MF	GO:0030546	signaling receptor activator activity	6/41	6	43/743	0.025604938	0.267022925	0.25027383	6
MF	GO:0048018	receptor ligand activity	6/41	6	43/743	0.025604938	0.267022925	0.25027383	6
MF	GO:0004888	transmembrane signaling receptor activity	9/41	9	85/743	0.0349037	0.318496267	0.298518491	9

Supplement 7. GO enrichment analysis of DEGs in NID vs HC.

ONTOLOGY	ID	Description	GeneRatio	count	BgRatio	pvalue	p.adjust	qvalue	Count
BP	GO:0002376	immune system process	13/70	13	44/559	0.001364059	0.145341077	0.142239916	13
BP	GO:0002682	regulation of immune system process	7/70	7	16/559	0.001634369	0.145341077	0.142239916	7
