# Supplementary material for: Integrated transcriptomic and metabolomic analyses reveal host–metabolite interactions underlying cloacal inflammation in laying Pekin ducks
Source: Anim Biosci. 2026 Apr 16;39(10):260208. doi: 10.5713/ab.260208 (PMC13662949; doi:10.5713/ab.260208)
Supplement: Supplementary file 11 [file ab-260208-Supplementary-8.pdf]

BP	GO:0006955	immune response	12/70	12	41/559	0.002356882	0.145341077	0.142239916	12
BP	GO:0048518	positive regulation of biological process	10/70	10	36/559	0.008590445	0.397308085	0.388830672	10
BP	GO:0002684	positive regulation of immune system process	5/70	5	13/559	0.01538954	0.525755505	0.514537393	5
BP	GO:0006954	inflammatory response	5/70	5	15/559	0.029334107	0.525755505	0.514537393	5
BP	GO:0007264	small GTPase mediated signal transduction	5/70	5	15/559	0.029334107	0.525755505	0.514537393	5
BP	GO:0002833	positive regulation of response to biotic stimulus	4/70	4	11/559	0.037597083	0.525755505	0.514537393	4
BP	GO:0031349	positive regulation of defense response	4/70	4	11/559	0.037597083	0.525755505	0.514537393	4
BP	GO:0032103	positive regulation of response to external stimulus	4/70	4	11/559	0.037597083	0.525755505	0.514537393	4
BP	GO:0045088	regulation of innate immune response	4/70	4	11/559	0.037597083	0.525755505	0.514537393	4
BP	GO:0045089	positive regulation of innate immune response	4/70	4	11/559	0.037597083	0.525755505	0.514537393	4
BP	GO:0006952	defense response	7/70	7	27/559	0.040233003	0.525755505	0.514537393	7

Supplement 8. KEGG pathway enrichment of DEGs in ID vs NID.

subcategory	ID	Description	GeneRatio	BgRatio	pvalue	p.adjust	qvalue	Count
NA	apla04820	Cytoskeleton in muscle cells	56/295	215/5816	4.14E-26	5.09E-24	4.61E-24	56
Signaling molecules and interaction	apla04060	Cytokine-cytokine receptor interaction	31/295	205/5816	2.80E-08	1.72E-06	1.56E-06	31
Cell motility	apla04814	Motor proteins	26/295	184/5816	1.51E-06	6.18E-05	5.60E-05	26
Immune system	apla04672	Intestinal immune network for IgA production	10/295	34/5816	4.28E-06	0.000131675	0.000119449	10
Signal transduction	apla04020	Calcium signaling pathway	26/295	235/5816	0.000124328	0.003058461	0.002774471	26
Immune system	apla04620	Toll-like receptor signaling pathway	13/295	84/5816	0.000272748	0.005321399	0.004827285	13
Immune system	apla04625	C-type lectin receptor signaling pathway	14/295	96/5816	0.000302844	0.005321399	0.004827285	14
Circulatory system	apla04261	Adrenergic signaling in cardiomyocytes	16/295	125/5816	0.000523124	0.008043025	0.007296197	16
Circulatory system	apla04260	Cardiac muscle contraction	10/295	65/5816	0.00144372	0.019730844	0.017898754	10
Transport and catabolism	apla04145	Phagosome	15/295	133/5816	0.002827115	0.034773513	0.031544651	15
Transport and catabolism	apla04148	Efferocytosis	15/295	146/5816	0.006822955	0.075232503	0.068246858	15
Signaling molecules and interaction	apla04512	ECM-receptor interaction	10/295	81/5816	0.007412607	0.075232503	0.068246858	10
Signal transduction	apla04371	Apelin signaling pathway	13/295	121/5816	0.007951403	0.075232503	0.068246858	13
Infectious disease: bacterial	apla05132	Salmonella infection	19/295	221/5816	0.016202432	0.142349937	0.129132164	19
Signaling molecules and interaction	apla04514	Cell adhesion molecules	12/295	125/5816	0.024063462	0.197320385	0.178998381	12
Infectious disease: viral	apla05164	Influenza A	12/295	129/5816	0.029891453	0.229790548	0.208453556	12
Endocrine system	apla04920	Adipocytokine signaling pathway	7/295	61/5816	0.033557964	0.24280174	0.220256606	7

Supplement 9. KEGG pathway enrichment of DEGs in ID vs NID.

subcategory	ID	Description	GeneRatio	BgRatio	pvalue	p.adjust	qvalue	Count
Signaling molecules and interaction	apla04060	Cytokine-cytokine receptor interaction	26/208	205/5816	1.18E-08	1.32E-06	1.25E-06	26
Signaling molecules and interaction	apla04512	ECM-receptor interaction	13/208	81/5816	4.71E-06	0.000255424	0.000242461	13
NA	apla04820	Cytoskeleton in muscle cells	22/208	215/5816	6.84E-06	0.000255424	0.000242461	22
NA	apla04382	Cornified envelope formation	15/208	137/5816	9.86E-05	0.002759915	0.002619845	15
Cellular community - eukaryotes	apla04510	Focal adhesion	15/208	185/5816	0.002416197	0.053983608	0.051243839	15
Immune system	apla04620	Toll-like receptor signaling pathway	9/208	84/5816	0.002891979	0.053983608	0.051243839	9
Immune system	apla04672	Intestinal immune network for IgA production	5/208	34/5816	0.006657356	0.106517698	0.101111725	5
