# Supplementary material for: Integrated transcriptomic and metabolomic analyses reveal host–metabolite interactions underlying cloacal inflammation in laying Pekin ducks
Source: Anim Biosci. 2026 Apr 16;39(10):260208. doi: 10.5713/ab.260208 (PMC13662949; doi:10.5713/ab.260208)
Supplement: Supplementary file 12 [file ab-260208-Supplementary-9.pdf]

BP	GO:0006955	immune response	12/70	12	41/559	0.002356882	0.145341077	0.142239916	12
BP	GO:0048518	positive regulation of biological process	10/70	10	36/559	0.008590445	0.397308085	0.388830672	10
BP	GO:0002684	positive regulation of immune system process	5/70	5	13/559	0.01538954	0.525755505	0.514537393	5
BP	GO:0006954	inflammatory response	5/70	5	15/559	0.029334107	0.525755505	0.514537393	5
BP	GO:0007264	small GTPase mediated signal transduction	5/70	5	15/559	0.029334107	0.525755505	0.514537393	5
BP	GO:0002833	positive regulation of response to biotic stimulus	4/70	4	11/559	0.037597083	0.525755505	0.514537393	4
BP	GO:0031349	positive regulation of defense response	4/70	4	11/559	0.037597083	0.525755505	0.514537393	4
BP	GO:0032103	positive regulation of response to external stimulus	4/70	4	11/559	0.037597083	0.525755505	0.514537393	4
BP	GO:0045088	regulation of innate immune response	4/70	4	11/559	0.037597083	0.525755505	0.514537393	4
BP	GO:0045089	positive regulation of innate immune response	4/70	4	11/559	0.037597083	0.525755505	0.514537393	4
BP	GO:0006952	defense response	7/70	7	27/559	0.040233003	0.525755505	0.514537393	7

Supplement 8. KEGG pathway enrichment of DEGs in ID vs NID.

subcategory	ID	Description	GeneRatio	BgRatio	pvalue	p.adjust	qvalue	Count
NA	apla04820	Cytoskeleton in muscle cells	56/295	215/5816	4.14E-26	5.09E-24	4.61E-24	56
Signaling molecules and interaction	apla04060	Cytokine-cytokine receptor interaction	31/295	205/5816	2.80E-08	1.72E-06	1.56E-06	31
Cell motility	apla04814	Motor proteins	26/295	184/5816	1.51E-06	6.18E-05	5.60E-05	26
Immune system	apla04672	Intestinal immune network for IgA production	10/295	34/5816	4.28E-06	0.000131675	0.000119449	10
Signal transduction	apla04020	Calcium signaling pathway	26/295	235/5816	0.000124328	0.003058461	0.002774471	26
Immune system	apla04620	Toll-like receptor signaling pathway	13/295	84/5816	0.000272748	0.005321399	0.004827285	13
Immune system	apla04625	C-type lectin receptor signaling pathway	14/295	96/5816	0.000302844	0.005321399	0.004827285	14
Circulatory system	apla04261	Adrenergic signaling in cardiomyocytes	16/295	125/5816	0.000523124	0.008043025	0.007296197	16
Circulatory system	apla04260	Cardiac muscle contraction	10/295	65/5816	0.00144372	0.019730844	0.017898754	10
Transport and catabolism	apla04145	Phagosome	15/295	133/5816	0.002827115	0.034773513	0.031544651	15
Transport and catabolism	apla04148	Efferocytosis	15/295	146/5816	0.006822955	0.075232503	0.068246858	15
Signaling molecules and interaction	apla04512	ECM-receptor interaction	10/295	81/5816	0.007412607	0.075232503	0.068246858	10
Signal transduction	apla04371	Apelin signaling pathway	13/295	121/5816	0.007951403	0.075232503	0.068246858	13
Infectious disease: bacterial	apla05132	Salmonella infection	19/295	221/5816	0.016202432	0.142349937	0.129132164	19
Signaling molecules and interaction	apla04514	Cell adhesion molecules	12/295	125/5816	0.024063462	0.197320385	0.178998381	12
Infectious disease: viral	apla05164	Influenza A	12/295	129/5816	0.029891453	0.229790548	0.208453556	12
Endocrine system	apla04920	Adipocytokine signaling pathway	7/295	61/5816	0.033557964	0.24280174	0.220256606	7

Supplement 9. KEGG pathway enrichment of DEGs in ID vs NID.

subcategory	ID	Description	GeneRatio	BgRatio	pvalue	p.adjust	qvalue	Count
Signaling molecules and interaction	apla04060	Cytokine-cytokine receptor interaction	26/208	205/5816	1.18E-08	1.32E-06	1.25E-06	26
Signaling molecules and interaction	apla04512	ECM-receptor interaction	13/208	81/5816	4.71E-06	0.000255424	0.000242461	13
NA	apla04820	Cytoskeleton in muscle cells	22/208	215/5816	6.84E-06	0.000255424	0.000242461	22
NA	apla04382	Cornified envelope formation	15/208	137/5816	9.86E-05	0.002759915	0.002619845	15
Cellular community - eukaryotes	apla04510	Focal adhesion	15/208	185/5816	0.002416197	0.053983608	0.051243839	15
Immune system	apla04620	Toll-like receptor signaling pathway	9/208	84/5816	0.002891979	0.053983608	0.051243839	9
Immune system	apla04672	Intestinal immune network for IgA production	5/208	34/5816	0.006657356	0.106517698	0.101111725	5

Immune system	apla04625	C-type lectin receptor signaling pathway	8/208	96/5816	0.0209601	0.293441403	0.278548701	8
Signaling molecules and interaction	apla04514	Cell adhesion molecules	9/208	125/5816	0.034369189	0.416065574	0.394949464	9
Lipid metabolism	apla00590	Arachidonic acid metabolism	5/208	52/5816	0.037148712	0.416065574	0.394949464	5
Transport and catabolism	apla04145	Phagosome	9/208	133/5816	0.048040547	0.489140117	0.464315336	9

Supplement 10. KEGG pathway enrichment of DEGs in ID vs NID.

subcategory	ID	Description	GeneRatio	BgRatio	pvalue	p.adjust	qvalue	Count
Immune system	apla04672	Intestinal immune network for IgA production	20/439	34/5816	1.23E-14	1.77E-12	1.68E-12	20
NA	apla04820	Cytoskeleton in muscle cells	47/439	215/5816	1.00E-11	7.20E-10	6.84E-10	47
Signaling molecules and interaction	apla04514	Cell adhesion molecules	28/439	125/5816	1.08E-07	5.17E-06	4.91E-06	28
Signaling molecules and interaction	apla04060	Cytokine-cytokine receptor interaction	37/439	205/5816	3.82E-07	1.37E-05	1.31E-05	37
Cell motility	apla04814	Motor proteins	31/439	184/5816	1.51E-05	0.000433495	0.000411947	31
Transport and catabolism	apla04145	Phagosome	23/439	133/5816	0.000127973	0.00307136	0.00291869	23
Circulatory system	apla04260	Cardiac muscle contraction	14/439	65/5816	0.000269976	0.00555379	0.005277725	14
Circulatory system	apla04261	Adrenergic signaling in cardiomyocytes	19/439	125/5816	0.002410388	0.043386983	0.04123032	19
Cell growth and death	apla04110	Cell cycle	20/439	138/5816	0.003324249	0.053187987	0.05054414	20
Immune system	apla04625	C-type lectin receptor signaling pathway	15/439	96/5816	0.00514508	0.074089156	0.070406362	15
Replication and repair	apla03030	DNA replication	7/439	30/5816	0.005892367	0.077136438	0.07330217	7
Signal transduction	apla04020	Calcium signaling pathway	26/439	235/5816	0.030010945	0.360131341	0.342230075	26
Energy metabolism	apla00910	Nitrogen metabolism	4/439	18/5816	0.041963539	0.452454549	0.429964118	4
Infectious disease: viral	apla05168	Herpes simplex virus 1 infection	16/439	134/5816	0.043988637	0.452454549	0.429964118	16

Supplement 11. Classification of detected metabolites in the cloacal contents of diseased and healthy ducks.

Metabolite Set	Total	Hits	Ratio	Expect	P value	Holm P	FDR
Organic acids and derivatives	4600	219	23.77850163	20.4	4.98E-154	1.74E-152	1.74E-152
Organoheterocyclic compounds	6560	173	18.78393051	29	8.49E-80	2.89E-78	1.49E-78
Organic oxygen compounds	3240	81	8.794788274	14.4	2.65E-35	8.74E-34	3.09E-34
Benzenoids	5030	86	9.337676439	22.3	6.03E-26	1.93E-24	5.28E-25
Nucleosides, nucleotides, and analogues	560	27	2.931596091	2.48	1.59E-19	4.91E-18	1.11E-18
Organic nitrogen compounds	632	18	1.954397394	2.8	9.1E-10	2.73E-08	5.31E-09
Phenylpropanoids and polyketides	3830	35	3.800217155	17	0.0000665	0.00193	0.000332
Alkaloids and derivatives	442	8	0.868621064	1.96	0.000903	0.0253	0.00395
Hydrocarbons	283	4	0.434310532	1.25	0.038	1	0.148
Hydrocarbon derivatives	14	1	0.108577633	0.0619	0.0602	1	0.211
Lignans, neolignans and related compounds	217	1	0.108577633	0.96	0.618	1	1
Lipids and lipid-like molecules	94100	268	29.09880565	416	1	1	1
Sum		921					
